# Catalyst-Solvent System for PASE Approach to Hydroxyquinolinone-Substituted Chromeno[2,3-*b*]pyridines Its Quantum Chemical Study and Investigation of Reaction Mechanism

**DOI:** 10.3390/molecules25112573

**Published:** 2020-05-31

**Authors:** Fedor V. Ryzhkov, Yuliya E. Ryzhkova, Michail N. Elinson, Stepan V. Vorobyev, Artem N. Fakhrutdinov, Anatoly N. Vereshchagin, Mikhail P. Egorov

**Affiliations:** 1 N. D. Zelinsky Institute of Organic Chemistry Russian Academy of Sciences, Leninsky pr. 47, 119991 Moscow, Russia; ryzhkov.fe@ya.ru (F.V.R.); julia4912@mail.ru (Y.E.R.); fak.part@gmail.com (A.N.F.); anatoly103@yandex.ru (A.N.V.); mpe@ioc.ac.ru (M.P.E.); 2 Department of Organic Chemistry and Petroleum Chemistry, Gubkin Russian State University of Oil and Gas, 65 Leninsky Prospect, 119991 Moscow, Russia; vorstepan@yandex.ru

**Keywords:** salicylaldehyde, malononitrile dimer, hydroxyquinolinone, chromeno[2,3-*b*]pyridines, multicomponent reactions, PASE approach, corrosion inhibitor, NMR monitoring

## Abstract

The Pot, Atom, and Step Economy (PASE) approach is based on the Pot economy principle and unites it with the Atom and Step Economy strategies; it ensures high efficiency, simplicity and low waste formation. The PASE approach is widely used in multicomponent chemistry. This approach was adopted for the synthesis of previously unknown hydroxyquinolinone substituted chromeno[2,3-*b*]pyridines via reaction of salicylaldehydes, malononitrile dimer and hydroxyquinolinone. It was shown that an ethanol-pyridine combination is more beneficial than other inorganic or organic catalysts. Quantum chemical studies showed that chromeno[2,3-*b*]pyridines has potential for corrosion inhibition. Real time ^1^H NMR monitoring was used for the investigation of reaction mechanism and 2-((2*H*-chromen-3-yl)methylene)malononitrile was defined as a key intermediate in the reaction.

## 1. Introduction

Multicomponent reactions (MCRs) employ three or more reactants to obtain heterocycles containing structures of all starting materials in a one-pot process under fixed reaction conditions [1,2,3,4]. It provides powerful productivity to satisfy modern green chemistry requirements, but new synthetic strategies with robust efficiency are still demanded. In this connection, the PASE approach has recently emerged [5,6], it is based on the Pot economy principle and unites it with the Atom and Step Economy strategies (PASE), thereby ensuring high efficiency, simplicity and low waste formation [7,8,9,10]. Nowadays, it is emerging as a fast-paced research front of organic chemistry [11,12,13,14,15,16].

Corrosion is a gradual destruction of refined metal by means of reactions with environment. Today it causes heavy losses to the economy [17,18,19]. Due to electronic configuration of heterocyclic compounds, they are capable of corrosion inhibition or coating metals. The development of PASE approaches to anti-corrosion heterocycles is beneficial for protection of metals.

Chromeno [2,3-*b*]pyridines are heterocycles with special and useful electronic configuration. Thus, they showed gastric antisecretory activity [20], inhibit mitogen-activated protein kinase 2 and suppress expression of tumor necrosis factor alpha (TNFα) in U937 cells [21].

Similar compounds were earlier synthesized via microwave irradiation of aldehydes, malononitrile dimer and kojic acid in EtOH [22]. The synthesis was carried out under reflux conditions in the presence of Et_3_N as a catalyst (73–90% yields). Bis-chromeno[2,3-*b*]pyridine derivatives were obtained via reaction of bis-aldehydes, malononitrile dimer and dimedone under reflux conditions. The reaction was carried out for 5 h in EtOH with a large amount of piperidine [23]. Phenyl substituted derivatives were obtained by reaction of benzaldehydes, malononitrile dimer and naphthols (12%–62% yields). The reaction was carried out under reflux conditions in H_2_O-EtOH with an equivalent amount of Et_2_NH [24] and in solvent-free conditions with guanidine hydrochloride catalysis (100 °C, 2 h) [25]. Solvent-free conditions were also used for the transformation of benzaldehydes, malononitrile dimer and coumarin [26]. Carbazole and indole derivatives were synthesized (67–85%) under reflux conditions and microwave irradiation: by reaction of aldehydes, malononitrile dimer and hydroxycarbazole or hydroxyindole in anhydrous EtOH with EtONa [27].

We have already described multicomponent syntheses of chromeno[2,3-*b*]pyridines [28,29,30,31,32,33,34]. However, there are no reports on a PASE synthesis of hydroxyquinolinone substituted chromeno[2,3-*b*]pyridines. Therefore, our attention was devoted toward reactions of salicylaldehydes, malononitrile dimer and hydroxyquinolinone. We were interested in developing the PASE method, clarifying reaction mechanism and defining the actual intermediates of the reaction. We were also prompted to carry out quantum chemical studies and estimate chromeno[2,3-*b*]pyridine potential for corrosion inhibition.

## 2. Results and Discussion

### 2.1. PASE Approach

**Scheme 1:**
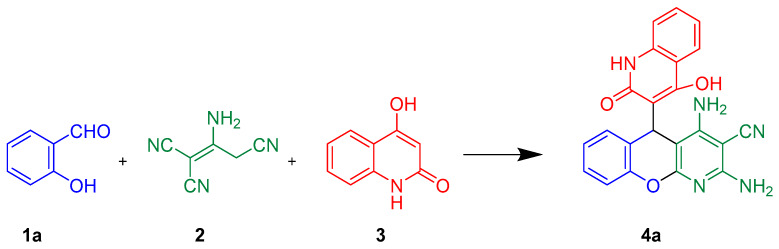
Reaction of salicylaldehyde, malononitrile dimer and hydroxyquinolinone.

Initially, to examine the reaction of salicylaldehyde **1a**, malononitrile dimer **2** and hydroxyquinolinone **3**, we have carried out multicomponent synthesis of chromeno[2,3-*b*]pyridine **4a** under solvent-free conditions (Scheme 1, Table 1). Previously, AcONa and KF have successfully catalyzed similar processes under solvent-free conditions [32]. AcONa showed better results (Entry 3) than KF (Entry 2) and provided twice the yield as the reaction without catalyst (Entry 1). ‘On-water’ [33,34,35] reactions showed almost the same results as solvent-free experiments (Entries 4,5).

Further, we have examined the multicomponent reaction in alcohol media. The reaction in EtOH without catalyst resulted in assembling of chromeno[2,3-*b*]pyridine **4a** in 19% yield (Entry 6), in the presence of AcONa it was formed in 35% yield (Entry 7) whereas organocatalysis by Et_3_N and morpholine resulted in 63% and 57% yields (Entries 8 and 9).

Pyridine (Py) has been already employed as a solvent and catalyst for similar processes [36]. Refluxing of salicylaldehyde **1a**, malononitrile dimer **2** and hydroxyquinone **3** in Py for 2 h resulted in 61% yield (Entry 10). Py is a good solvent for structures of this type, however, a small amount of chromeno[2,3-*b*]pyridine **4a** always remains dissolved in Py.

Then, reaction in a mixture of EtOH and Py was carried out. In the volumetric range of EtOH:Py (2-4:1), the best yield was in EtOH-Py (3:1) and chromeno[2,3-*b*]pyridine **4a** was isolated in excellent 95% yield (Entry 12).

**Figure 1:**
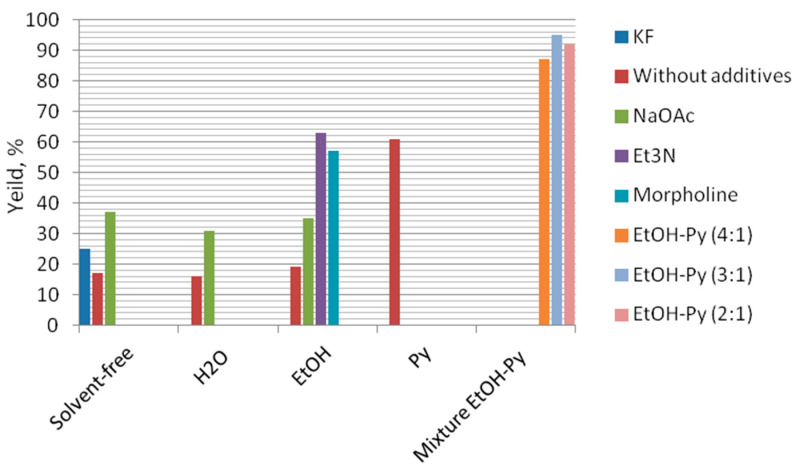
Comparison diagram of approaches to chromeno[2,3-*b*]pyridine **4a**. The mixture of EtOH-Py (3:1) provides the best result.

Figure 1 shows that this organic catalyst is more effective than inorganic KF or AcONa catalysts and the ‘EtOH-Py’ system is more beneficial than ‘solvent-free’ and ‘on-water’ approaches to chromeno[2,3-*b*]pyridine **4a**. Py in EtOH keeps the intermediates dissolved and ensures optimal basicity [37] supporting the reaction.

When the reaction in EtOH:Py mixture was finished, the final compound was directly crystallized in pure form after cooling. Under the optimal conditions (Entry12: refluxing for 2 h in 4 mL of EtOH-Py (3:1) mixture) multicomponent reactions of salicylaldehydes **1a**–**i**, malononitrile dimer **2** and hydroxyquinolinone **3** were carried out. Chromeno[2,3-*b*]pyridines **4a**–**i** were obtained in 59–98% yields (Table 2). In general, substitution reduced yields of chromeno[2,3-*b*]pyridines **4b**–**i**. Presumably, electronic effects were taking place: electron-donating methyl, methoxy and ethoxy groups tended to support higher yields than bromo and nitro groups. The yield of chlorine substituted compound was bigger than in the case of bromo substituted compound due to a mesomeric effect.

**Scheme 2:**
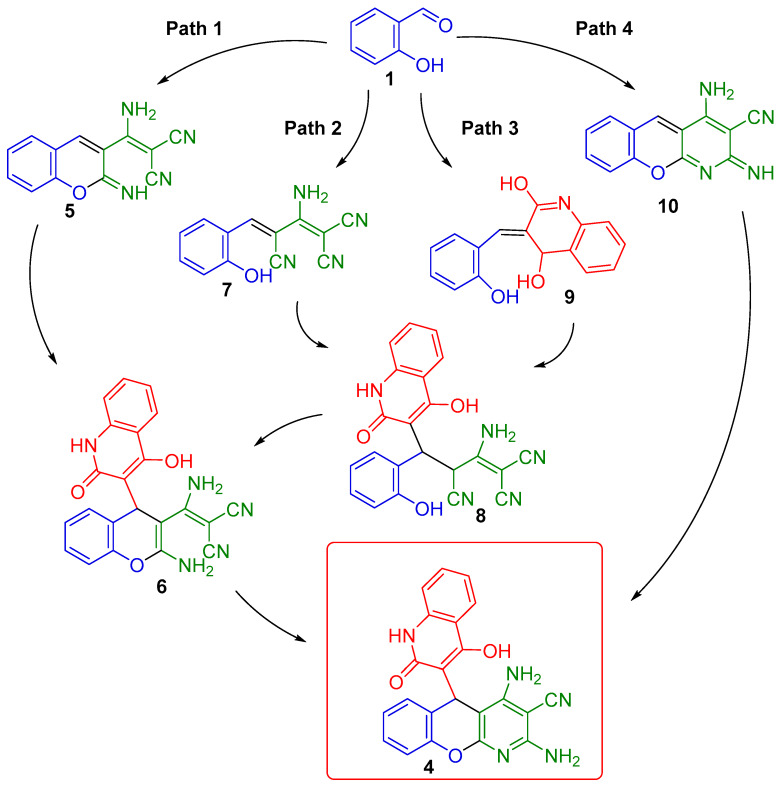
The key intermediates for the possible pathways to chromeno[2,3-*b*]pyridine formation.

Considering the mechanism of this multicomponent reaction, several path-ways for the reaction were possible. Salicylaldehyde **1** may have undergone condensation either with malononitrile dimer **2** or hydroxyquinolinone **3** (Scheme 2). Hence, four main pathways of the reaction could be discussed. Path 1 started from Knoevenagel condensation of salicylaldehyde **1** and malononitrile dimer **2**, then cyclization into intermediate **5** proceeded. The cyclization was followed by Michael addition of hydroxyquinolinone **3** and another cyclization. Path 2 [38,39] started from Knoevenagel condensation of salicylaldehyde **1** and malononitrile dimer **2**. Further Michael addition of hydroxyquinolinone **3** to intermediate **7** took place, and subsequent double cyclization of the malononitrile fragment formed chromeno[2,3-*b*]pyridine **4**. Path 3 began with Knoevenagel condensation of salicylaldehyde **1** and hydroxyquinolinone **3**. Formation of chromeno[2,3-*b*]pyridine **4** proceeded via intermediate **8**. In Path 4, salicylaldehyde **1** undergoes condensation with malononitrile dimer **2**, and then double cyclization took place to form intermediate **10**. Further, addition of hydroxyquinolinone **3** formed chromeno[2,3-*b*]pyridine **4**.

In order to gain insight into the reaction mechanism, we have performed ^1^H NMR monitoring, to obtain constituent data on the reaction. To reduce the influence of sample preparation, the transformation of starting materials into chromeno[2,3-*b*]pyridine **4a** was carried out and monitored directly into a spectrometer without catalyst to slow down the reaction.

**Figure 2:**
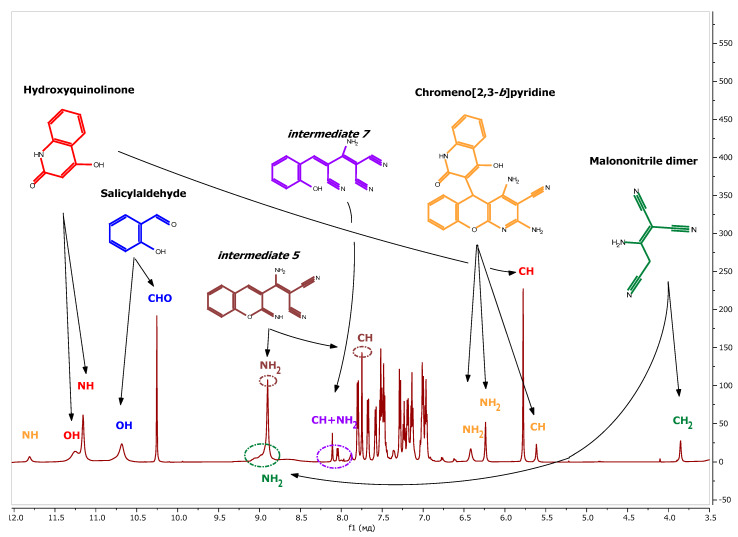
Representative ^1^H NMR spectrum of multicomponent reaction in DMSO-*d*_6_ at 313 K recorded 2 h after dissolution.

During the NMR study, six major components were recorded: salicylaldehyde **1a**, malononitrile dimer **2** and hydroxyquinolinone **3**, intermediate **5**, intermediate **7** and chromeno[2,3-*b*]pyridine **4a**. A representative ^1^H NMR spectrum with assignment of peaks showed on Figure 2.

The starting materials are well known (we estimated the presence of salicylaldehyde **1a** by the singlet at 10.25 ppm, malononitrile dimer **2** by the singlet at 3.85 ppm and hydroxyquinolinone by the singlet at 5.78 ppm); compound **4a** is described in this manuscript and was estimated by the signal at 5.61 ppm; intermediate **5** was described previously (7.75 ppm) [40]; intermediate **7** was estimated by the signals at 8.04–8.11 ppm [41].

**Figure 3:**
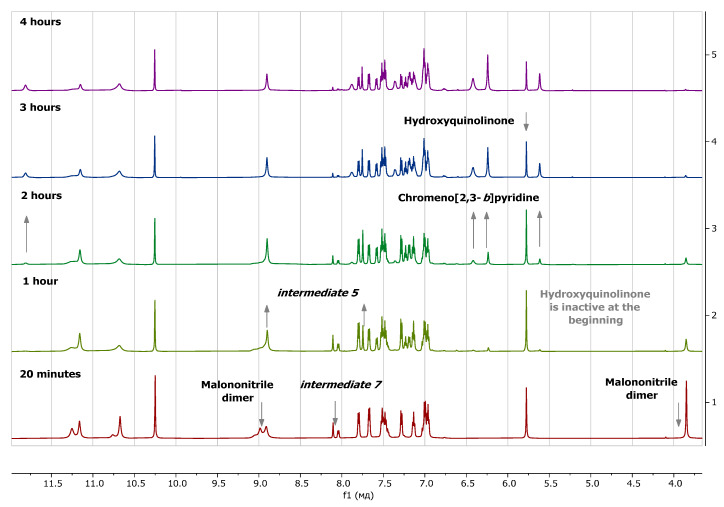
Real-time monitoring of chemical reaction by ^1^H NMR spectroscopy in DMSO-*d*_6_ at 313 K. The NMR spectra of all the synthesized compounds, as well as the NMR spectra of the real-time monitoring are presented in Supplementary Materials (Figures S1–S42).

Figure 3 presents a series of ^1^H NMR spectra for a conversion of starting materials into the final compound **4a** in DMSO-*d*_6_. It shows the disappearance of malononitrile dimer **2** (in 20 min to 2 h from the beginning of experiment) and the simultaneous formation of intermediate **7** and intermediate **5.** Intermediate **7** formed by Knoevenagel condensation and it was followed by cyclization into intermediate **5** (Scheme 2). Thus, intermediate **7** was forming and rapidly converting into intermediate **5** (1–3 h of experiment). Eventually the signals of intermediate **7** disappeared.

As shown in Figure 3, when intermediate **7** disappeared (Figure 3), hydroxyquinolinone **3** signals disappeared and chromeno[2,3-*b*]pyridine **4a** signals appeared. Thus, hydroxyquinolinone **3** took part in Michael addition to the electron-deficient intermediate **5** to form final compound **4a**.

Based on these data and taking into consideration earlier published results [42,43], we suggest that the first stage was a rapid formation of intermediate **7** with expulsion of a hydroxide anion [37]. This hydroxide anion instantly catalyzed a rapid cyclization of intermediate **7** into intermediate **5**. Then, subsequent Michael addition and cyclization formed the chromeno[2,3-*b*]pyridine **4a**.

To prove this conclusion, the intermediate **5** was isolated in a one-step reaction. Further addition of hydroxyquinolinone **3** to the obtained intermediate **5** in a distinct reactor afforded chromeno[2,3-*b*]pyridine **4a**. The reaction conditions were the same as developed for multicomponent process and the yield of chromeno[2,3-*b*]pyridine **4a** was 95% again. More than that, the two-component reaction of salicylaldehyde **1a** and hydroxyquinolinone **3** in a separate reactor did not form intermediate **9**. This experimental data and ^1^NMR monitoring data were consistent with each other. Thus, the results from both intermediate synthesis and ^1^NMR monitoring turned out to be consistent with each other.

Moreover, both ^1^NMR monitoring and experimental data (Table 1 and Table 2) revealed no signals of intermediate **9 [44]**: 7.11–7.16 (m, 1H), 7.36 (s, 1H), 7.54–7.58 (m, 1H), 7.67–7.70 (d, 1H, *J* = 8.4 Hz), 7.93–7.99 (m, 4H), 8.45–8.48 (d, 1H, *J* = 8.4 Hz), 10.22 (s, 1H), 11.19 (s, 1H) ppm.

**Scheme 3:**
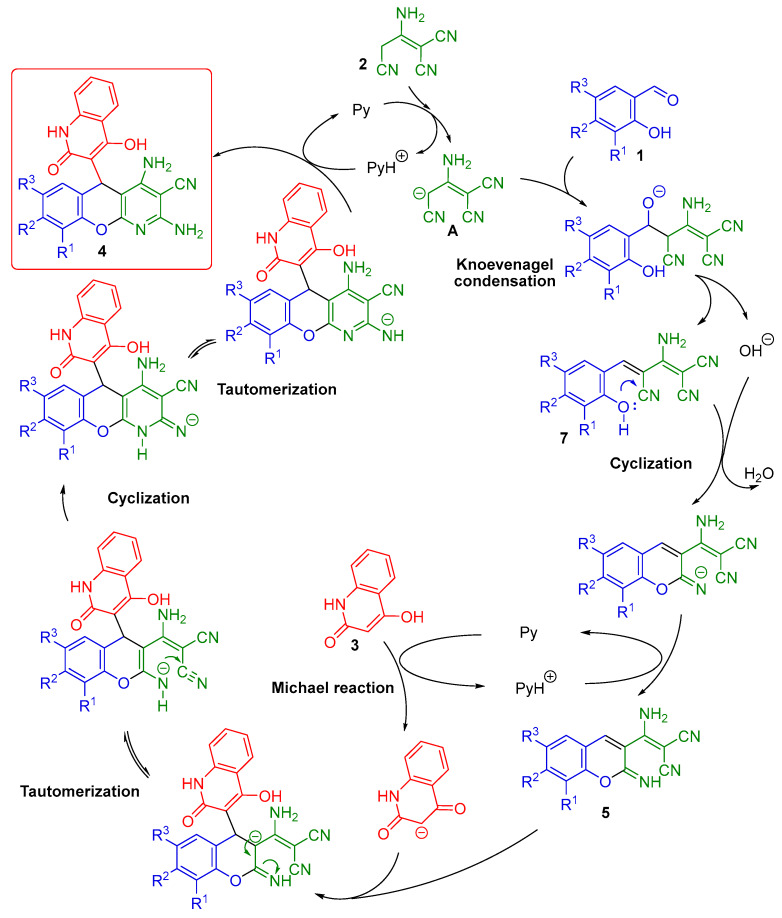
Mechanism of salicylaldehydes **1**, malononitrile dimer **2** and hydroxyquinolinone **3** transformation into chromeno[2,3-*b*]pyridine **4.** Catalytic cycles are simplified.

To sum up, we postulated Path 1 for the assembling of chromeno[2,3-*b*]pyridine **4a** in this multicomponent process. The proposed mechanism for the developed reaction is outlined in Scheme 3.

### 2.2. Quantum Chemical Investigation

#### 2.2.1. Quantum Chemistry Approach

One of the most well-known corrosion inhibitors are heterocycles, such as azoles [45,46], pyridines [47], several bioorganic compounds [48] and chromenes [49]. The mechanism of their action is the adsorption on the metal’s surface (whatever metal is used–copper, steel, brass, etc.). Generally, the greater the number of electron-donating groups in molecule, the higher the probability of adsorption and inhibition activity. As it was shown previously, the inhibitory activity of organic compounds can be predicted properly by quantum-chemical methods [50,51,52]. This reduces the time of the study and hazardous reactant wasting by selecting the hit-compounds. They must have appropriate electronic properties, which can be estimated by quantum chemistry calculations, to interact with metal’s surface.

To estimate electronic properties, the next method can be used [53]: according to Koopman’s theorem, the energy of the highest occupied orbital (*E_HOMO_*) is equal to the ionization potential (*I*) having the opposite charge, the energy of the lowest unoccupied orbital (*E_LUMO_*) is related to the electron affinity (*A*).

Considering the quantum chemical parameters of the inhibitor, the higher the energy of the highest occupied orbital (HOMO), the higher the donation of the inhibitor to metal’s vacant d-orbital; the lower by the energy of the lowest unoccupied orbital (LUMO), the greater is the electron acception from the metal to inhibitor. The electron release is characterized by energy difference (Δ*E_(L-H)_*). The electron release is easier when the Δ*E_(L-H)_* is lower, and then the adsorption of inhibitor is stronger [54]. The electronic density distribution is an important parameter as well, and it can be estimated by a frontier orbital localization study and partial atomic charge distribution [55].

In addition to HOMO and LUMO, several significant parameters were calculated as well. Electronegativity shows the ability of the molecule to attract electrons towards itself and therefore a molecule with the lowest value tends to has the highest ability to donate electrons.

The global chemical hardness is related to the resistance of a molecule to charge transfer [56]. The global chemical softness (σ) is inversely proportional from the chemical hardness. The higher softness, the better adsorption [57]. Chemical hardness (η) as well as electronegativity (χ) can be calculated from ionization potential and electron affinity, and then chemical softness can be calculated from chemical hardness. Since the molecule of inhibitor must donate its electron to vacant metal orbitals, its nucleophilicity should be high, opposite to electrophilicity and hence to the global electrophilicity index (ω) [58].

All quantum chemical calculations were performed using the Gaussian09 program package [59]. The structures **4a**–**i** were optimized and the required parameters were calculated using the density functional theory method B3LYP [60] with the 6-311G(d,p) basic set. This method is recommended for calculation of frontier orbitals energies and related values, such as electronegativity, polarizability etc. [61]. Our study was carried out for molecules in gas phase as well as for solvated ones in water (PCM model) [62].

The results of quantum chemical calculations for gas phase, solvated forms and protonated forms are presented in Table 3, frontier orbitals of several studied compounds are shown in Table 4.

#### 2.2.2. Gas Phase Calculations

**Figure 4:**
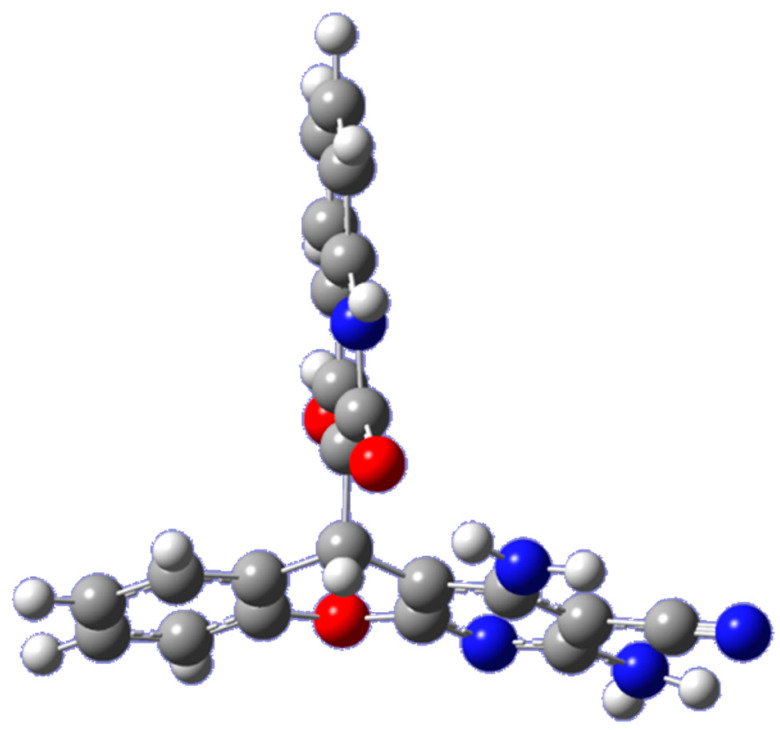
Optimized structure for compound **4a** (gas phase).

During quantum chemical calculations, the structures of the studied compounds were optimized as shown in Figure 4 for **4a**. The chromeno[2,3-*b*]pyridine fragment was not planar and hydroxyquinolinone ring was localized nearly perpendicular to chromeno[2,3-*b*]pyridine ring. Calculated key parameters of the studied compounds **4a**–**i** are shown in Table 3.

As mentioned above, corrosion inhibition depends on the energies and distributions of frontier orbitals. For most cases in considered compounds, the HOMOs are localized on the benzene ring atoms of chromeno[2,3-*b*]pyridines, making them sites for interaction with metal cations that formed by dissolving the metal in acid. The LUMOs are localized in the hydroxyquinolinone ring of the substituent. Such a type of distribution of the frontier orbitals is represented in compounds **4a, 4b, 4d** and **4i**. The frontier orbitals of several compounds are shown in Table 4.

Introducing of a strong electron-withdrawing substituent, such as a nitro-group, tended to change the orbital distribution. The HOMO became localized on the atoms of the pyridine ring, and the LUMO on the nitro-group of the substituent (**4h**, Table 4). A similar distribution was observed for the compounds **4e**, **4f** and **4g**: the HOMOs were localized in the pyridine ring, while the LUMOs were in the hydroxyquinolinone ring (Table 4). This was caused by the withdrawing effect of the halogen atom. It is noteworthy that compound **4c** had the same distribution of the orbitals, which was probably related to poor overlapping of oxygen orbital of methoxy-group with ones of benzene ring.

The energy difference is another important parameter. The lower the energy difference of the molecule, the higher the reaction ability (due to easier electron release). For the studied compounds these values were comparable, but **4b**, **4e** and **4i** had the lowest ones. The global electronegativity χ values for **4b**, **4e** and **4i** were almost twice as low as values calculated for similar tricyclic cationic inhibitors (<7.2 is accepted, [53]). The global electrophilicity index ω was low as well. It was almost five times lower than the values calculated for the studied inhibitors (<19.3 is accepted, [53]).

#### 2.2.3. Solvated Form Calculations

Several parameters changed by taking into consideration the solvation processes. Thus, the frontier orbitals in compounds **4a**, **4b**, **4d**, **4f** and **4i** were located in the same manner as in non-solvated ones, but for **4c**, **4e**, **4g** and **4h** shifting of the HOMOs to the carbonyl atoms of hydroxyquinolinone ring is observed, while the LUMOs remained in the same place (Table 4).

The analysis of the basic parameters (Table 3) shows that assuming solvation led to changing of some values mostly for compound **4h**; we can expect the highest anticorrosive effect from **4b**, **4c** and **4d** due to their low values of general electrophilicity ω, which can be predicted because of electron-donating substituents. The values of electronegativity χ for **4b**, **4c** and **4d** remained low, almost the same as was calculated for the gas phase. It is noteworthy that the value of the global softness σ for these compounds tended to decrease while taking to consideration the solvation effects.

#### 2.2.4. Protonated form Calculations

In the case of a corrosion inhibition study in high acidic media, we must take in consideration the possibility of protonation of our compounds because of the basicity of some functional groups.

**Scheme 4:**
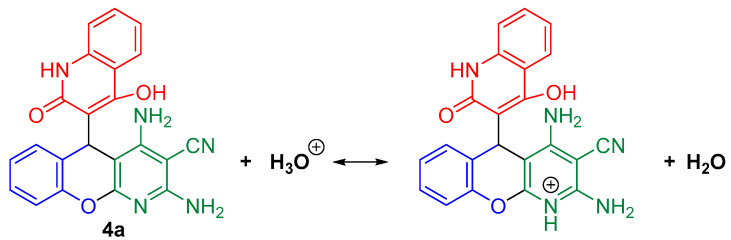
Protonation of chromeno[2,3-*b*]pyridine **4a**.

As it was shown previously [63], the main center of basicity in aminopyridines is the nitrogen atom in heterocyclic ring. We decided to confirm this statement by calculating the protonation energy for the compound **4a** (Scheme 4).

Proton affinity (PA) is Equation:

PA = *E*_PyH_ + *E*_H_2_O_ − *E*_Py_ − *E*_H_3_O^+^_ (1)

where E_i_–the total energy of the corresponding compound

We found that for the compound **4a** protonation took place on the pyridine ring nitrogen atom, while it was preferable by 15.1 kcal/mol in comparison to the α-amino-group protonation and by 14.2 kcal/mol to the γ-amino-group.

The calculation results confirmed that the protonation took place first on the pyridine ring, so we have not carried out such calculations for the other compounds in order to reduce computation time, and optimized their geometry assuming protonation of the first pyridine position.

The calculation for the protonated molecules shows the great changing of electron density distribution in them. As the nitrogen atom in chromeno[2,3-*b*]pyridine cycle became positively charged and revealed high electron-withdrawing effect, the electron density in the pyridine ring decreased. This process was also strengthened by the influence of the cyano-group in this ring. In all nine compounds the LUMOs were localized on the atoms of pyridine ring, while the HOMOs were localized either on the benzene ring atoms (compounds **4a**, **4b**, **4d**, **4i**, Table 4) or on the carbonyl group of the hydroxyquinolinone ring (compounds **4c**, **4e**, **4f**, **4g**, **4h**, Table 4).

The key parameters remained low for compounds **4a**–**i** even in the protonated state. Global electrophilicity ω was extremely low (<60 is acceptable, [53]), the softness σ and electronegativity χ were almost twice as low as the values for known cationic dies (<1 and <10 are acceptable, respectively [53]).

The analysis of the key parameters for the protonated molecules of **4a**–**i** shows that the highest anticorrosion effect was possessed by the compounds **4b**, **4c** and **4d**, the lowest by **4f** and **4h**. However, despite of the presence of an electron-withdrawing substituent and the high value of global electrophilicity ω, compound **4h** had the highest value of chemical softness σ, which indicates that this compound can readily react with forming Fe^2+^ ions (soft acid with soft base).

## 3. Materials and Methods

### 3.1. Synthesis

#### 3.1.1. General Information

All melting points were measured with a Gallenkamp melting point apparatus (London, UK). ^1^H and ^13^C NMR spectra were recorded with Bruker AM−300 spectrometer (Billerica, MA, USA) at ambient temperature. Chemical shifts values were relative to Me_4_Si. IR spectra were registered with a Bruker ALPHA-T FT-IR spectrometer (Billerica, MA, USA) in KBr pellets. Mass spectra (EI, 70 eV) were obtained directly with a Finnigan MAT INCOS 50 spectrometer (Bremen, Germany). High-resolution mass spectra (HRMS) were measured on a Bruker micrOTOF II instrument (Billerica, MA, USA) using electrospray ionization (ESI).

#### 3.1.2. Synthesis of **4a**–**i**

Salicylaldehyde **1a**–**i** (1 mmol), 2-aminoprop-1-ene-1,1,3-tricarbonitrile **2** (0.13 g, 1 mmol) and 4-hydroxyquinolin-2(1*H*)-one **3** (0.16 g, 1 mmol) were refluxed in 4 mL of ethanol–pyridine (3:1) mixture for 2 h. After the reaction was completed, the solid was filtered, washed with well-chilled methanol (3 × 2 mL) and dried to isolate pure substituted 2,4-diamino-5-(4-hydroxy-2-oxo-1,2-dihydroquinolin-3-yl)-5*H*-chromeno[2,3-*b*]pyridine-3-carbonitriles **4a**–**i**.

2,4-Diamino-5-(4-hydroxy-2-oxo-1,2-dihydroquinolin-3-yl)-5*H*-chromeno[2,3-*b*]pyri-dine-3-carbonitrile **4a**, (white solid, 0.377g, 95%), mp > 350 °C (from Py-EtOH), FTIR (KBr) cm^−1^: 3391, 3184, 2993, 2846, 2201, 1642, 1606, 1399, 1237, 751. ^1^H-NMR (400 MHz, DMSO-*d6*) δ 5.58 (s, 1H, CH), 6.31 (s, 2H, NH_2_), 6.44 (s, 2H, NH_2_), 6.90–7.05 (m, 3H, Ar), 7.06–7.22 (m, 2H, Ar), 7.28–7.41 (m, 2H, Ar), 7.86 (d, *J* = 7.8 Hz, 1H, Ar), 10.77 (br s, 1H, OH), 11.86 (s, 1H, NH) ppm. ^13^C-NMR (100 MHz, DMSO-d6) δ 28.68, 70.32, 88.97, 115.24, 115.41 (2C), 115.61, 116.54, 121.60, 122.16, 123.39, 127.42 (2C), 128.68, 130.76, 137.49, 151.27, 156.72, 159.19, 159.35, 160.19, 164.18. MS (EI, 70 eV) *m*/*z* (%): 397 (M^+^, 17), 376 (11), 304 (7), 252 (6), 237 (100), 171 (12), 161 (26), 119 (12), 79 (86), 52 (55). HRMS-ESI: [M + H]^+^, calcd for C_22_H_16_N_5_O_3_ 398.1253, found 398.1252.

2,4-Diamino-5-(4-hydroxy-2-oxo-1,2-dihydroquinolin-3-yl)-7-methyl-5*H*-chromeno[2,3-*b*]pyridine-3-carbonitrile **4b**, (white solid, 0.358g, 87%), mp > 350 °C (from Py-EtOH), FTIR (KBr) cm^−1^: 3419, 3361, 2881, 2845, 2203, 1634, 1580, 1402, 1220, 752. ^1^H-NMR (400 MHz, DMSO-*d6*) δ 2.14 (s, 3H, CH_3_), 5.53 (s, 1H, CH), 6.28 (s, 2H, NH_2_), 6.41 (s, 2H, NH_2_), 6.78 (s, 1H, Ar), 6.93 (dd, ^3^*J* = 16.6 Hz, ^4^*J* = 8.0 Hz, 2H, Ar), 7.11 (t, *J* = 7.4Hz, 1H, Ar), 7.33 (d, *J* = 7.8 Hz, 1H, Ar), 7.48 (t, *J* = 7.3 Hz, 1H, Ar), 7.86 (d, *J* = 7,8 Hz, 1H, Ar), 10.74 (s, 1H, OH), 11.84 (s, 1H, NH) ppm. ^13^C-NMR (100 MHz, DMSO-d6) δ 20.11, 28.64, 70.20, 89.00, 115.18 (2C), 115.61, 116.58, 121.48, 122.14, 123.15, 127.97 (2C), 128.34, 130.73, 132.22, 137.46, 149.20, 156.68, 159.15,159.49 160.09, 164.18. MS (EI, 70 eV) *m*/*z* (%): 411 (M^+^, 26), 390 (7), 251 (100), 223 (14), 185 (28), 161 (61), 119 (43), 92 (30), 77 (35), 42 (15). HRMS-ESI**:** [M + H]^+^, calcd for C_23_H_18_N_5_O_3_ 412.1410, found 412.1402.

2,4-Diamino-5-(4-hydroxy-2-oxo-1,2-dihydroquinolin-3-yl)-8-methoxy-5*H*-chromeno[2,3-*b*]pyridine-3-carbonitrile **4c**, (white solid, 0.355g, 83%), mp > 350°C (from Py-EtOH), FTIR (KBr) cm^−1^: 3428, 3381, 2887, 2836, 2199, 1632, 1607, 1569, 1402, 1201, 761. ^1^H-NMR (400 MHz, DMSO-*d6*) δ 3.72 (s, 3H, OMe), 5.50 (s, 1H, CH), 6.29 (s, 2H, NH_2_), 6.43 (s, 2H, NH_2_), 6.51–6.65 (m, 2H, Ar), 6.88 (d, *J* = 7.9 Hz, 1H, Ar), 7.11 (t, *J* = 7.2 Hz, 1H, Ar), 7.33 (d, *J* = 7.9 Hz, 1H, Ar), 7.48 (t, *J* = 7.2 Hz, 1H, Ar), 7.86 (d, *J* = 7.3 Hz, 1H, Ar), 10.71 (br s, 1H, OH), 11.83 (s, 1H, NH) ppm. ^13^C-NMR (100 MHz, DMSO-d6) δ 28.08, 30.63, 55.21, 70.29, 89.12, 100.60 (2C), 109.69, 115.27, 115.55 (2C), 116.50, 121.43, 122.11, 128.75, 130.64, 137.40, 151.93, 156.68, 158.53, 159.10, 159.97, 164.13. MS (EI, 70 eV) *m*/*z* (%): 407 (18), 267 (100), 252 (11), 237 (9), 224 (17), 201 (4), 161 (60), 119 (43), 92 (33), 15 (63). HRMS-ESI**:** [M + H]^+^, calcd for C_23_H_18_N_5_O_4_ 428.1359, found 428.1351.

2,4-Diamino-9-ethoxy-5-(4-hydroxy-2-oxo-1,2-dihydroquinolin-3-yl)-5*H*-chromeno[2,3-*b*]pyridine-3-carbonitrile **4d**, (white solid, 0.433 g, 98%), mp > 350 °C (from Py-EtOH), FTIR (KBr) cm^−1^: 3409, 3372, 2979, 2881, 2202, 1631, 1568, 1483, 1222, 751. ^1^H-NMR (400 MHz, DMSO-*d6*) δ 1.39 (t, *J* = 6.8 Hz, 3H, OEt), 4.04 (m, 2H, OEt), 5.56 (s, 1H, CH), 6.35 (s, 2H, NH_2_), 6.41 (s, 2H, NH_2_), 6.52 (d, *J* = 6.4 Hz, 1H, Ar), 6.78–6.90 (m, 2H, Ar), 7.10 (t, *J* = 7.6 Hz, 1H, Ar), 7.33 (d, *J* = 8.1 Hz, 1H, Ar), 7.48 (t, *J* = 7.6 Hz, 1H, Ar), 7.88 (d, *J* = 8.1 Hz, 1H, Ar), 10.80 (s, 1H, OH), 11.83 (s, 1H, NH) ppm. ^13^C-NMR (100 MHz, DMSO-d6) δ 14.81, 28.75, 63.62, 70.22, 88.77, 110.79, 115.22, 115.57, 116.55, 119.49 (2C), 121.47, 122.10, 122.90, 124.00, 130.71, 137.43, 140.78, 145.90, 156.64, 159.18 (2C), 160.21, 164.12. MS (EI, 70 eV) *m*/*z* (%): 441 (M^+^, 19), 412 (18), 392 (26), 281 (74), 253 (77), 187 (14), 161 (100), 119 (57), 92 (42), 29 (95). HRMS-ESI: [M + H]^+^, calcd for C_24_H_19_N_5_O_4_ 442.1510, found 442.1503.

2,4-Diamino-7-bromo-5-(4-hydroxy-2-oxo-1,2-dihydroquinolin-3-yl)-9-methoxy-5*H*-chromeno[2,3-*b*]pyridine-3-carbonitrile **4e**, (white solid, 0.344 g, 68%), mp > 350 °C (from Py-EtOH), FTIR (KBr) cm^−1^: 3445, 3387, 2204, 1639, 1600, 1567, 1398, 1224, 1013, 768. ^1^H-NMR (400 MHz, DMSO-*d6*) δ 3.84 (s, 3H, OMe), 5.53 (s, 1H, CH), 6.33 (s, 2H, NH_2_), 6.42 (br s, 2H, NH_2_), 6.64 (s, 1H, Ar), 7.04 (s, 1H, Ar), 7.12 (t, *J* = 7.3 Hz, 1H, Ar), 7.34 (d, *J* = 8.1 Hz, 1H, Ar), 7.50 (t, *J* = 7.3 Hz, 1H, Ar), 7.88 (d, *J* = 7.2 Hz, 1H, Ar), 10.90 (s, 1H, OH), 11.86 (s, 1H, NH) ppm. ^13^C-NMR (100 MHz, DMSO-d6) δ 28.64, 56.06, 70.39, 88.34, 113.03, 113.23, 114.08, 115.07, 115.62, 116.37, 121.62, 122.20, 123.27, 125.73, 130.88, 131.78, 137.51, 137.71, 147.67, 156.58, 156.74, 159.13, 163.94. MS (EI, 70 eV) *m*/*z* (%): 507 (M^+^, 3), 346 (51), 331 (8), 305 (51), 252 (14), 204 (45), 161 (100), 119 (59), 92 (39), 15 (28). HRMS-ESI: [M + H]^+^, calcd for C_23_H_17_BrN_5_O_4_ 506.0464 [^79^Br], 508.0443 [^81^Br], found 506.0458 [^79^Br], 508.0434 [^81^Br].

2,4-Diamino-7-chloro-5-(4-hydroxy-2-oxo-1,2-dihydroquinolin-3-yl)-5*H*-chrome-no[2,3-*b*]pyridine-3-carbonitrile **4f**, (white solid, 0.393 g, 91%), mp > 350°C (from Py-EtOH), FTIR (KBr) cm^−1^: 3393, 3217, 2975, 2894, 2203, 1633, 1607, 1404, 1259, 757. ^1^H-NMR (400 MHz, DMSO-*d6*) δ 5.56 (s, 1H, CH), 6.35 (s, 2H, NH_2_), 6.47 (br s, 2H, NH_2_), 6.96 (s, 1H, Ar), 7.06 (d, *J* = 8.7 Hz, 1H, Ar), 7.13 (t, *J* = 7.6 Hz, 1H, Ar), 7.22 (d, *J* = 8.6 Hz, 1H, Ar), 7.34 (d, *J* = 7.9 Hz, 1H, Ar), 7.50 (t, *J* = 7.6 Hz, 1H, Ar), 7.88 (d, *J* = 6.0 Hz, 1H, Ar), 10.89 (br s, 1H, OH), 11.88 (s, 1H, NH) ppm. ^13^C-NMR (100 MHz, DMSO-d6) δ 28.73, 70.43, 88.31, 115.15, 115.37, 115.66, 116.39, 117.27 (2C), 121.51, 122.27, 125.62, 126.68, 127.36, 127.48, 130.90, 137.60, 150.23, 156.65, 156.80, 159.20, 163.98. MS (EI, 70 eV) *m*/*z* (%): 431 (M^+^, 17), 409 (9), 271 (100), 243 (10), 205 (21), 161 (79), 119 (73), 92 (92), 77 (54), 28 (52). HRMS-ESI: [M + H]^+^, calcd for C_22_H_15_ClN_5_O_3_ 432.0863 [^35^Cl], 434.0834 [^37^Cl], found 432.0856 [^35^Cl], 434.0824 [^37^Cl].

2,4-Diamino-7-bromo-5-(4-hydroxy-2-oxo-1,2-dihydroquinolin-3-yl)-5H-chrome-no[2,3-b]pyridine-3-carbonitrile **4g**, (white solid, 0.324 g, 68%), mp > 231–233 °C (from Py-EtOH), FTIR (KBr) cm^−1^: 3343, 3184, 2993, 2846, 2201, 1642, 1609, 1398, 1258, 775. ^1^H-NMR (400 MHz, DMSO-*d6*) δ 5.56 (s, 1H, CH), 6.35 (s, 2H, NH_2_), 6.46 (br s, 2H, NH_2_), 7.01 (d, *J* = 8.5 Hz, 1H, Ar), 7.08 (s, 1H, Ar), 7.13 (t, *J* = 7.5 Hz, 1H, Ar), 7.35 (d, *J* = 7.9 Hz, 2H Ar), 7.50 (t, *J* = 7.5 Hz, 1H, Ar), 7.88 (d, *J* = 6.0 Hz, 1H Ar), 10.89 (s, 1H, OH), 11.87 (s, 1H, NH) ppm. ^13^C-NMR (100 MHz, DMSO-d6) δ 28.61, 70.43, 88.34, 114.47, 115.07, 115.65 (2C), 116.36, 117.72, 121.52, 122.27, 126.09, 130.23, 130.34, 130.90, 137.56, 150.67, 156.67, 158.92, 159.20, 160.32, 163.90. MS (EI, 70 eV) *m*/*z* (%): 316 ([M-C_9_H_6_NO_2_]^+^, ^81^Br, 2), 314 ([M-C_9_H_6_NO_2_]^+^, ^79^Br, 2), 236 (2), 202 (1), 171 (1), 161 (13), 119 (9), 92 (5), 78 (73), 63 (100), 15 (31). HRMS-ESI**:** [M + H]^+^, calcd for C_22_H_14_BrN5O_3_ 476.0353 [^79^Br], 478.0333 [^81^Br], found 476.0324 [^79^Br], 478.0323 [^81^Br].

2,4-Diamino-5-(4-hydroxy-2-oxo-1,2-dihydroquinolin-3-yl)-7-nitro-5*H*-chrome-no[2,3-*b*]pyridine-3-carbonitrile **4h**, (yellow solid, 0.261 g, 59%), mp > 350 °C (from Py-EtOH), FTIR (KBr) cm^−1^: 3357, 3184, 2199, 1661, 1627, 1336, 1242, 1026, 828, 752. ^1^H-NMR (400 MHz, DMSO-*d6*) δ 5.24 (s, 1H, CH), 6.53 (br s, 2H, NH_2_), 6.56 (s, 2H, NH_2_), 7.28 (t, *J* = 7.6 Hz, 1H, Ar), 7.34 (d, *J* = 8.3 Hz, 1H Ar), 7.42 (d, *J* = 8.3 Hz, 1H Ar), 7.54 (t, *J* = 7.6 Hz, 1H, Ar), 7.87–7.94 (m, 1H, Ar), 8.02 (d, *J* = 8.3 Hz, 1H Ar), 8.08 (dd, ^3^*J* = 7.0 Hz, ^4^*J* = 2.6 Hz, 1H, Ar), 9.57 (s, 1H, OH), 11.62 (s, 1H, NH) ppm. ^13^C-NMR (100 MHz, DMSO-d6) δ 28.45, 98.42, 107.18, 112.75, 115.20 (2C), 116.43, 116.77, 121.76 (2C), 123.02, 124.14, 126.22, 130.52, 137.22, 143.17, 152.75, 153.74, 154.75, 155.08, 159.76, 161.57. MS (EI, 70 eV) *m*/*z* (%): 294 (11), 247 (10), 236 (3), 190 (3), 161 (20), 150 (100), 122 (65), 78 (16), 63 (31), 18 (32). HRMS-ESI: [M + H]^+^, calcd for C_22_H_15_N_6_O_5_ 443.1104, found 443.1098.

9,11-Diamino-12-(4-hydroxy-2-oxo-1,2-dihydroquinolin-3-yl)-12*H*-benzo[5,6]chromeno[2,3-*b*]pyridine-10-carbonitrile **4i**, (white solid, 0.389 g, 87%), mp > 350 °C (from Py-EtOH), FTIR (KBr) cm^−1^: 3454, 3400, 2875, 2835, 2203, 1634, 1607, 1409, 1239, 755. ^1^H-NMR (400 MHz, DMSO-*d6*) δ 6.06 (s, 1H, CH), 6.35 (s, 2H, NH_2_), 6.69 (s, 2H, NH_2_), 7.07 (t, *J* = 7.6 Hz, 1H, Ar), 7.25–7.51 (m, 5H, Ar), 7.76–7.93 (m, 3H, Ar), 8.02 (d, *J* = 8.4 Hz, 1H Ar), 10.82 (s, 1H, OH), 11.95 (s, 1H, NH) ppm. ^13^C-NMR (100 MHz, DMSO-d6) δ 26.76, 70.47, 89.08, 114.46, 115.21, 115.58, 116.51, 117.06 (2C), 121.56, 122.13, 122.68, 124.08, 126.83, 128.30, 128.44 (2C), 130.18, 130.78, 131.32, 137.26, 149.24, 156.86, 159.17, 160.96, 163.90. MS (EI, 70 eV) *m*/*z* (%): 429 ([M-H_2_O]^+^, 5), 410 (14), 364 (19), 287 (100), 258 (6), 221 (13), 161 (35), 144 (38), 92 (12), 63 (13). HRMS-ESI**:** [M + H]^+^, calcd for C_26_H_18_N_5_O_3_ 448.1410, found 448.1401.

#### 3.1.3. Isolation of the Intermediate **5**

Salicylaldehyde **1a** (0.12 g, 1 mmol) and 2-aminoprop-1-ene-1,1,3-tricarbonitrile **2** (0.13 g, 1 mmol) were stirred at room temperature in 4 mL of ethanol–pyridine (3:1) mixture for 2 h. After the reaction was completed, the solid was filtered, washed with well-chilled methanol (3 × 2 mL) and dried to isolate pure 2-(amino-(2-imino-2*H*-chromen-3-yl)methylene)malononitrile **5**.

2-(Amino-(2-imino-2*H*-chromen-3-yl)methylene)malononitrile **5**, (Yellow solid, 0.208 g, yield 88%), m.p. 272–273 °C (decomp.) (lit [40] m.p. 271–272 °C (decomp.)). ^1^H-NMR (300 MHz, DMSO-d6) δ 7.11–7.31 (m, 2H, 2CH Ar), 7.45–7.63 (m, 2H, 2CH Ar), 7.75 (s, 1H, CH), 8.65 (s, 1H, NH), 8.93 (br.s, 1H, N*H*H), 8.95 (br.s, 1H, NH*H*) ppm.

#### 3.1.4. Synthesis of Chromeno[2,3-*b*]pyridine **4a** from 2-(amino-(2-imino-2*H*-chromen-3-yl)methylene)malononitrile **5** and 4-hydroxyquinolin-2(1*H*)-one **3**

2-(Amino-(2-imino-2*H*-chromen-3-yl)methylene)malononitrile **5** (0.24 g, 1 mmol) and 4-hydroxyquinolin-2(1*H*)-one (0.16 g, 1 mmol) **3** were refluxed in 4 mL of ethanol–pyridine (3:1) mixture for 2 h. After the reaction was completed, the solid was filtered, washed with well-chilled methanol (3 × 2 mL) and dried to isolate pure chromeno[2,3-*b*]pyridine **4a**.

2,4-Diamino-5-(4-hydroxy-2-oxo-1,2-dihydroquinolin-3-yl)-5*H*-chromeno[2,3-*b*]pyridine-3-car-bonitrile **3a**, (White solid, 0.377g, 95%), mp > 350°C (from Py-EtOH), ^1^H-NMR (400 MHz, DMSO-*d6*) δ 5.58 (s, 1H, CH), 6.31 (s, 2H, NH_2_), 6.44 (s, 2H, NH_2_), 6.90–7.05 (m, 3H, Ar), 7.06–7.22 (m, 2H, Ar), 7.28–7.41 (m, 2H, Ar), 7.86 (d, *J* = 7.8 Hz, 1H, Ar), 10.77 (br s, 1H, OH), 11.86 (s, 1H, NH) ppm.

## 4. Conclusions

In summary, the PASE transformation of salicylaldehydes, malononitrile dimer and hydroxyquinolinone into previously unknown hydroxyquinolinone substituted chromeno[2,3-*b*]pyridines has been found. The proposed EtOH-Py catalyst-solvent system provides an efficient rout to hydroxyquinolinone substituted chromeno[2,3-*b*]pyridines.

The developed approach is facile, it is easy to isolate final compounds directly from reaction mixture and the yields of final compounds are 59%–98%.

During investigation of reaction mechanism using a real-time ^1^H NMR monitoring, 2-((2*H*-chromen-3-yl)methylene)malononitrile was determined and proven as the key intermediate in assembling of chromeno[2,3-*b*]pyridines.

Quantum chemistry calculations showed that hydroxyquinolinone-substituted chromeno[2,3-*b*]pyridines bearing electron-donating groups and hydroxynaphthaldehyde derivatives are prospective compounds for corrosion inhibition. In particular, methyl, methoxy, ethoxy substituted 5-(1,2-dihydroquinolin-3-yl)-5*H*-chromeno[2,3-*b*]pyridines are the most effective among those examined according to calculation.

## Figures and Tables

**Table 1:** Optimization of reaction conditions ^1^.

Entry	Solvent	Catalyst	Time (h)	Temp (°C)	Yield (%)
1	Solvent-free	-	2	80	17
2	Solvent-free	KF	2	80	25
3	Solvent-free	AcONa	2	80	37
4	H_2_O	-	2	80	16
5	H_2_O	AcONa	2	80	31
6	EtOH	-	2	78	19 ^2^
7	EtOH	AcONa	2	78	35 ^2^
8	EtOH	Et_3_N	2	78	63 ^2^
9	EtOH	Morph	2	78	57 ^2^
10	Py	-	2	116	61 ^2^
11	EtOH-Py (4:1)	-	2	78	87 ^2^
12	EtOH-Py (3:1)	-	2	78	95 ^2^
13	EtOH-Py (2:1)	-	2	78	92 ^2^
14	EtOH-Py (3:1)	-	1	78	89 ^2^

^1^ Reaction conditions **1a** (1 mmol), **2** (1 mmol), **3** (1 mmol) were heated in 4 mL of solvent or without solvent; with 10 mol% of catalyst or without catalyst. ^2^ Isolated yield, in other cases NMR data.

**Table 2:** PASE reaction of salicylaldehydes **1a–i**, malononitrile dimer **2** and hydroxyquinolinone **3** ^1^.

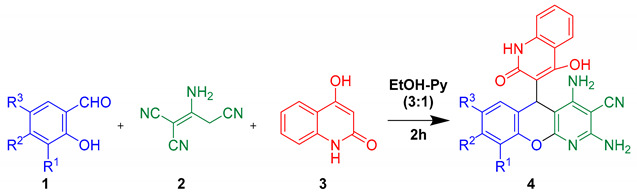
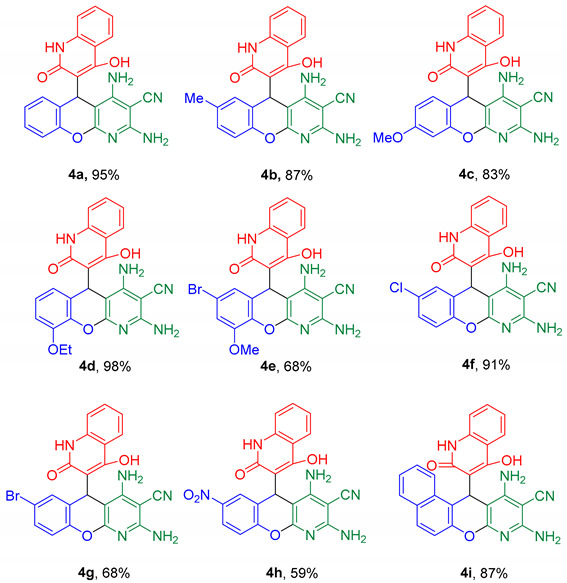

^1^ Reaction conditions: **1a**–**i** (1 mmol), **2** (1 mmol), **3** (1 mmol) were refluxed in 4 mL of EtOH-Py mixture (3:1). Isolated yields.

**Table 3:** Results of quantum chemical calculations for studied compounds **4a**–**i**.

Compound	4a	4b	4c	4d	4e	4f	4g	4h	4i
**Gas Phase Calculations**									
Total energy, a.u.	−1347.19	−1386.52	−1461.75	−1501.07	−4035.28	−1806.81	−3920.74	−1551.75	−1500.87
*E_(HOMO)_, eV*	−5.707	−5.649	−5.644	−5.558	−5.695	−5.835	−5.830	−6.065	−5.669
*E_(LUMO),_ eV*	−2.040	−2.023	−1.997	−1.982	−2.072	−2.135	−2.131	−2.416	−2.047
*ΔE_(L-H),_ eV*	3.667	3.626	3.647	3.576	3.623	3.700	3.699	3.649	3.622
*μ, D*	7.896	8.071	7.645	8.119	7.487	7.325	7.351	7.619	7.670
*χ*	3.874	3.836	3.821	3.770	3.884	3.986	3.981	4.241	3.858
*η*	1.834	1.813	1.824	1.788	1.812	1.85	1.849	1.825	1.811
*ω*	4.092	4.058	4.003	3.975	4.164	4.301	4.286	4.929	4.109
*σ*	0.545	0.552	0.548	0.559	0.552	0.541	0.541	0.548	0.552
**Calculations for Solvated Compounds**									
Total energy, a.u.	−1347.22	−1386.55	−1461.77	−1501.10	−4035.31	−1806.84	−3920.76	−1551.78	−1500.90
*E_(HOMO)_, eV*	−5.993	−5.947	−5.940	−5.900	−5.968	−6.037	−6.035	−6.139	−5.934
*E_(LUMO),_ eV*	−1.795	−1.791	−1.783	−1.791	−1.822	−1.826	−1.825	−2.726	−1.810
*ΔE_(L-H),_ eV*	4.198	4.156	4.157	4.109	4.146	4.211	4.210	3.413	4.124
*μ, D*	10.263	11.789	9.883	10.873	11.137	9.490	9.486	9.609	10.135
*χ*	3.894	3.869	3.867	3.846	3.895	3.932	3.930	4.433	3.872
*η*	2.099	2.078	2.079	2.055	2.073	2.106	2.105	1.707	2.062
*ω*	3.612	3.602	3.596	3.599	3.659	3.671	3.669	5.756	3.635
*σ*	0.476	0.481	0.481	0.487	0.482	0.475	0.475	0.586	0.485
*Calculations for protonated forms of studied compounds*									
Total energy, a.u.	−1347.67	−1386.99	−1462.22	−1501.55	−4035.76	−1807.29	−3921.21	−1552.22	−1501.34
*E_(HOMO)_, eV*	−6.592	−6.541	−6.374	−6.350	−6.449	−6.623	−6.617	−6.670	−6.321
*E_(LUMO),_ eV*	−1.983	−1.971	−1.967	−1.977	−2.026	−2.035	−2.033	−2.927	−2.044
*ΔE_(L-H),_ eV*	4.609	4.57	4.407	4.373	4.423	4.588	4.584	3.743	4.277
*μ, D*	9.704	9.785	11.099	8.722	13.356	12.543	13.784	16.319	11.489
*χ*	4.288	4.256	4.171	4.164	4.263	4.329	4.325	4.799	4.183
*η*	2.305	2.285	2.204	2.187	2.212	2.294	2.292	1.872	2.139
*ω*	3.988	3.964	3.947	3.964	4.108	4.085	4.081	6.151	4.090
*σ*	0.434	0.438	0.454	0.457	0.452	0.436	0.436	0.534	0.468

Ionization potential [53]: I ≈ −E_(HOMO)_; electron affinity as: A ≈ −E_(LUMO)_; electronegativity: χ ≈ (I + A)/2; chemical hardness: η ≈ (I − A)/2; global electrophilicity index: ω = (χ^2^)/2η; global chemical softness: σ = 1/η. this table with the extended decimal point is presented in Supplementary Materials (Table S1).

**Table 4:** Frontier orbitals of several studied compounds.

Compound	HOMO	LUMO
**In Gas Phase**		
**4a**	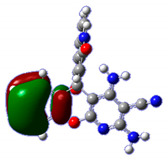	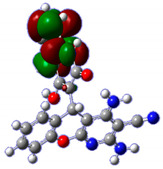
**4e**	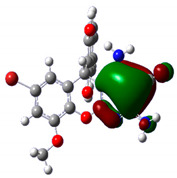	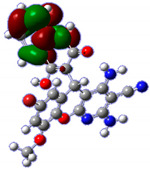
**4h**	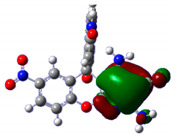	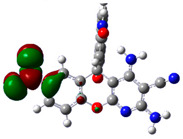
**Solvated Molecules**		
**4c**	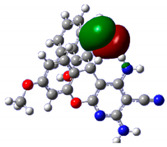	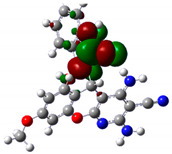
**Protonated Molecules**		
**4a**	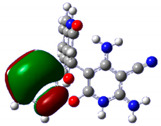	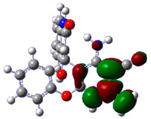
**4h**	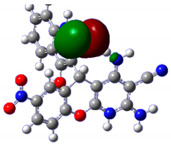	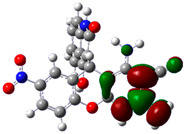

Frontier orbitals of these compounds are also presented in Figures S43–S55 of Supplementary Materials.

## Supplementary Materials

^1^H and ^13^C Spectra of synthesized compounds **4a**–**i** and intermediate **5** (Figure S1–S22)**,** NMR monitoring spectra (Figure S23–S42), results of quantum chemical calculations with extended decimal point (Table S1) and figures of frontier orbitals (Figure S44–S55) are available online.
