# Supplementary material for: Catalyst-Solvent System for PASE Approach to Hydroxyquinolinone-Substituted Chromeno[2,3-b]pyridines Its Quantum Chemical Study and Investigation of Reaction Mechanism
Source: Molecules. 2020 May 31;25(11):2573. doi: 10.3390/molecules25112573 (PMC7321101; doi:10.3390/molecules25112573)
Supplement: Supplementary file 1 [file molecules-25-02573-s001.pdf]

Catalyst-solvent system for PASE approach to hydroxyquinolinone-substituted chromeno[2,3-b]pyridines, its quantum chemical study and investigation of reaction mechanism

Fedor V. Ryzhkov,[a] Yuliya E. Ryzhkova,[a] Michail N. Elinson,[*a] Stepan V. Vorobyev,[b] Artem N. Fakhrutdinov,[a] Anatoly N. Vereshchagin,[a] Mikhail P. Egorov[a]

[a] N. D. Zelinsky Institute of Organic Chemistry Russian academy of sciences

Leninsky pr. 47, Moscow, 119991, Russian Federation E-mail: elinson@ioc.ac.ru

[b] Gubkin Russian State University of Oil and Gas, 65

Leninsky prospect, Moscow 119991, Russian Federation.

Supporting Information

Table of Contents

Contents

General information....................2

^{1}H and ^{13}C Spectra of Compounds....................7

Compound **4a**....................7

Compound **4b**....................9

Compound **4c**....................11

Compound **4d**....................13

Compound **4e**....................15

Compound **4f**....................17

Compound **4g**....................19

Compound **4h**....................21

Compound **4i**....................23

^{1}H NMR monitoring....................25

Quantum chemistry simulations....................51

Frontier orbitals of several studied compounds....................53

References....................59

General information

All melting points were measured with a Gallenkamp melting point apparatus. ^{1}H and ^{13}C NMR spectra were recorded with Bruker AM-300 spectrometer at ambient temperature. Chemical shifts values are relative to Me_4Si. IR spectra were registered with a Bruker ALPHA-T FT-IR spectrometer in KBr pellets. Mass spectra (EI, 70 eV) were obtained directly with a Finningan MAT INCOS 50 spectrometer. High-resolution mass spectra (HRMS) were measured on a Bruker micrOTOF II instrument using electrospray ionization (ESI).

Salicylaldehyde **1a-i** (1 mmol), 2-aminoprop-1-ene-1,1,3-tricarbonitrile **2** (0.13 g, 1 mmol) and 4-hydroxyquinolin-2(1*H*)-one (1 mmol) **3** were reluxed in 4 ml of ethanol–pyridine (3:1) mixture for 2 h. After the reaction was completed, the solid was filtered, washed with well-chilled methanol (3 × 2 mL) and dried to isolate pure substituted 2,4-diamino-5-(4-hydroxy-2-oxo-1,2-dihydroquinolin-3-yl)-5*H*-chromeno[2,3-*b*]pyridine-3-carbonitriles **4a–i**.

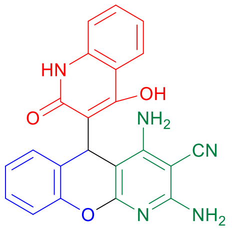

4a, 95%

2,4-Diamino-5-(4-hydroxy-2-oxo-1,2-dihydroquinolin-3-yl)-5*H*-chromeno[2,3-*b*]pyridine-3-carbonitrile, (White solid, 0.377g, 95%), mp > 350°C (from Py-EtOH), **FTIR (KBr) cm^{-1}:** 3391, 3184, 2993, 2846, 2201, 1642, 1606, 1399, 1237, 751. **^{1}H-NMR** (400 MHz, DMSO-*d6*) δ 5.58 (s, 1H, CH), 6.31 (s, 2H, NH_2), 6.44 (s, 2H, NH_2), 6.90-7.05 (m, 3H, Ar), 7.06-7.22 (m, 2H, Ar), 7.28-7.41 (m, 2H, Ar), 7.86 (d, *J* = 7.8 Hz, 1H, Ar), 10.77 (br s, 1H, OH), 11.86 (s, 1H, NH) ppm. **^{13}C-NMR** (100 MHz, DMSO-d6) δ 28.68, 70.32, 88.97, 115.24, 115.41 (2C), 115.61, 116.54, 121.60, 122.16, 123.39, 127.42 (2C), 128.68, 130.76, 137.49, 151.27, 156.72, 159.19, 159.35, 160.19, 164.18. **MS (EI, 70 eV) m/z (%):** 397 (M^+, 17), 376 (11), 304 (7), 252 (6), 237 (100), 171 (12), 161 (26), 119 (12), 79 (86), 52 (55). **HRMS-ESI:** $[M+H]^+$, calcd for $C_{22}H_{16}N_5O_3$ 398.1253, found 398.1252.

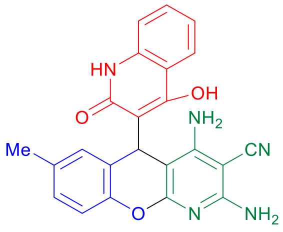

4b, 87%

2,4-Diamino-5-(4-hydroxy-2-oxo-1,2-dihydroquinolin-3-yl)-7-methyl-5*H*-chromeno[2,3-*b*]pyridine-3-carbonitrile, (White solid, 0.358g, 87%), mp > 350°C (from Py-EtOH), **FTIR (KBr) cm^{-1}:** 3419, 3361, 2881, 2845, 2203, 1634, 1580, 1402, 1220, 752. **^{1}H-NMR** (400 MHz, DMSO-*d6*) δ 2.14 (s, 3H, CH_3), 5.53 (s, 1H, CH), 6.28 (s, 2H, NH_2), 6.41 (s, 2H, NH_2), 6.78 (s, 1H, Ar), 6.93 (dd, ^{3}J = 16.6 Hz, ^{4}J = 8.0 Hz, 2H, Ar), 7.11 (t, *J* = 7.4Hz, 1H, Ar), 7.33 (d, *J* = 7.8 Hz, 1H, Ar), 7.48 (t, *J* = 7.3 Hz, 1H, Ar), 7.86 (d, *J* = 7,8 Hz, 1H, Ar), 10.74 (s, 1H, OH), 11.84 (s, 1H, NH) ppm. **^{13}C-NMR** (100 MHz, DMSO-d6) δ 20.11, 28.64, 70.20, 89.00, 115.18 (2C), 115.61, 116.58, 121.48, 122.14, 123.15, 127.97 (2C), 128.34, 130.73, 132.22, 137.46, 149.20, 156.68, 159.15,159.49 160.09, 164.18. **MS (EI, 70 eV) m/z (%):** 411 (M^+, 26), 390 (7), 251 (100), 223 (14), 185 (28), 161 (61), 119 (43), 92 (30), 77 (35), 42 (15). **HRMS-ESI:** $[M+H]^+$, calcd for $C_{23}H_{18}N_5O_3$ 412.1410, found 412.1402.

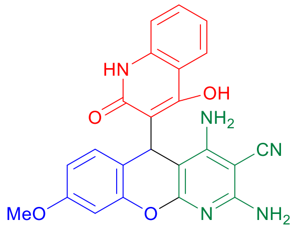

4c, 83% 2,4-diamino-5-(4-hydroxy-2-oxo-1,2-dihydroquinolin-3-yl)-8-methoxy-5*H*-chromeno[2,3-*b*]pyridine-3-carbonitrile, (White solid, 0.355g, 83%), mp > 350°C (from Py-EtOH), **FTIR (KBr) cm^{-1}:** 3428, 3381, 2887, 2836, 2199, 1632, 1607, 1569, 1402, 1201, 761. **^{1}H-NMR** (400 MHz, DMSO-*d6*) δ 3.72 (s, 3H, OMe), 5.50 (s, 1H, CH), 6.29 (s, 2H, NH_2), 6.43 (s, 2H, NH_2), 6.51-6.65 (m, 2H, Ar), 6.88 (d, *J* = 7.9 Hz, 1H, Ar), 7.11 (t, *J* = 7.2 Hz, 1H, Ar), 7.33 (d, *J* = 7.9 Hz, 1H, Ar), 7.48 (t, *J* = 7.2 Hz, 1H, Ar), 7.86 (d, *J* = 7.3 Hz, 1H, Ar), 10.71 (br s, 1H, OH), 11.83 (s, 1H, NH) ppm. **^{13}C-NMR** (100 MHz, DMSO-d6) δ 28.08, 30.63, 55.21, 70.29, 89.12, 100.60 (2C), 109.69, 115.27, 115.55 (2C), 116.50, 121.43, 122.11, 128.75, 130.64, 137.40, 151.93, 156.68, 158.53, 159.10, 159.97, 164.13. **MS (EI, 70 eV) m/z (%):** 407 (18), 267 (100), 252 (11), 237 (9), 224 (17), 201 (4), 161 (60), 119 (43), 92 (33), 15 (63). **HRMS-ESI:** $[M+H]^+$, calcd for $C_{23}H_{18}N_5O_4$ 428.1359, found 428.1351.

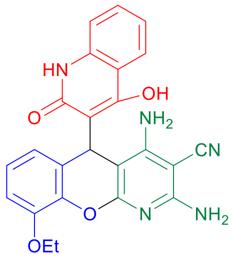

4d, 98% 2,4-diamino-9-ethoxy-5-(4-hydroxy-2-oxo-1,2-dihydroquinolin-3-yl)-5*H*-chromeno[2,3-*b*]pyridine-3-carbonitrile, (White solid, 0.433g, 98%), mp > 350°C (from Py-EtOH), **FTIR (KBr) cm^{-1}:** 3409, 3372, 2979, 2881, 2202, 1631, 1568, 1483, 1222, 751. **^{1}H-NMR** (400 MHz, DMSO-*d6*) δ 1.39 (t, *J* = 6.8 Hz, 3H, OEt), 4.04 (m, 2H, OEt), 5.56 (s, 1H, CH), 6.35 (s, 2H, NH_2), 6.41 (s, 2H, NH_2), 6.52 (d, *J* = 6.4 Hz, 1H, Ar), 6.78-6.90 (m, 2H, Ar), 7.10 (t, *J* = 7.6 Hz, 1H, Ar), 7.33 (d, *J* = 8.1 Hz, 1H, Ar), 7.48 (t, *J* = 7.6 Hz, 1H, Ar), 7.88 (d, *J* = 8.1 Hz, 1H, Ar), 10.80 (s, 1H, OH), 11.83 (s, 1H, NH) ppm. **^{13}C-NMR** (100 MHz, DMSO-d6) δ 14.81, 28.75, 63.62, 70.22, 88.77, 110.79, 115.22, 115.57, 116.55, 119.49 (2C), 121.47, 122.10, 122.90, 124.00, 130.71, 137.43, 140.78, 145.90, 156.64, 159.18 (2C), 160.21, 164.12. **MS (EI, 70 eV) m/z (%):** 441 (M^+, 19), 412 (18), 392 (26), 281 (74), 253 (77), 187 (14), 161 (100), 119 (57), 92 (42), 29 (95). **HRMS-ESI:** $[M+H]^+$, calcd for $C_{24}H_{19}N_5O_4$ 442.1510, found 442.1503.

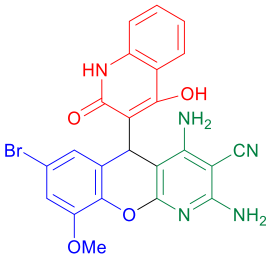

4e, 68%

2,4-Diamino-7-bromo-5-(4-hydroxy-2-oxo-1,2-dihydroquinolin-3-yl)-9-methoxy-5*H*-chromeno[2,3-*b*]pyridine-3-carbonitrile, (White solid, 0.344 g, 68%), mp > 350°C (from Py-EtOH), **FTIR (KBr) cm^{-1}:** 3445, 3387, 2204, 1639, 1600, 1567, 1398, 1224, 1013, 768. **^{1}H-NMR** (400 MHz, DMSO-*d6*) δ 3.84 (s, 3H, OMe), 5.53 (s, 1H, CH), 6.33 (s, 2H, NH_2), 6.42 (br s, 2H, NH_2), 6.64 (s, 1H, Ar), 7.04 (s, 1H, Ar), 7.12 (t, *J* = 7.3 Hz, 1H, Ar), 7.34 (d, *J* = 8.1 Hz, 1H, Ar), 7.50 (t, *J* = 7.3 Hz, 1H, Ar), 7.88 (d, *J* = 7.2 Hz, 1H, Ar), 10.90 (s, 1H, OH), 11.86 (s, 1H, NH) ppm. **^{13}C-NMR** (100 MHz, DMSO-d6) δ 28.64, 56.06, 70.39, 88.34, 113.03, 113.23, 114.08, 115.07, 115.62, 116.37, 121.62, 122.20, 123.27, 125.73, 130.88, 131.78, 137.51, 137.71, 147.67, 156.58, 156.74, 159.13, 163.94. **MS (EI, 70 eV) m/z (%):** 507 (M^+, 3), 346 (51), 331 (8), 305 (51), 252 (14), 204 (45), 161 (100), 119 (59), 92 (39), 15 (28). **HRMS-ESI:** $[M+H]^+$, calcd for $C_{23}H_{17}BrN_5O_4$ 506.0464 [^{79}Br], 508.0443 [^{81}Br], found 506.0458 [^{79}Br], 508.0434 [^{81}Br].

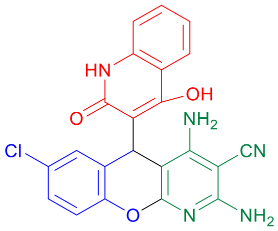

4f, 91%

2,4-Diamino-7-chloro-5-(4-hydroxy-2-oxo-1,2-dihydroquinolin-3-yl)-5*H*-chromeno[2,3-*b*]pyridine-3-carbonitrile, (White solid, 0.393 g, 91%), mp > 350°C (from Py-EtOH), **FTIR (KBr) cm^{-1}:** 3393, 3217, 2975, 2894, 2203, 1633, 1607, 1404, 1259, 757. **^{1}H-NMR** (400 MHz, DMSO-*d6*) δ 5.56 (s, 1H, CH), 6.35 (s, 2H, NH_2), 6.47 (br s, 2H, NH_2), 6.96 (s, 1H, Ar), 7.06 (d, *J* = 8.7 Hz, 1H, Ar), 7.13 (t, *J* = 7.6 Hz, 1H, Ar), 7.22 (d, *J* = 8.6 Hz, 1H, Ar), 7.34 (d, *J* = 7.9 Hz, 1H, Ar), 7.50 (t, *J* = 7.6 Hz, 1H, Ar), 7.88 (d, *J* = 6.0 Hz, 1H, Ar), 10.89 (br s, 1H, OH), 11.88 (s, 1H, NH) ppm. **^{13}C-NMR** (100 MHz, DMSO-d6) δ 28.73, 70.43, 88.31, 115.15, 115.37, 115.66, 116.39, 117.27 (2C), 121.51, 122.27, 125.62, 126.68, 127.36, 127.48, 130.90, 137.60, 150.23, 156.65, 156.80, 159.20, 163.98. **MS (EI, 70 eV) m/z (%):** 431 (M^+, 17), 409 (9), 271 (100), 243 (10), 205 (21), 161 (79), 119 (73), 92 (92), 77 (54), 28 (52). **HRMS-ESI:** $[M+H]^+$, calcd for $C_{22}H_{15}ClN_5O_3$ 432.0863 [^{35}Cl], 434.0834 [^{37}Cl], found 432.0856 [^{35}Cl], 434.0824 [^{37}Cl].

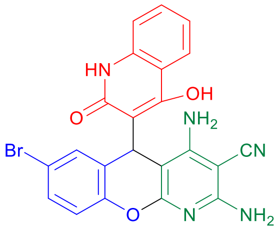

4g, 68%

2,4-Diamino-7-bromo-5-(4-hydroxy-2-oxo-1,2-dihydroquinolin-3-yl)-5H-chromeno[2,3-b]pyridine-3-carbonitrile, (White solid, 0.324 g, 68%), mp > 231-233 °C (from Py-EtOH), **FTIR (KBr) cm^{-1}:** 3343, 3184, 2993, 2846, 2201, 1642, 1609, 1398, 1258, 775. **^{1}H-NMR** (400 MHz, DMSO-*d6*) δ 5.56

(s, 1H, CH), 6.35 (s, 2H, NH_2), 6.46 (br s, 2H, NH_2), 7.01 (d, *J* = 8.5 Hz, 1H, Ar), 7.08 (s, 1H, Ar), 7.13 (t, *J* = 7.5 Hz, 1H, Ar), 7.35 (d, *J* = 7.9 Hz, 2H Ar), 7.50 (t, *J* = 7.5 Hz, 1H, Ar), 7.88 (d, *J* = 6.0 Hz, 1H Ar), 10.89 (s, 1H, OH), 11.87 (s, 1H, NH) ppm. **^{13}C-NMR** (100 MHz, DMSO-d6) δ 28.61, 70.43, 88.34, 114.47, 115.07, 115.65 (2C), 116.36, 117.72, 121.52, 122.27, 126.09, 130.23, 130.34, 130.90, 137.56, 150.67, 156.67, 158.92, 159.20, 160.32, 163.90. **MS (EI, 70 eV) m/z (%):** 316 ($[M-C_9H_6NO_2]^+$, ^{81}Br, 2), 314 ($[M-C_9H_6NO_2]^+$, ^{79}Br, 2), 236 (2), 202 (1), 171 (1), 161 (13), 119 (9), 92 (5), 78 (73), 63 (100), 15 (31). **HRMS-ESI:** $[M+H]^+$, calcd for $C_{22}H_{14}BrN5O_3$ 476.0353 [^{79}Br], 478.0333 [^{81}Br], found 476.0324 [^{79}Br], 478.0323 [^{81}Br].

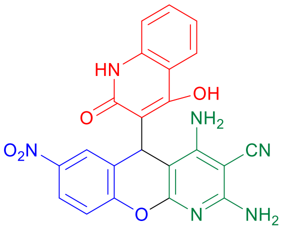

4h, 59%

2,4-Diamino-5-(4-hydroxy-2-oxo-1,2-dihydroquinolin-3-yl)-7-nitro-5*H*-chromeno[2,3-*b*]pyridine-3-carbonitrile, (Yellow solid, 0.261 g, 59%), mp > 350°C (from Py-EtOH), **FTIR (KBr) cm^{-1}:** 3357, 3184, 2199, 1661, 1627, 1336, 1242, 1026, 828, 752. **^{1}H-NMR** (400 MHz, DMSO-*d6*) δ 5.24 (s, 1H, CH), 6.53 (br s, 2H, NH_2), 6.56 (s, 2H, NH_2), 7.28 (t, *J* = 7.6 Hz, 1H, Ar), 7.34 (d, *J* = 8.3 Hz, 1H Ar), 7.42 (d, *J* = 8.3 Hz, 1H Ar), 7.54 (t, *J* = 7.6 Hz, 1H, Ar), 7.87-7.94 (m, 1H, Ar), 8.02 (d, *J* = 8.3 Hz, 1H Ar), 8.08 (dd, ^3J = 7.0 Hz, ^4J = 2.6 Hz, 1H, Ar), 9.57 (s, 1H, OH), 11.62 (s, 1H, NH) ppm. **^{13}C-NMR** (100 MHz, DMSO-d6) δ 28.45, 98.42, 107.18, 112.75, 115.20 (2C), 116.43, 116.77, 121.76 (2C), 123.02, 124.14, 126.22, 130.52, 137.22, 143.17, 152.75, 153.74, 154.75, 155.08, 159.76, 161.57. **MS (EI, 70 eV) m/z (%):** 294 (11), 247 (10), 236 (3), 190 (3), 161 (20), 150 (100), 122 (65), 78 (16), 63 (31), 18 (32). **HRMS-ESI:** $[M+H]^+$, calcd for $C_{22}H_{15}N_6O_5$ 443.1104, found 443.1098.

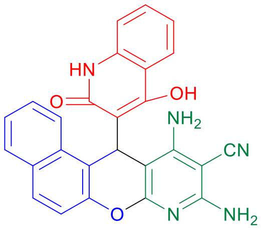

4i, 87%

9,11-diamino-12-(4-hydroxy-2-oxo-1,2-dihydroquinolin-3-yl)-12*H*-benzo[5,6]chromeno[2,3-*b*]pyridine-10-carbonitrile, (White solid, 0.389 g, 87%), mp > 350°C (from Py-EtOH), **FTIR (KBr) cm^{-1}:** 3454, 3400, 2875, 2835, 2203, 1634, 1607, 1409, 1239, 755. **^{1}H-NMR** (400 MHz, DMSO-*d6*) δ 6.06 (s, 1H, CH), 6.35 (s, 2H, NH_2), 6.69 (s, 2H, NH_2), 7.07 (t, *J* = 7.6 Hz, 1H, Ar), 7.25-7.51 (m, 5H, Ar), 7.76-7.93 (m, 3H, Ar), 8.02 (d, *J* = 8.4 Hz, 1H Ar), 10.82 (s, 1H, OH), 11.95 (s, 1H, NH) ppm. **^{13}C-NMR** (100 MHz, DMSO-d6) δ 26.76, 70.47, 89.08, 114.46, 115.21, 115.58, 116.51, 117.06 (2C), 121.56, 122.13, 122.68, 124.08, 126.83, 128.30, 128.44 (2C), 130.18, 130.78, 131.32, 137.26, 149.24, 156.86, 159.17, 160.96, 163.90. **MS (EI, 70 eV) m/z (%):** 429 ($[M-H_2O]^+$, 5), 410 (14), 364 (19), 287 (100), 258 (6), 221 (13), 161 (35), 144 (38), 92 (12), 63 (13). **HRMS-ESI:** $[M+H]^+$, calcd for $C_{26}H_{18}N_5O_3$ 448.1410, found 448.1401.

Isolation of the intermediate ***5***

Salicylaldehyde **1a** (0.12 g, 1 mmol) and 2-aminoprop-1-ene-1,1,3-tricarbonitrile **2** (0.13 g, 1 mmol) were stirred at room temperature in 4 ml of ethanol–pyridine (3:1) mixture for 2 h. After the reaction was completed, the solid was filtered, washed with well-chilled methanol (3 × 2 mL) and dried to isolate pure 2-(amino(2-imino-2*H*-chromen-3-yl)-methylene)malononitrile **5**.

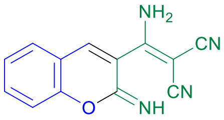

5, 88%

2-(amino-(2-imino-2*H*-chromen-3-yl)methylene)malononitrile, (Yellow solid, 0.208 g, yield 88 %), m.p. 272–273 °C (decomp.) (lit[1] m.p. 271–272 °C (decomp.)). **^{1}H-NMR** (300 MHz, DMSO-*d6*) δ 7.11–7.31 (m, 2H, 2CH Ar), 7.45–7.63 (m, 2H, 2CH Ar), 7.75 (s, 1H, CH), 8.65 (s, 1H, NH), 8.93 (br.s, 1H, N*H*H), 8.95 (br.s, 1H, NH*H*) ppm.

Synthesis of chromeno[2,3-b]pyridine ***4a*** *from 2-(amino(2-imino-2H-chromen-3-yl)methylene)-malononitrile* ***5*** *and 4-hydroxyquinolin-2(1H)-one* ***3***

2-(Amino(2-imino-2*H*-chromen-3-yl)methylene)malononitrile **5** (0.24 g, 1 mmol) and 4-hydroxyquinolin-2(1*H*)-one (0.16 g, 1 mmol) **3** were refluxed in 4 ml of ethanol–pyridine (3:1) mixture for 2 h. After the reaction was completed, the solid was filtered, washed with well-chilled methanol (3 × 2 mL) and dried to isolate pure chromeno[2,3-*b*]pyridine **4a**.

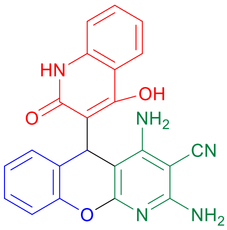

4a, 95%

2,4-Diamino-5-(4-hydroxy-2-oxo-1,2-dihydroquinolin-3-yl)-5*H*-chromeno[2,3-*b*]pyridine-3-carbonitrile, (White solid, 0.377g, 95%), mp > 350°C (from Py-EtOH), **^{1}H-NMR** (400 MHz, DMSO-*d6*) δ 5.58 (s, 1H, CH), 6.31 (s, 2H, NH_2), 6.44 (s, 2H, NH_2), 6.90-7.05 (m, 3H, Ar), 7.06-7.22 (m, 2H, Ar), 7.28-7.41 (m, 2H, Ar), 7.86 (d, *J* = 7.8 Hz, 1H, Ar), 10.77 (br s, 1H, OH), 11.86 (s, 1H, NH) ppm.

^{1}H and ^{13}C Spectra of Compounds

Figure S1. Compound **4a**

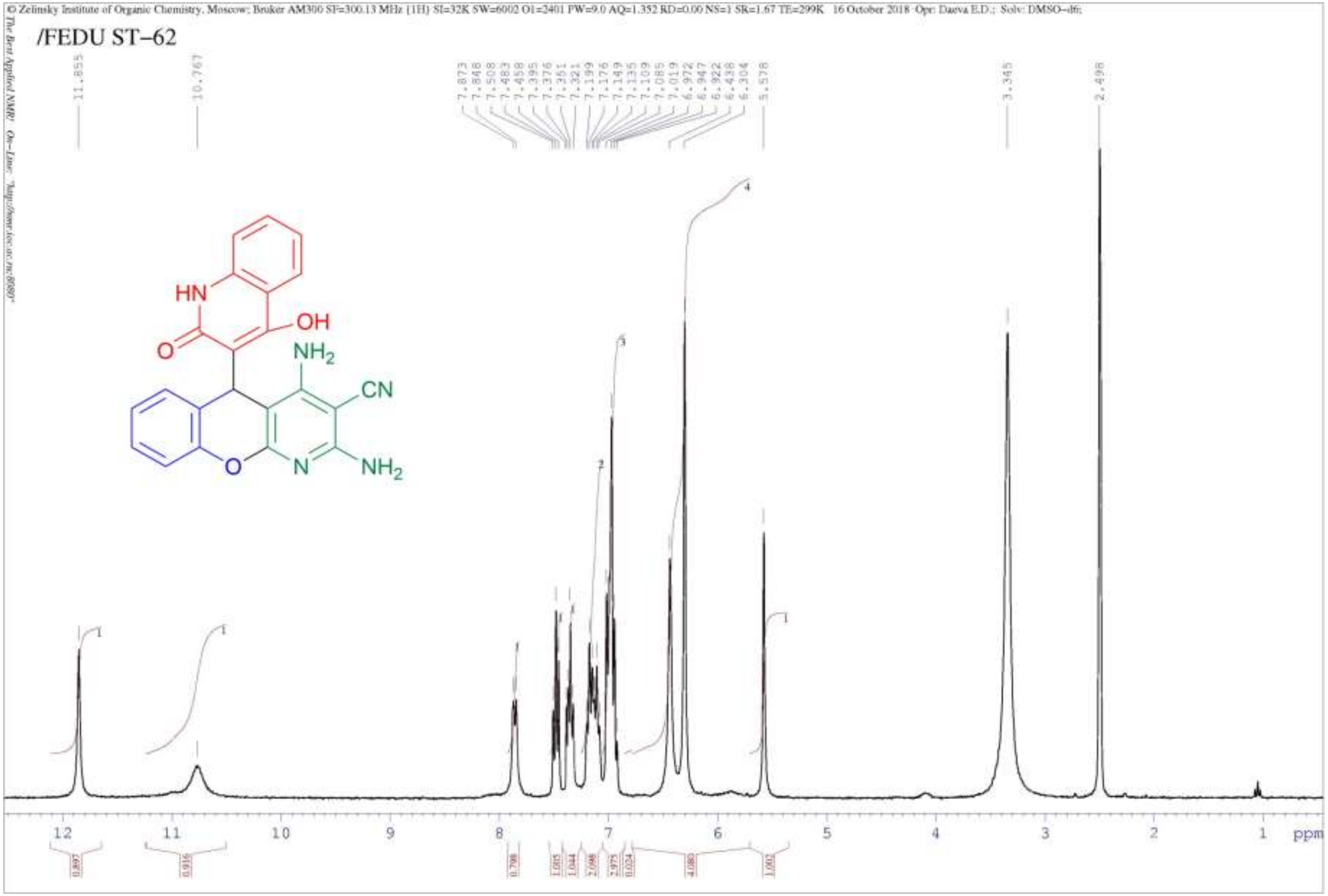

Figure S2. Compound **4a**

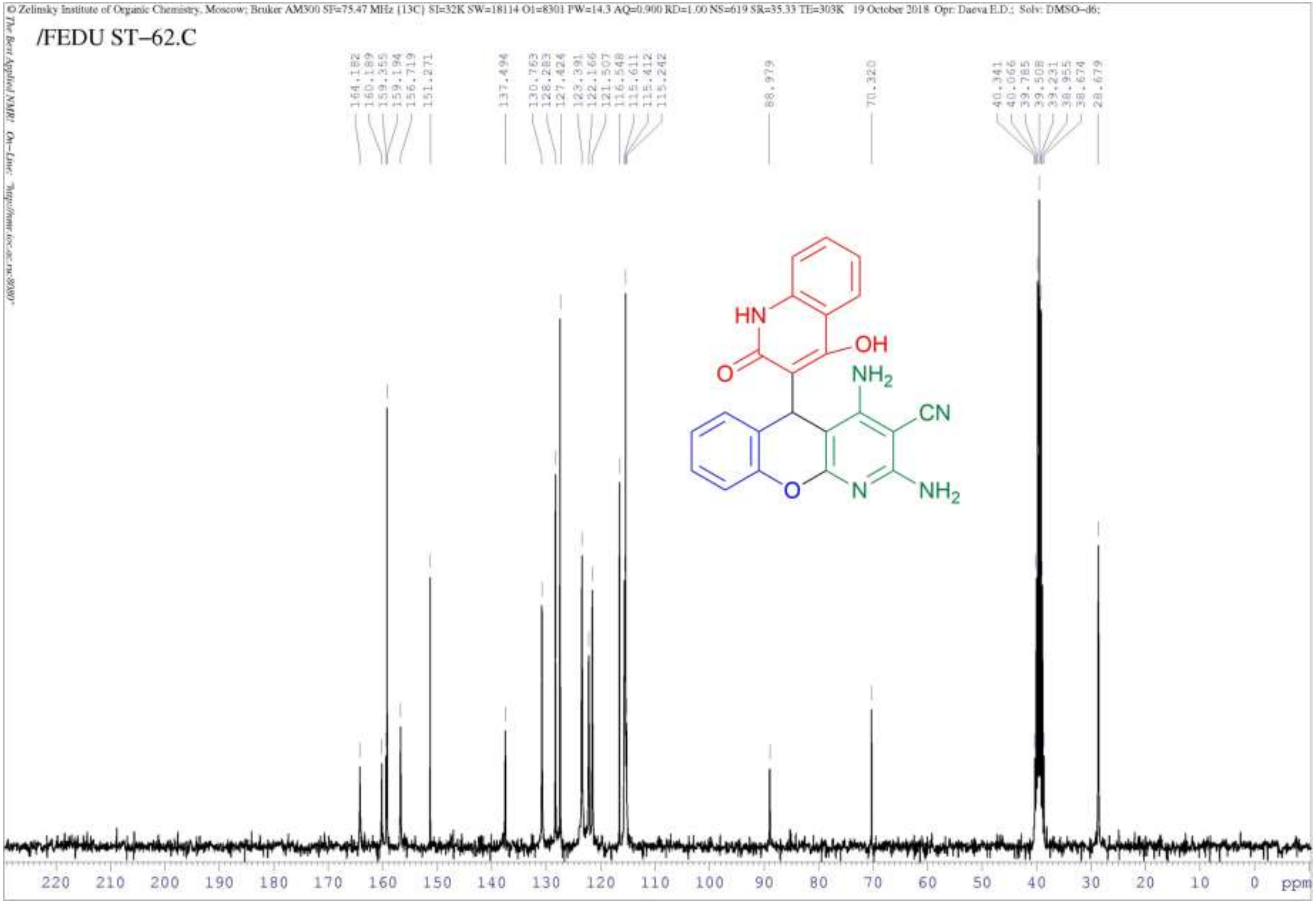

Figure S3. Compound **4b**

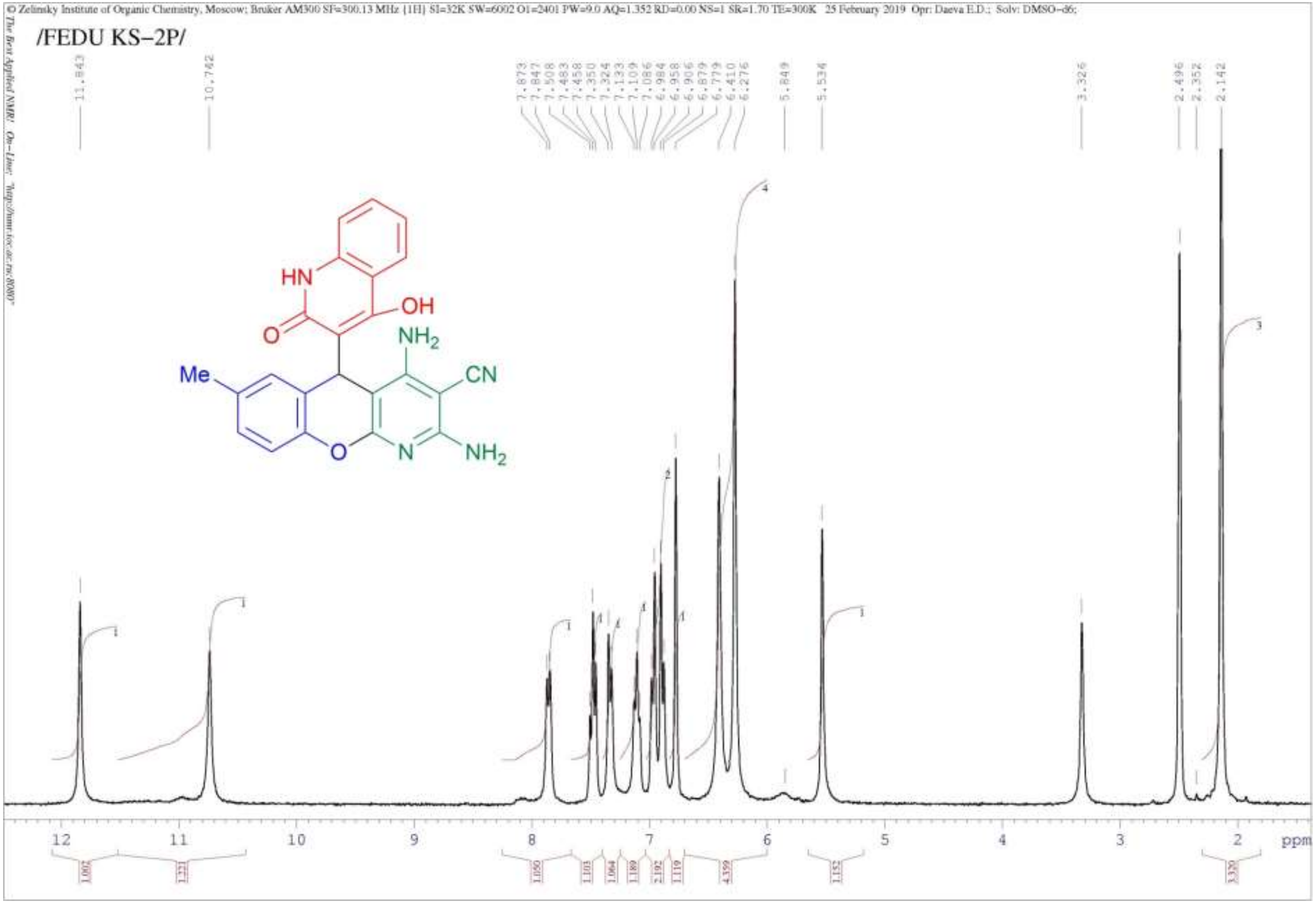

Figure S4. Compound **4b**

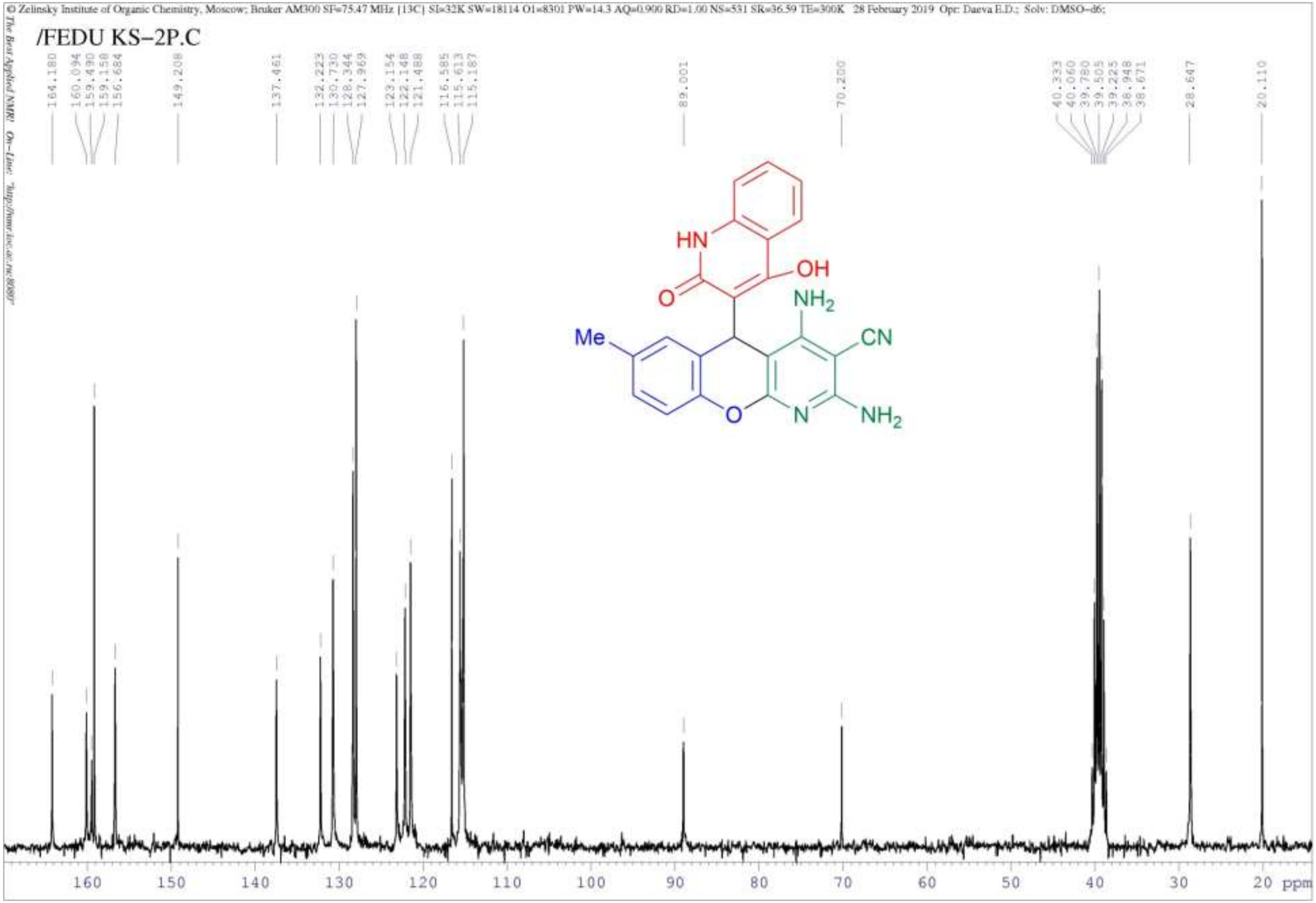

Figure S5. Compound **4c**

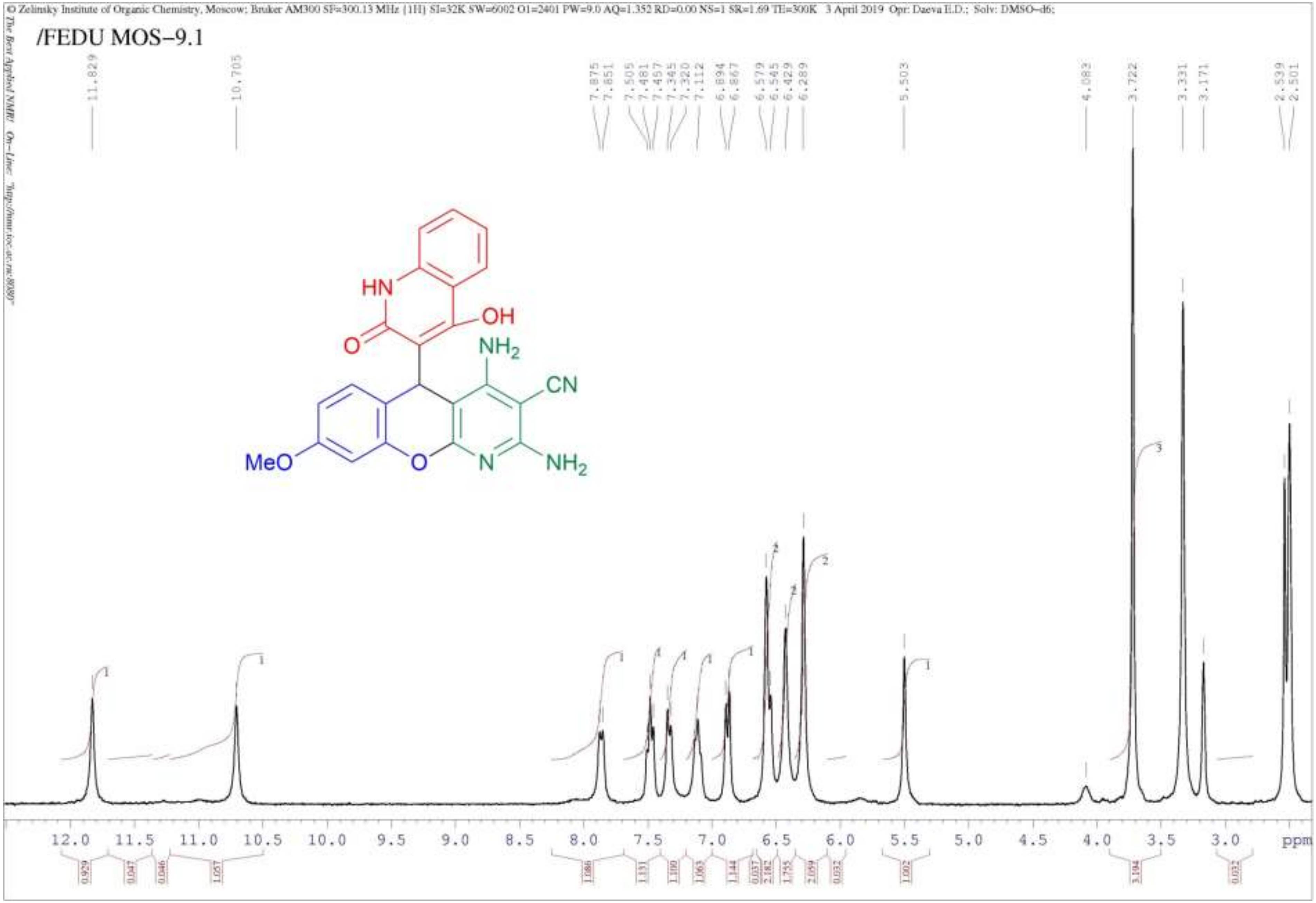

Figure S6. Compound **4c**

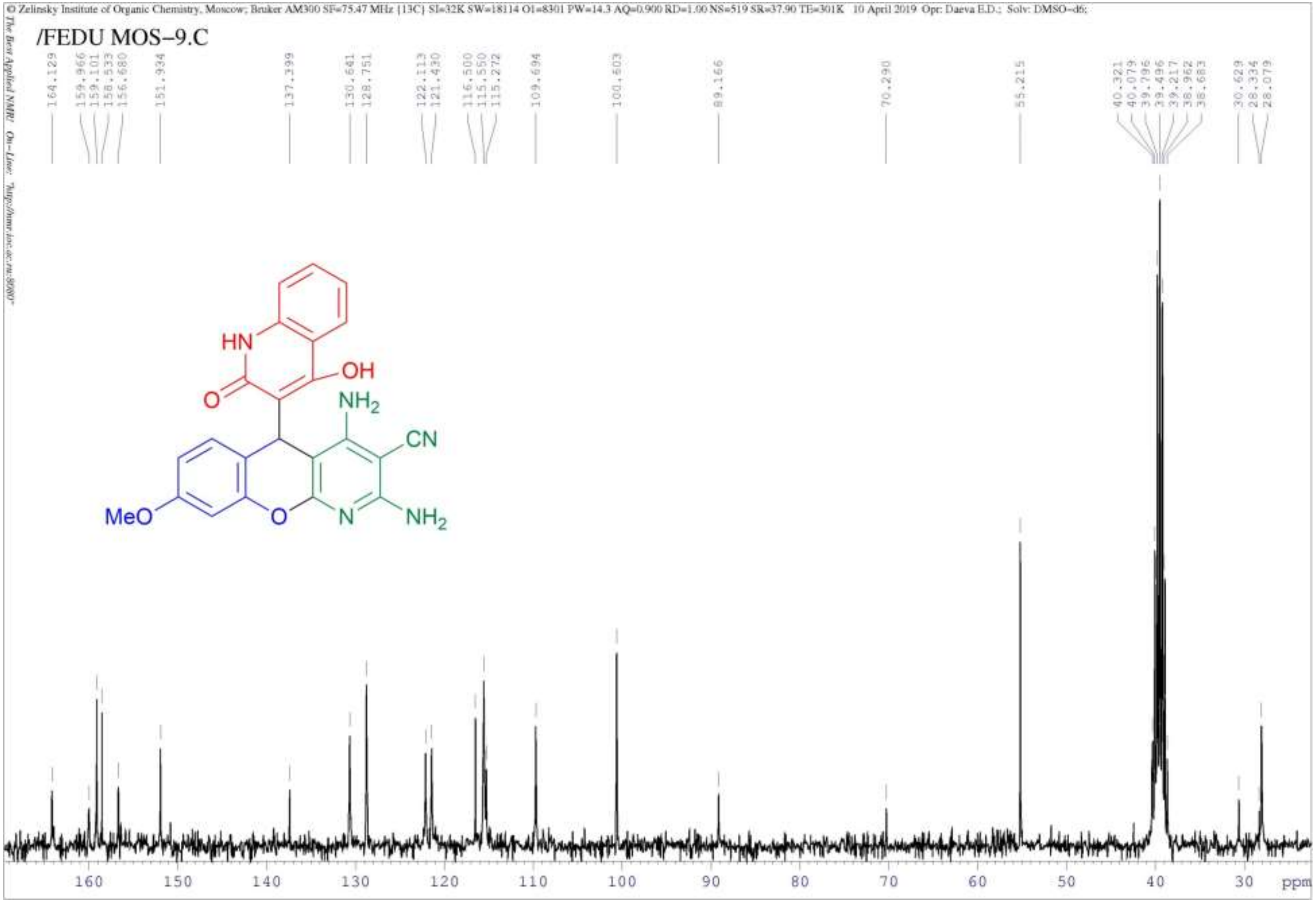

Figure S7. Compound **4d**

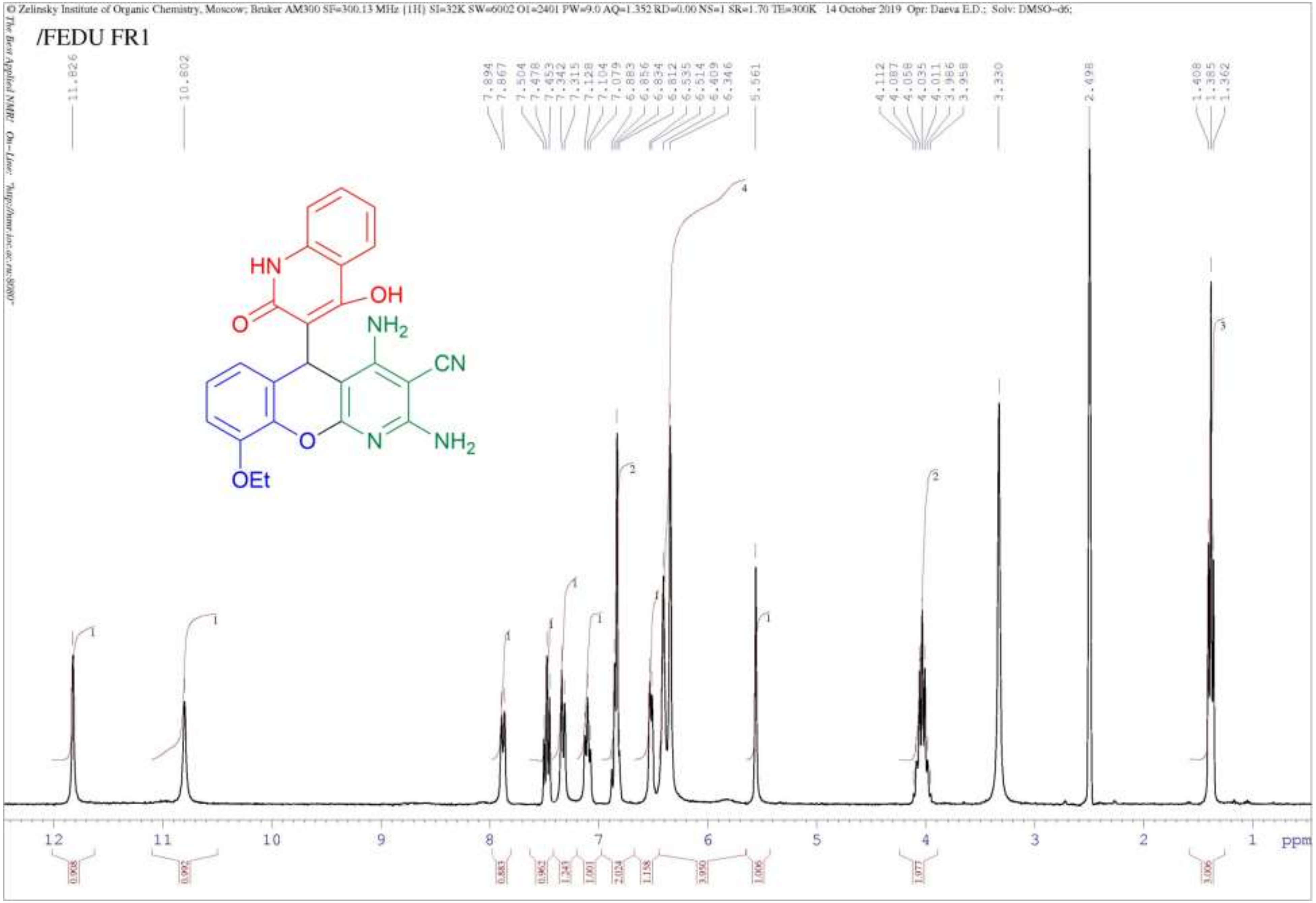

Figure S8. Compound **4d**

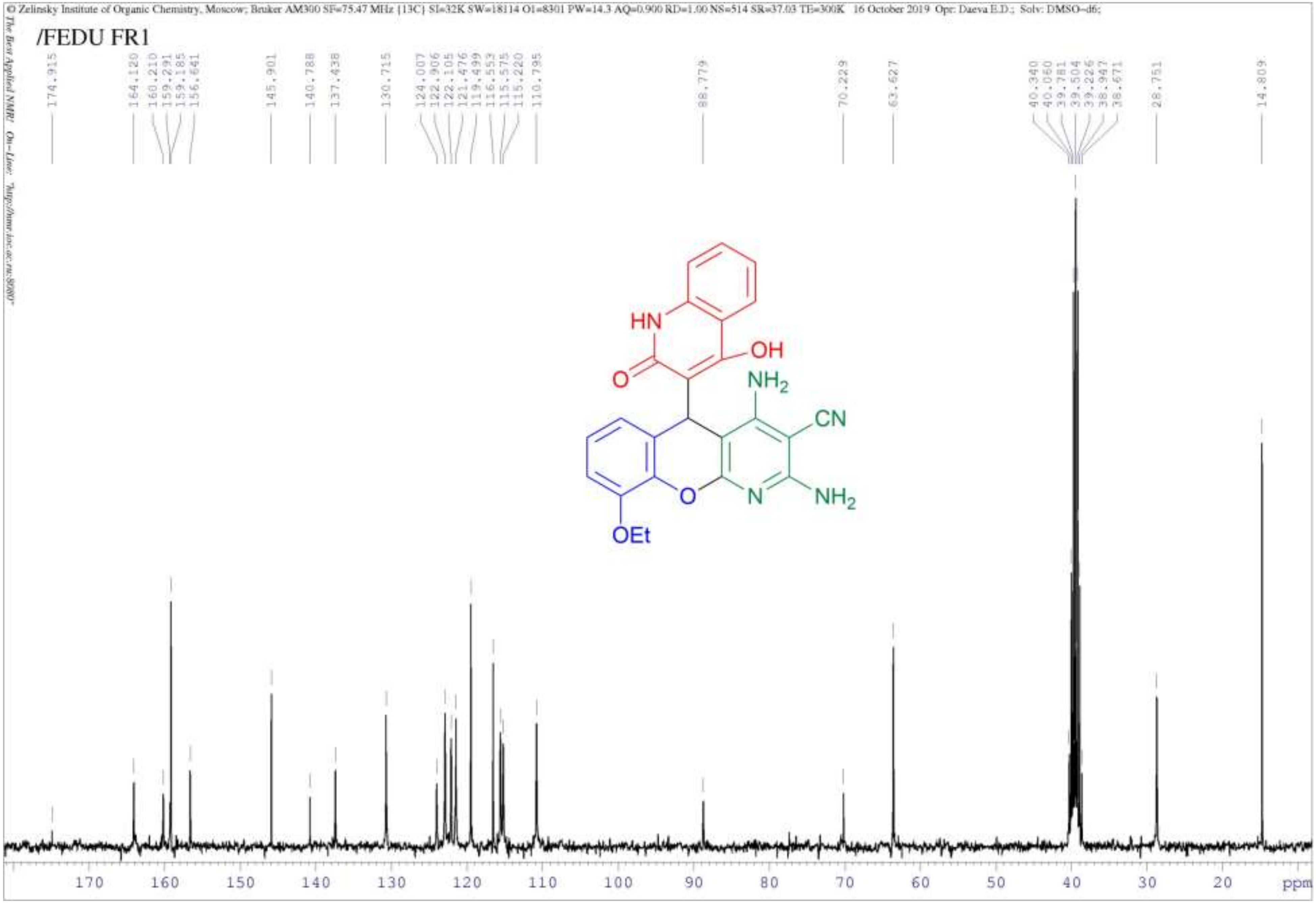

Figure S9. Compound **4e**

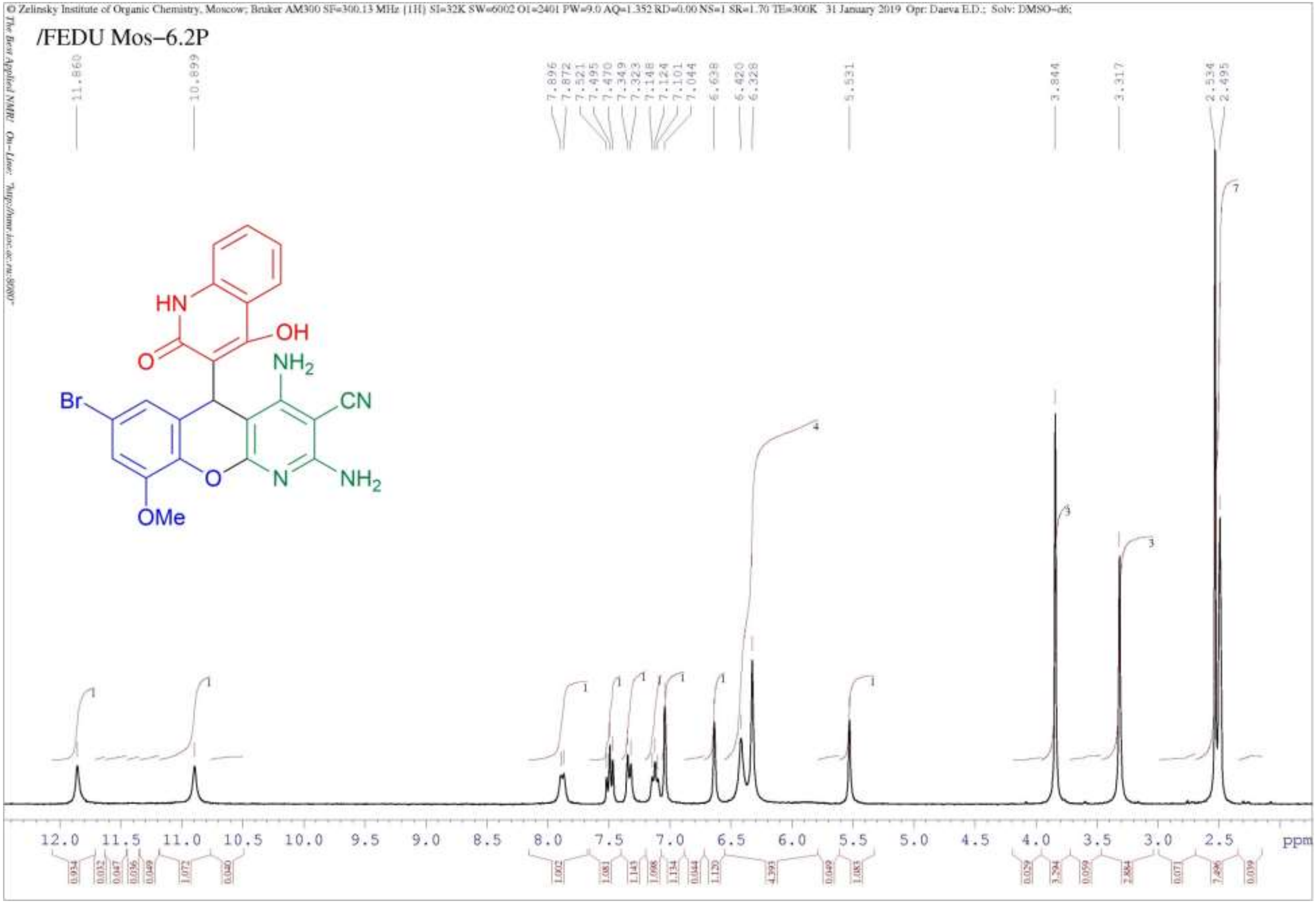

Figure S10. Compound **4e**

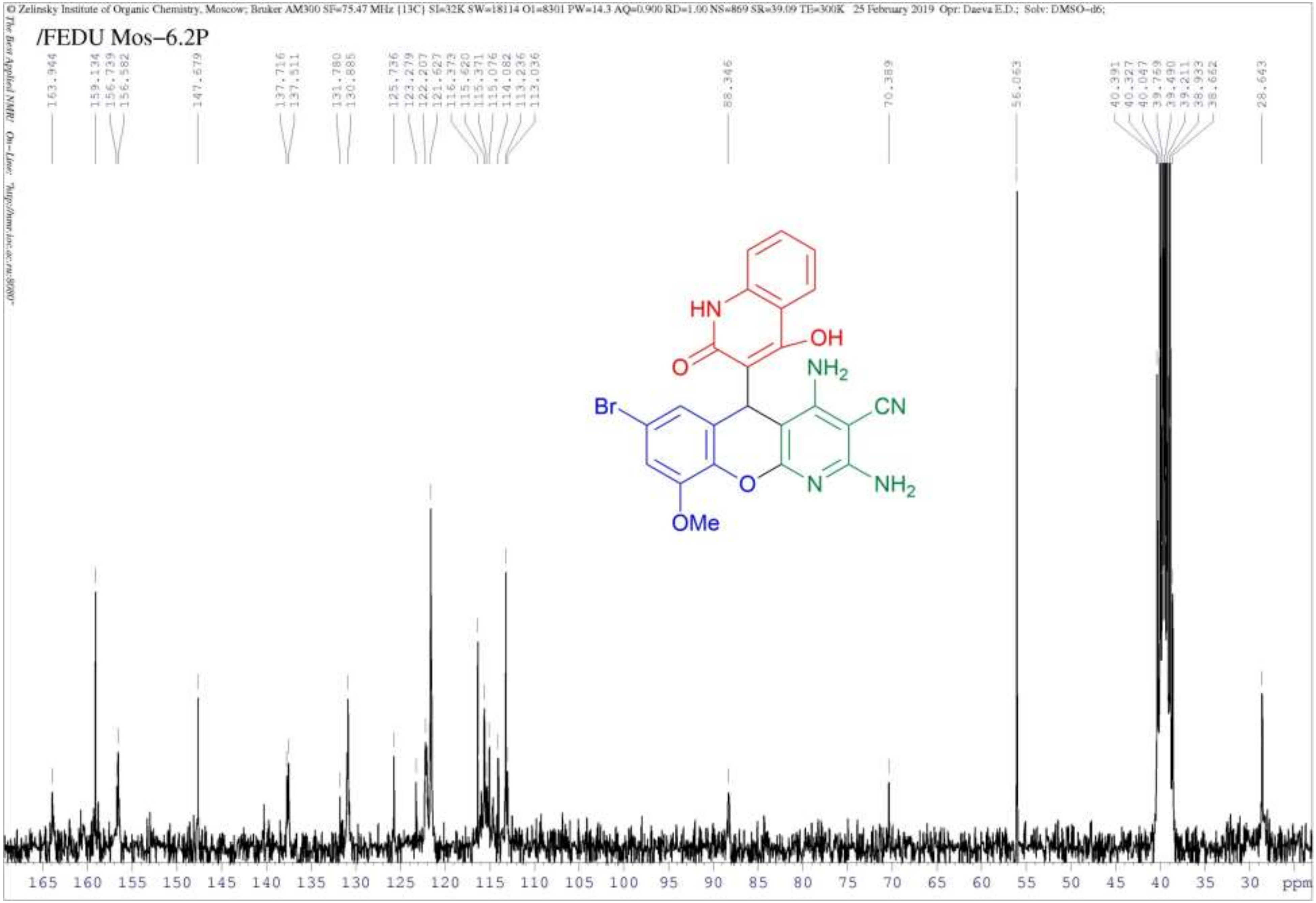

Figure S11. Compound **4f**

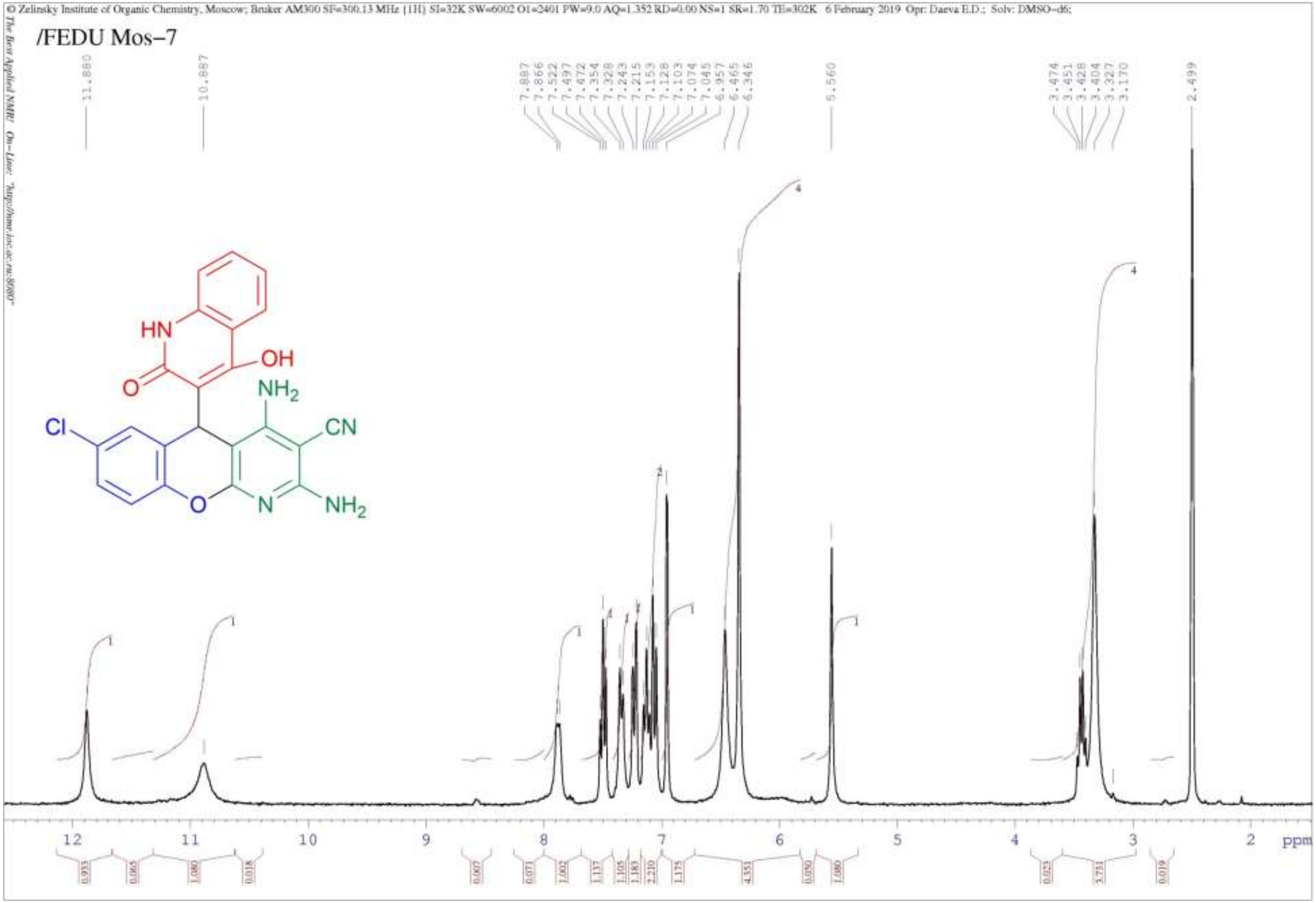

Figure S12. Compound **4f**

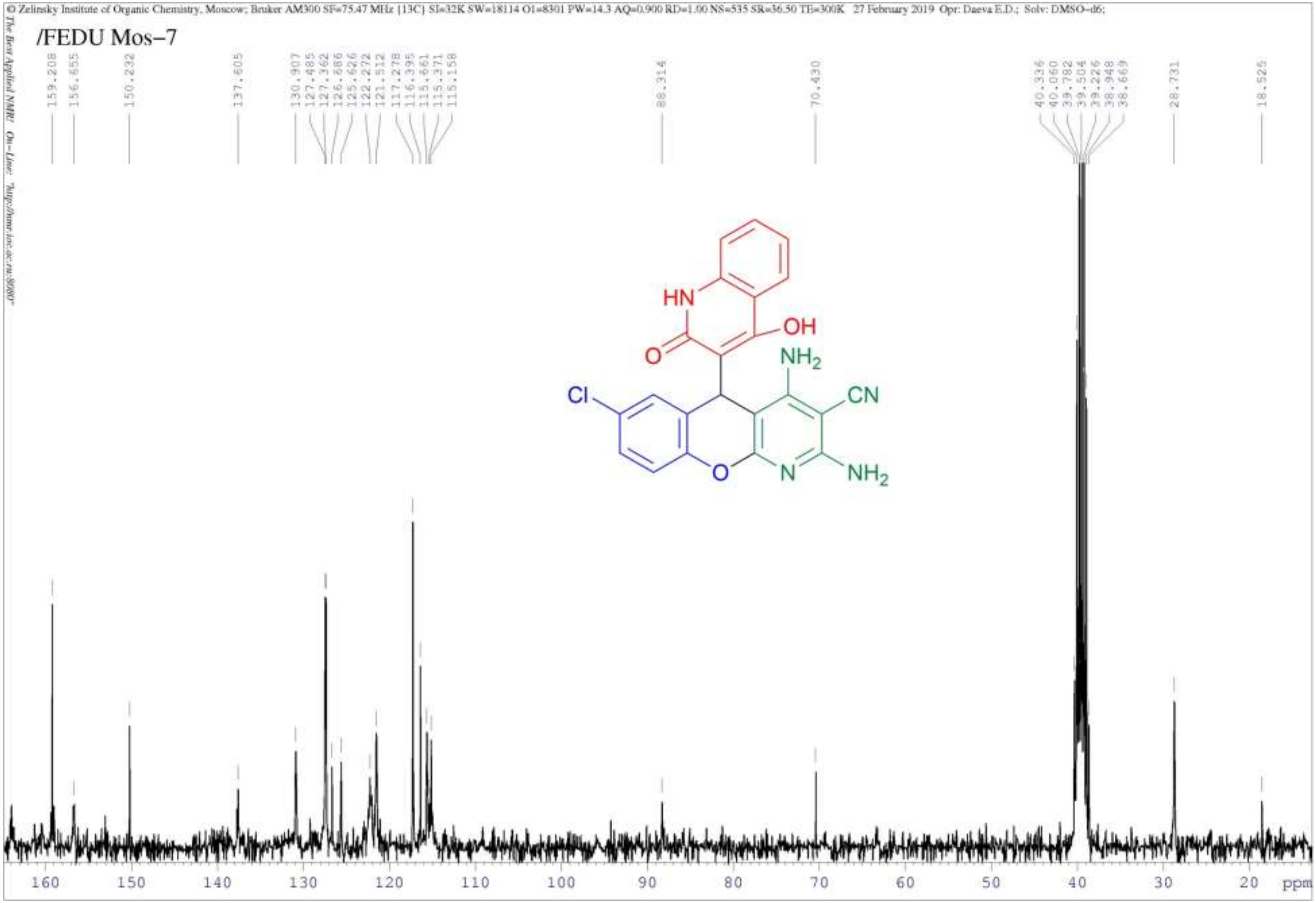

Figure S13. Compound **4g**

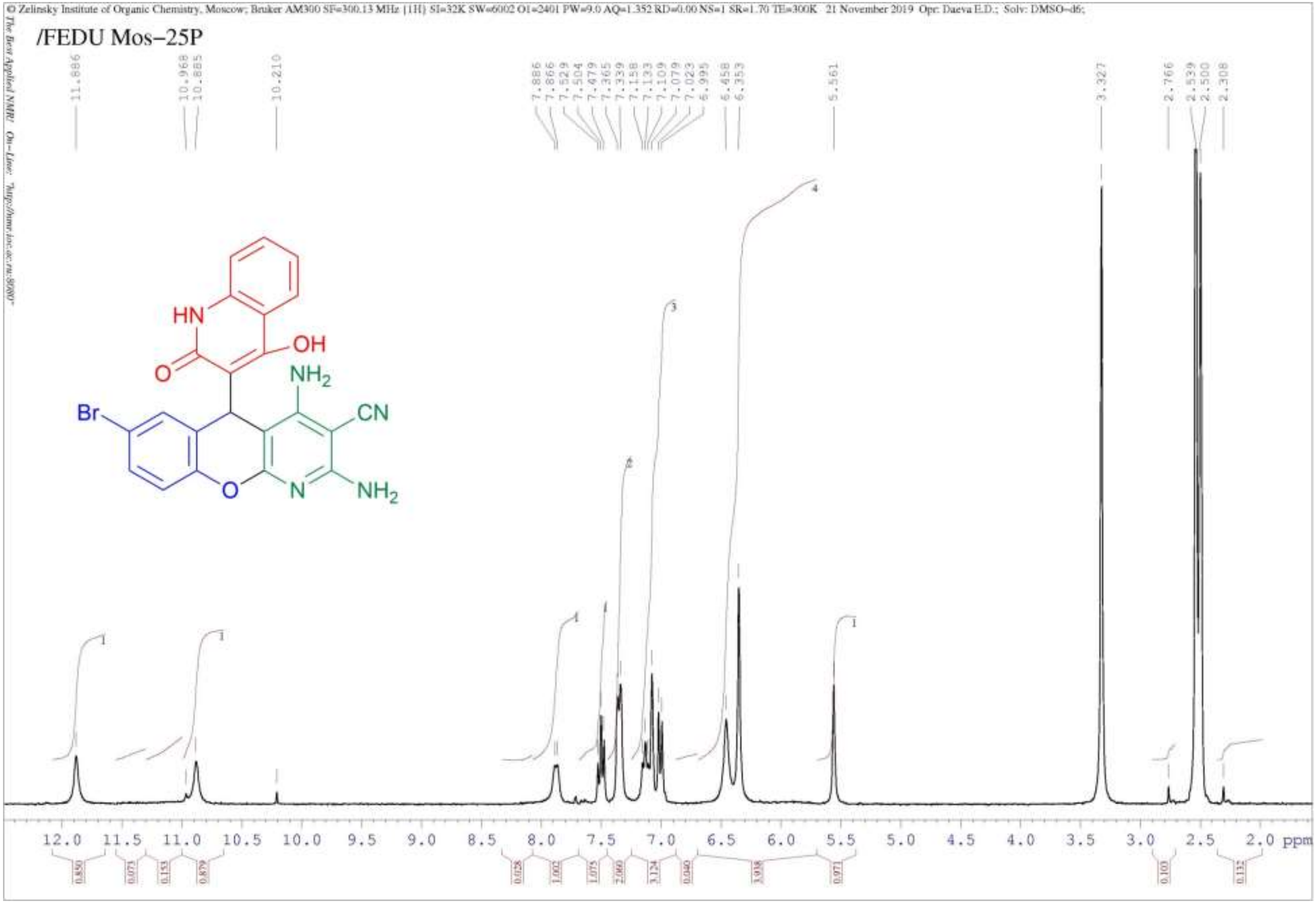

Figure S14. Compound **4g**

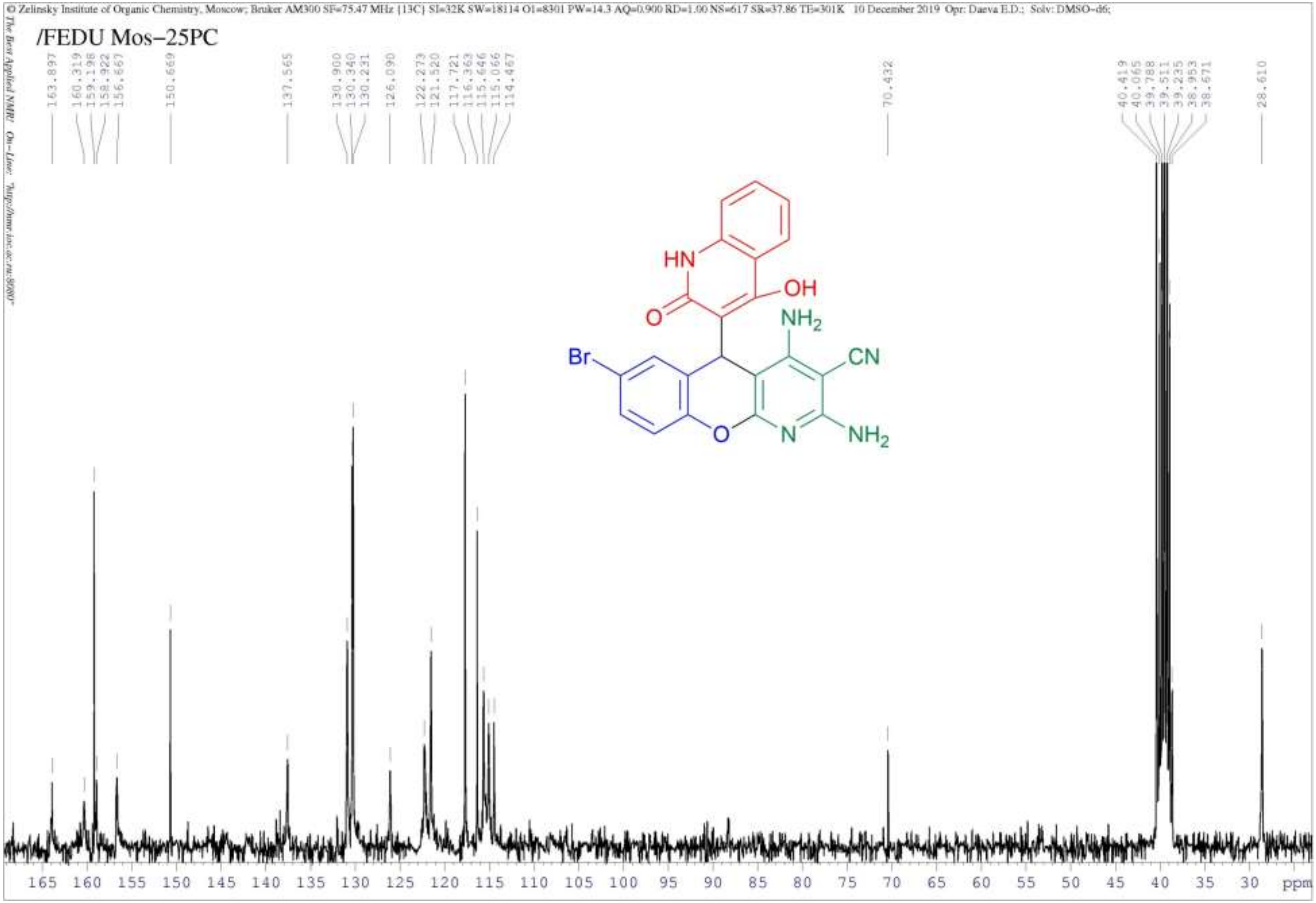

Figure S15. Compound **4h**

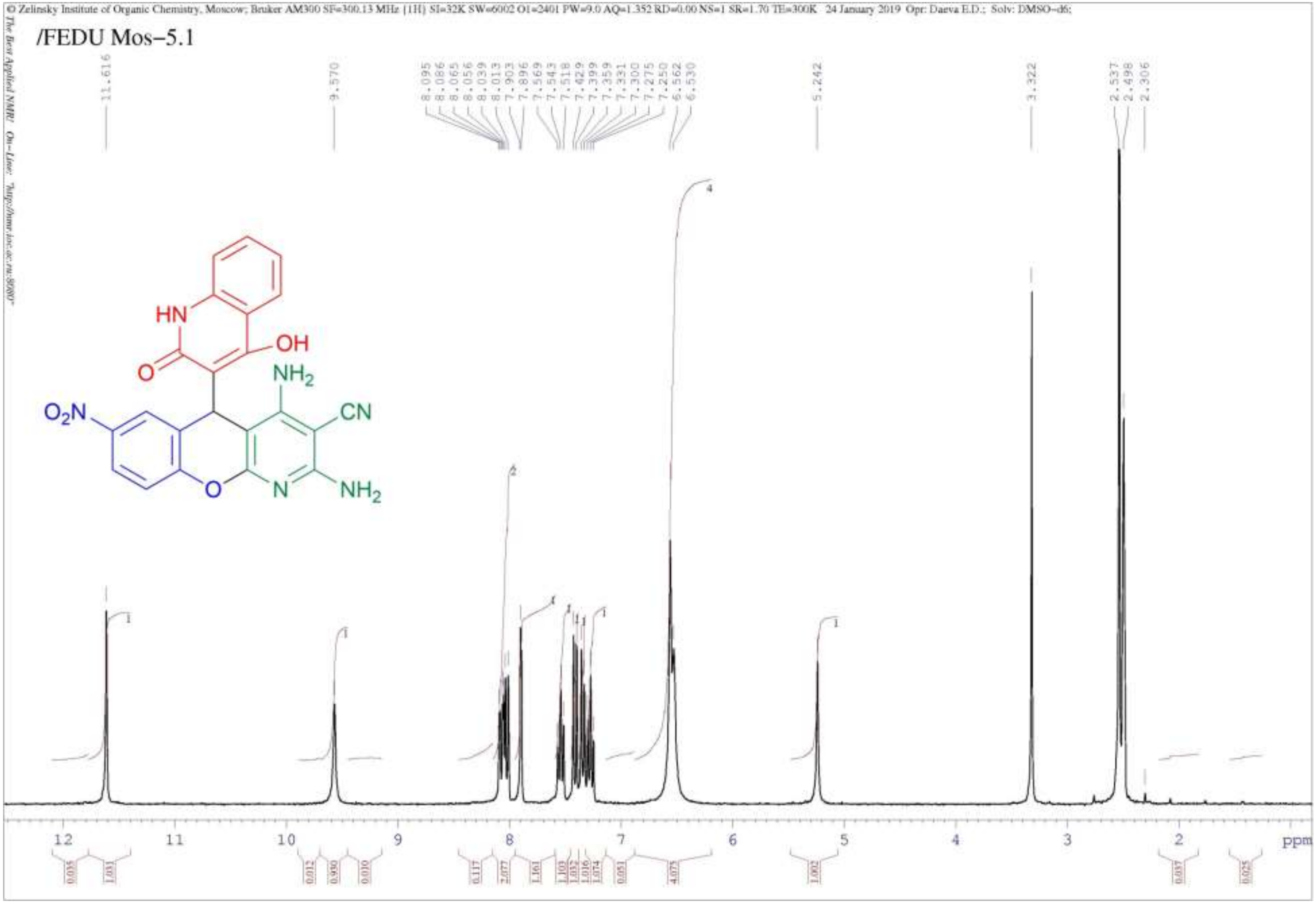

Figure S16. Compound **4h**

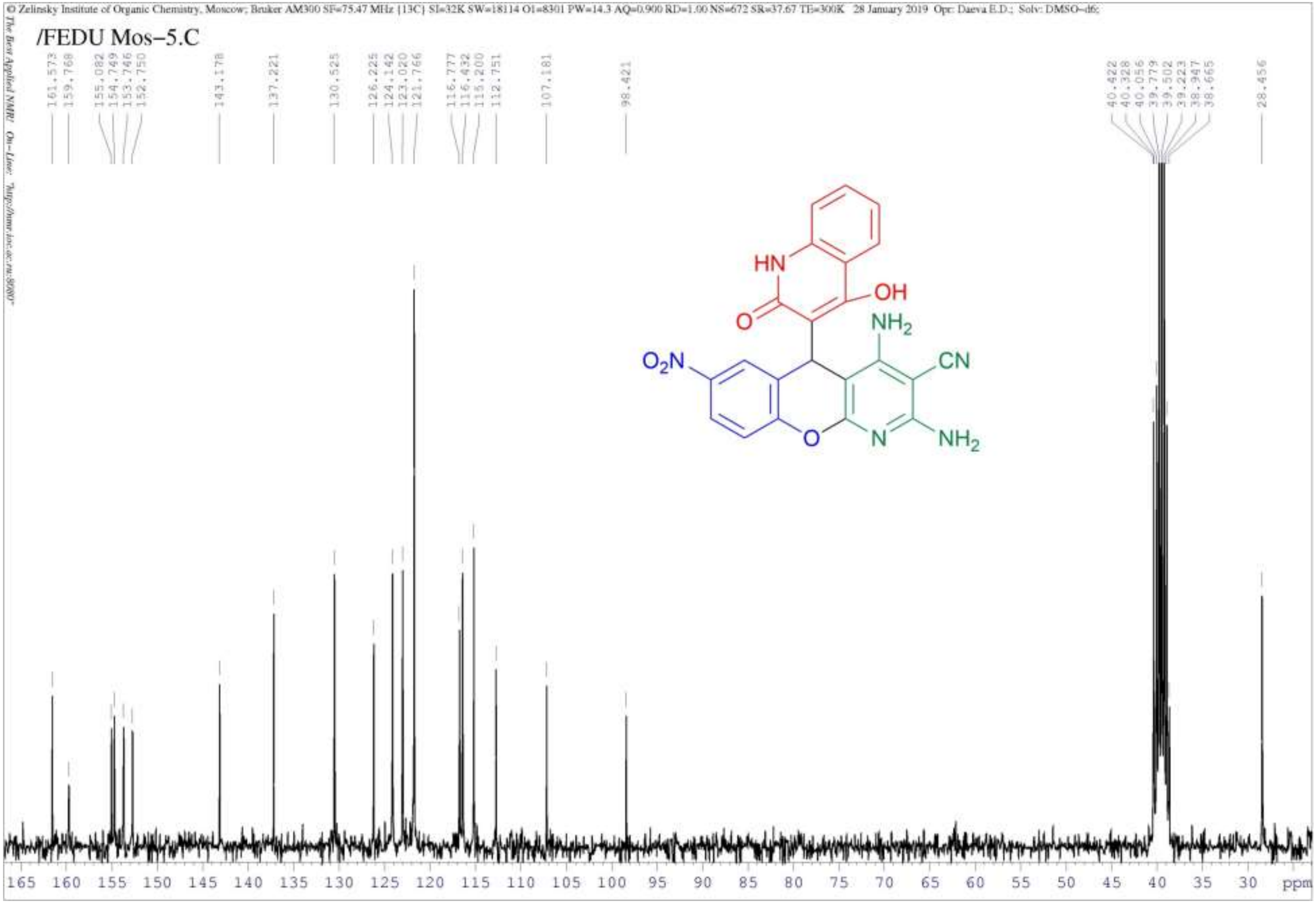

Figure S17. Compound **4i**

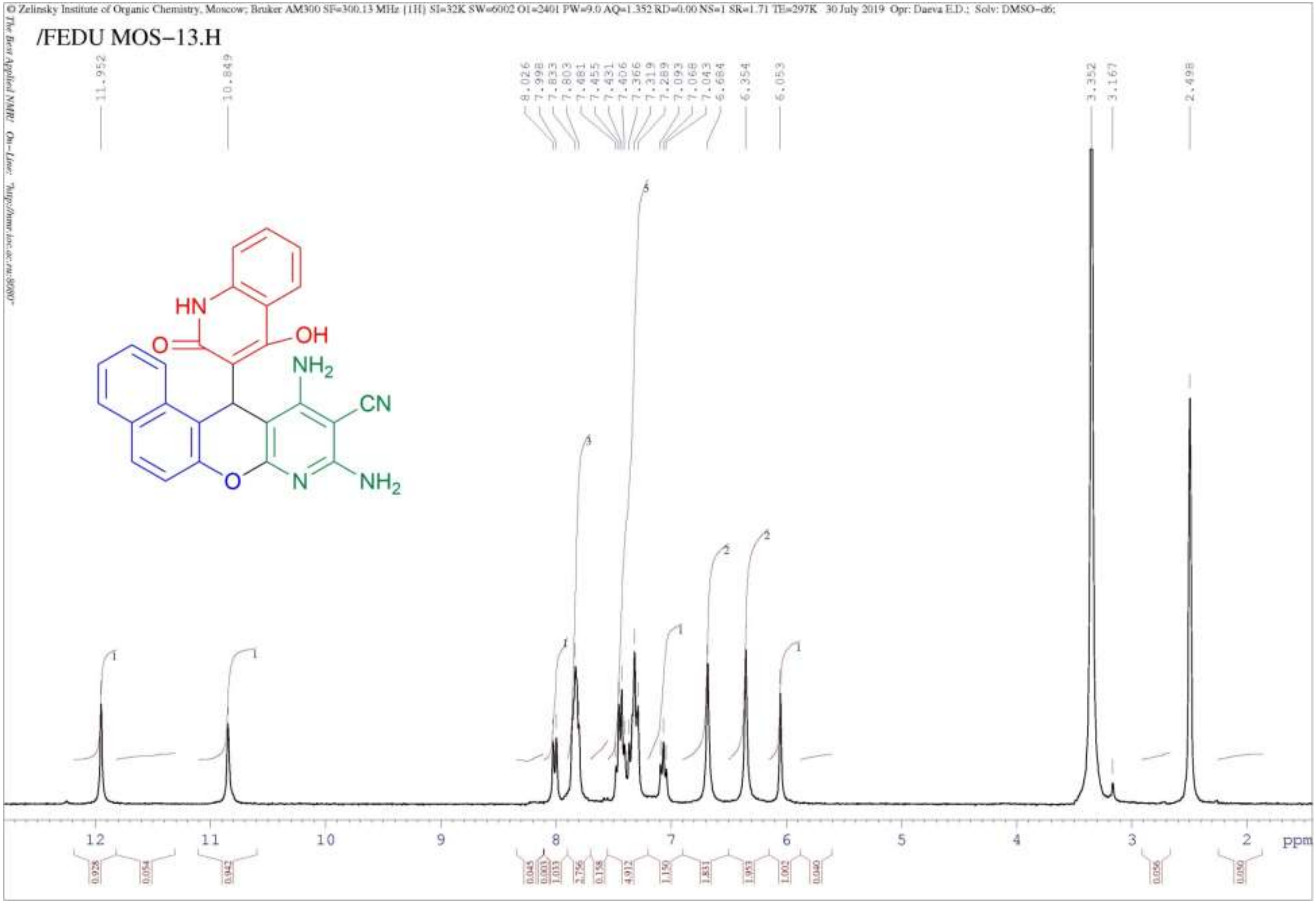

Figure S18. Compound **4i**

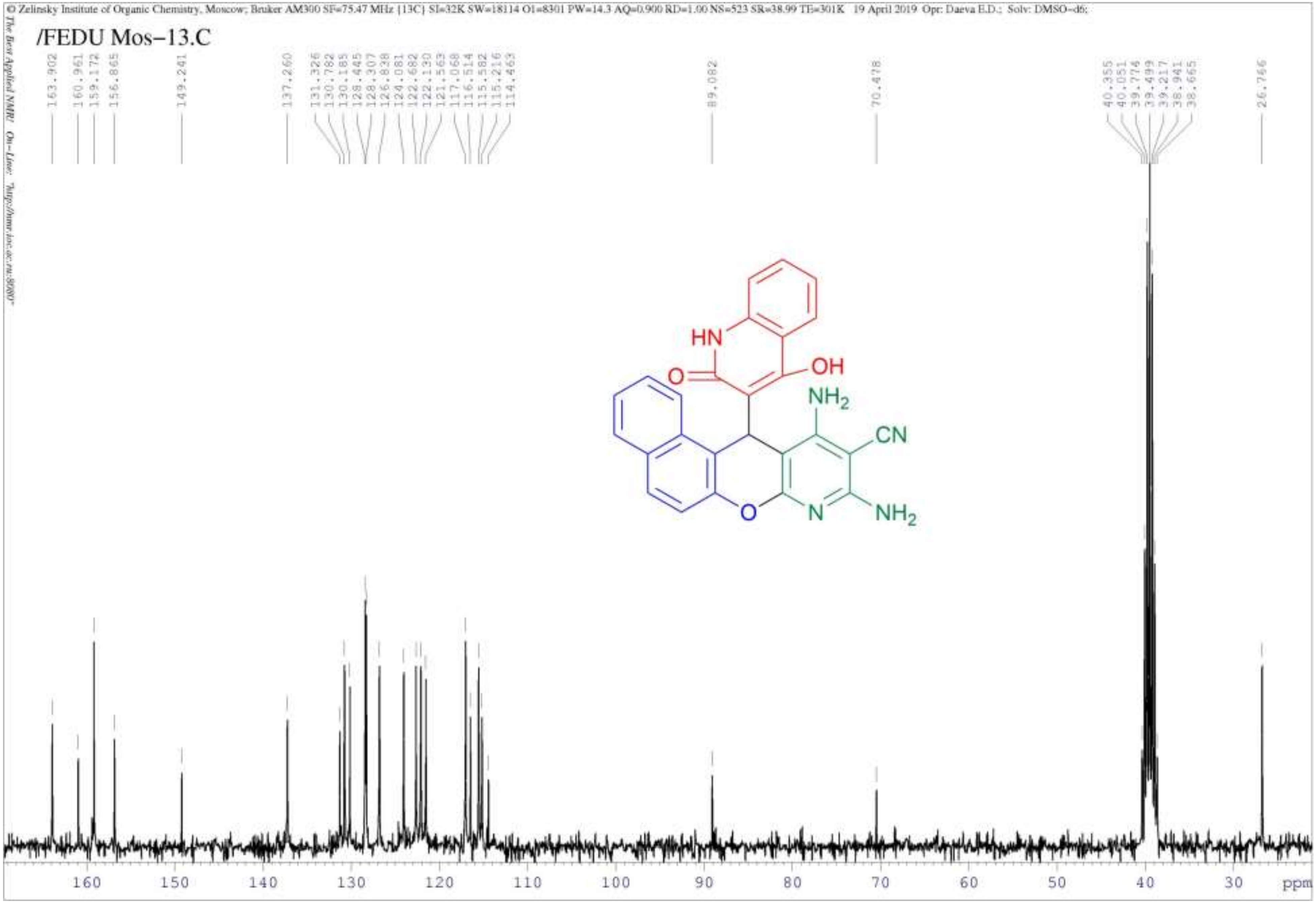

^{1}H NMR monitoring

During monitoring, ^{1}H NMR spectra were recorded with Bruker AV600 spectrometer at 40°C. Chemical shifts values are relative to Me_4Si.

Salicylaldehyde **1a** (0.006 g, 0.05 mmol), 2-aminoprop-1-ene-1,1,3-tricarbonitrile **2** (0.007 g, 0.05 mmol) and 4-hydroxyquinolin-2(1*H*)-one (0.008 g, 0.05 mmol) **3** were dissolved in DMSO-d_6 (0.6 ml) and were placed into ampoule, which was further placed directly into apparatus at 40°C. The spectra were recorded each 12 minute.

The spectra of reference compounds (recorded on Bruker AM-300) and the series of ^{1}H NMR monitoring spectra are presented below.

Figure S19. Salicyclaldehyde **1a**

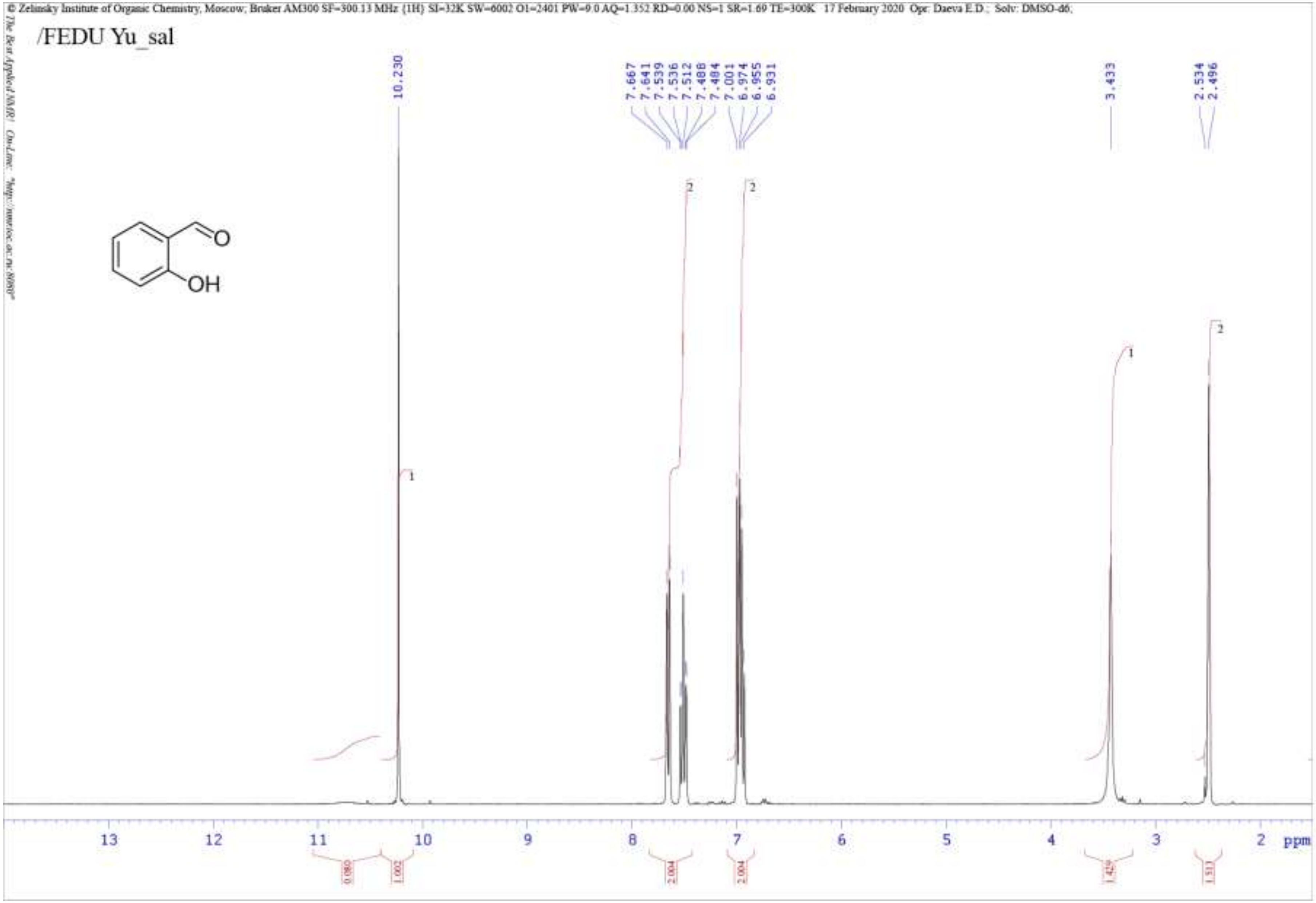

Figure S20. Malononitrile dimer **2**

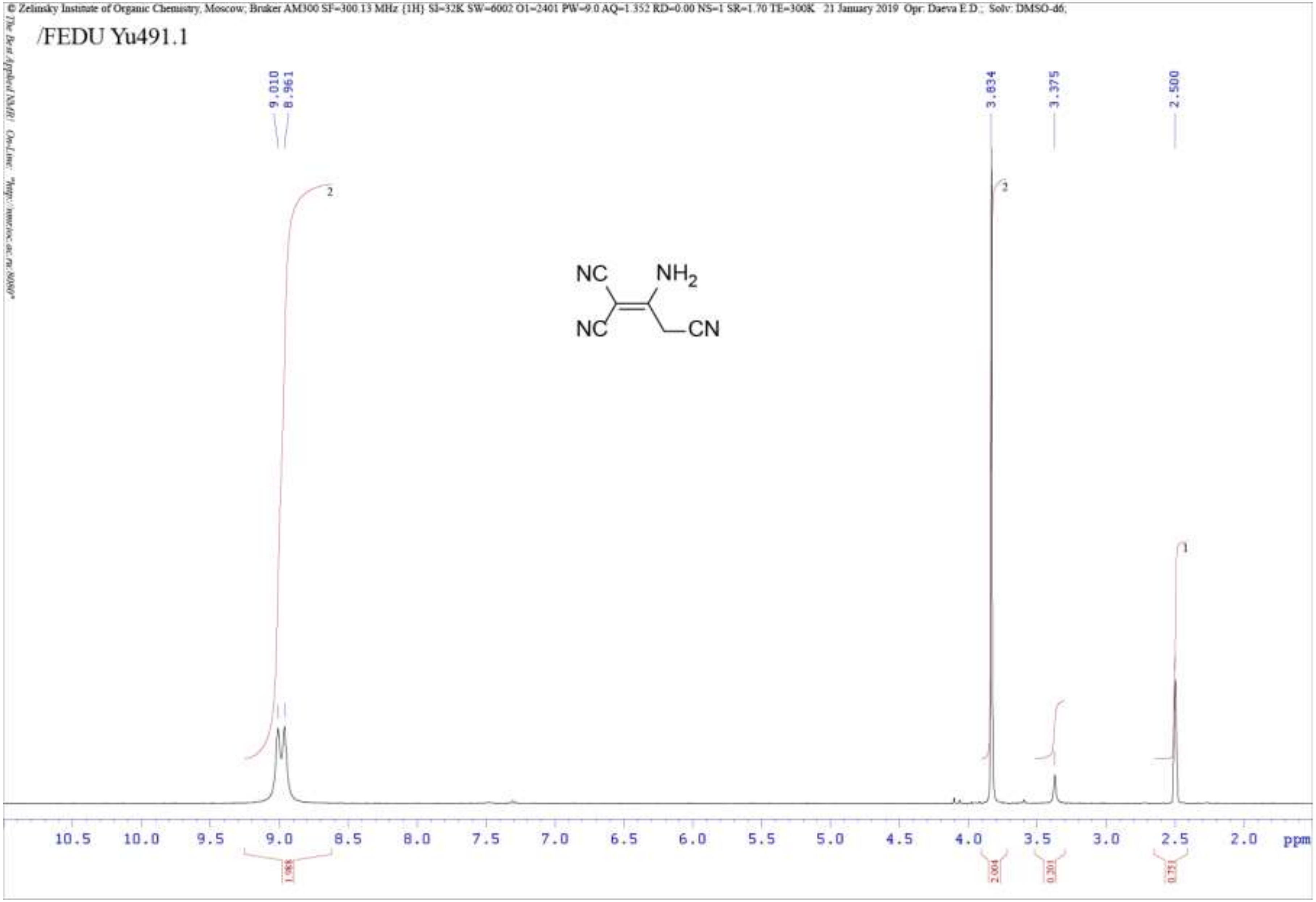

Figure S21. Hydroxyhinolinone **3**

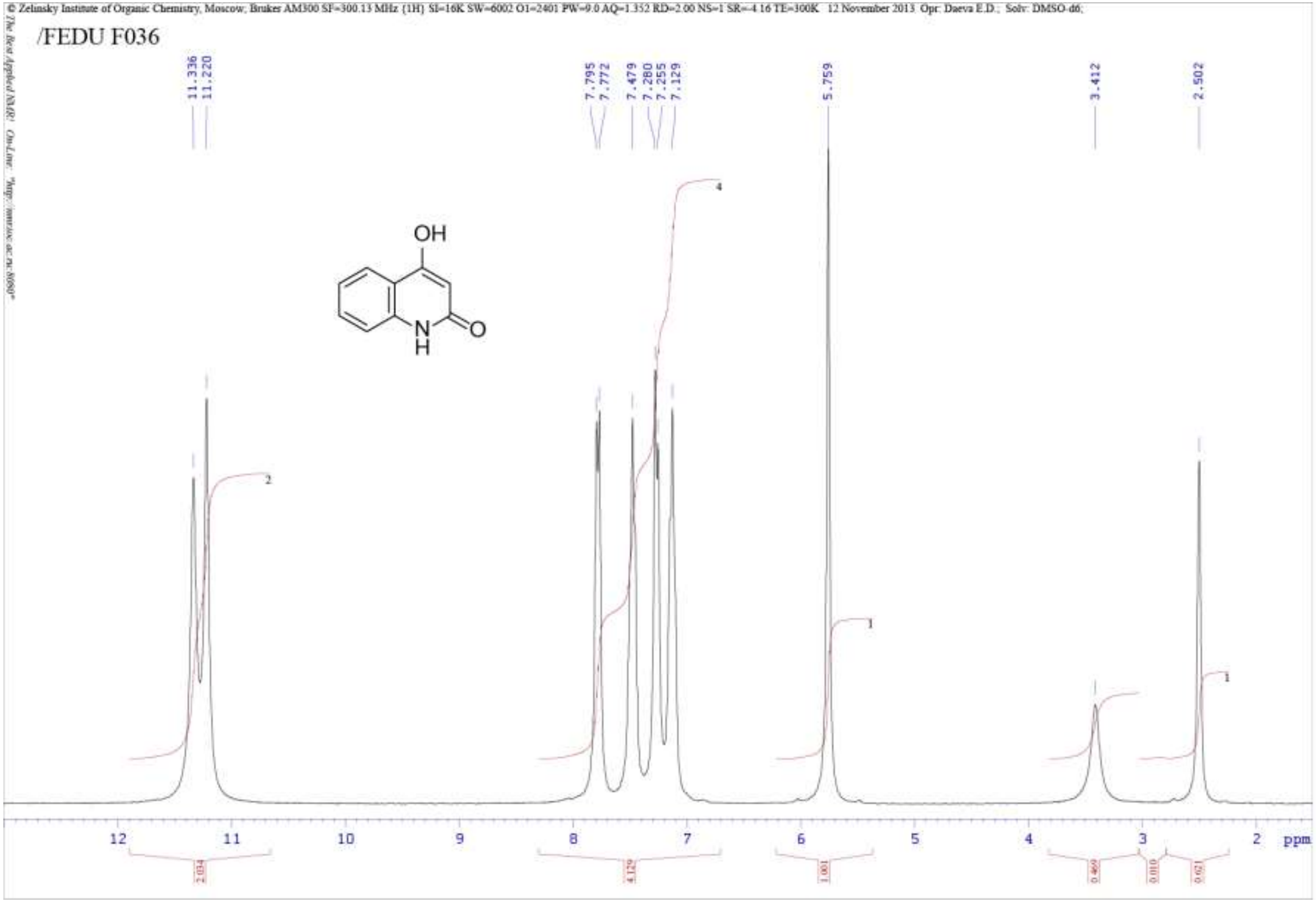

Figure S22. Intermediate **5**

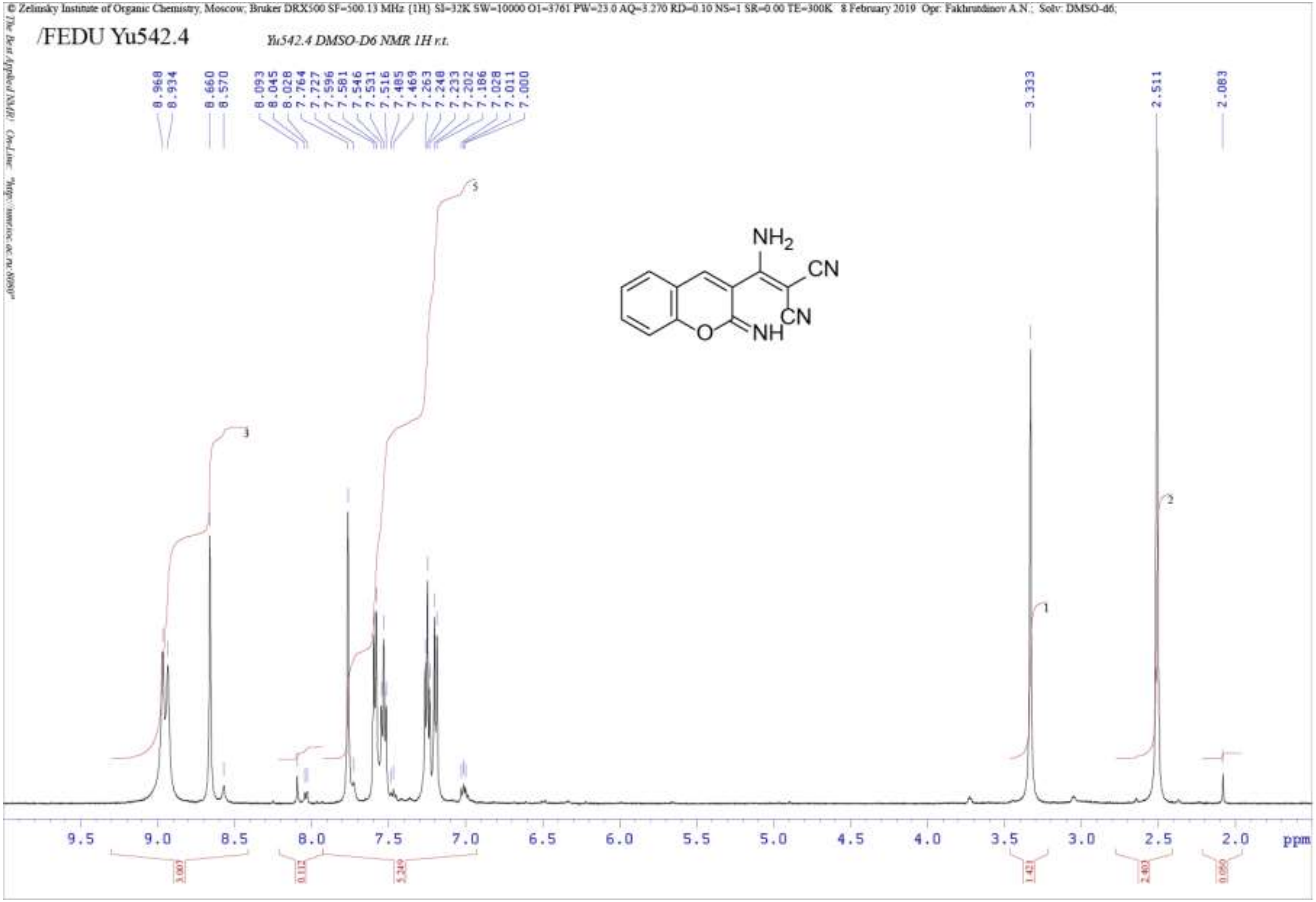

Figure S22. Substituted intermediate **5**

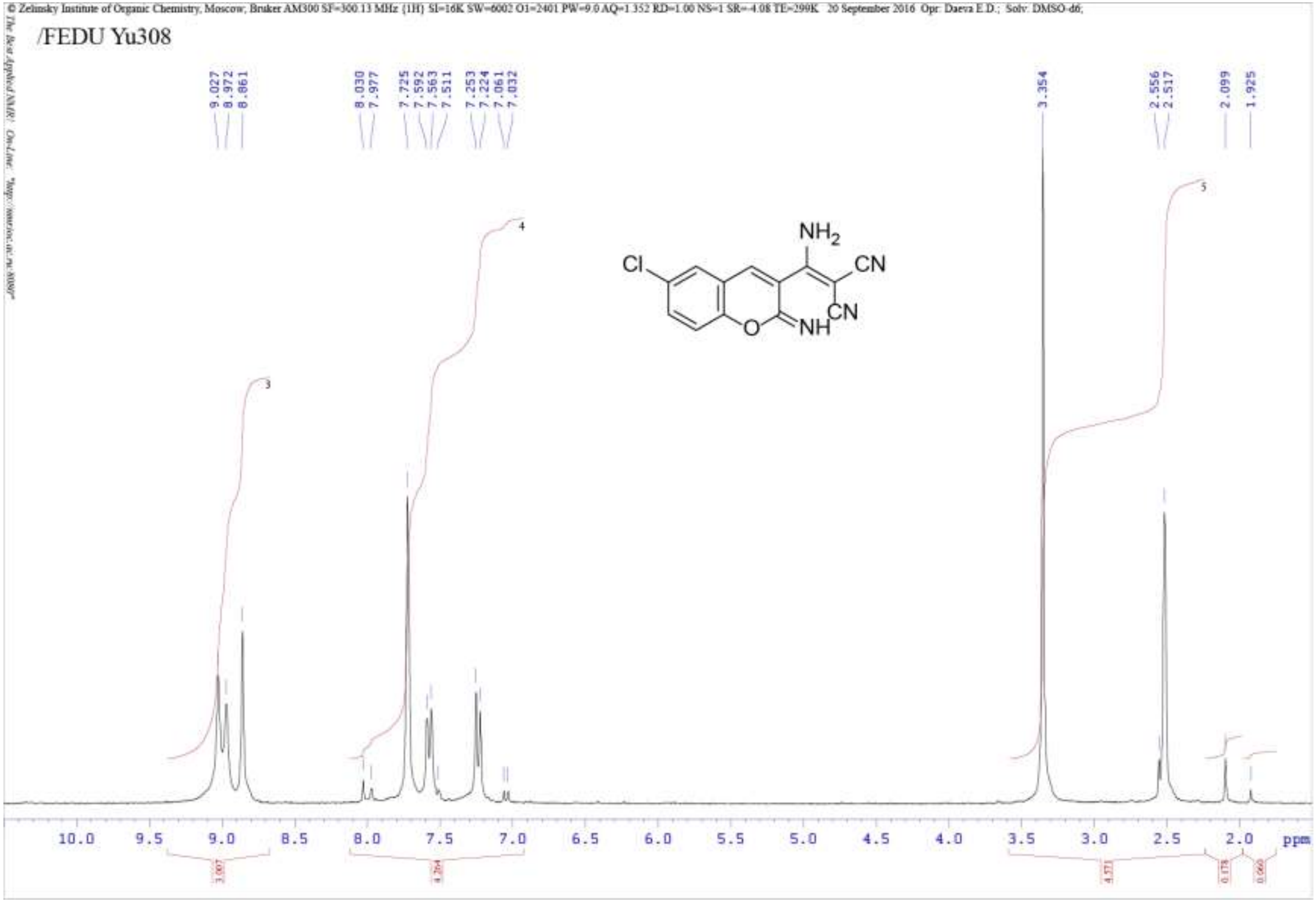

Figures S23-S42. ^{1}H NMR monitoring of the reaction, recorded each 12 minutes of the reaction.

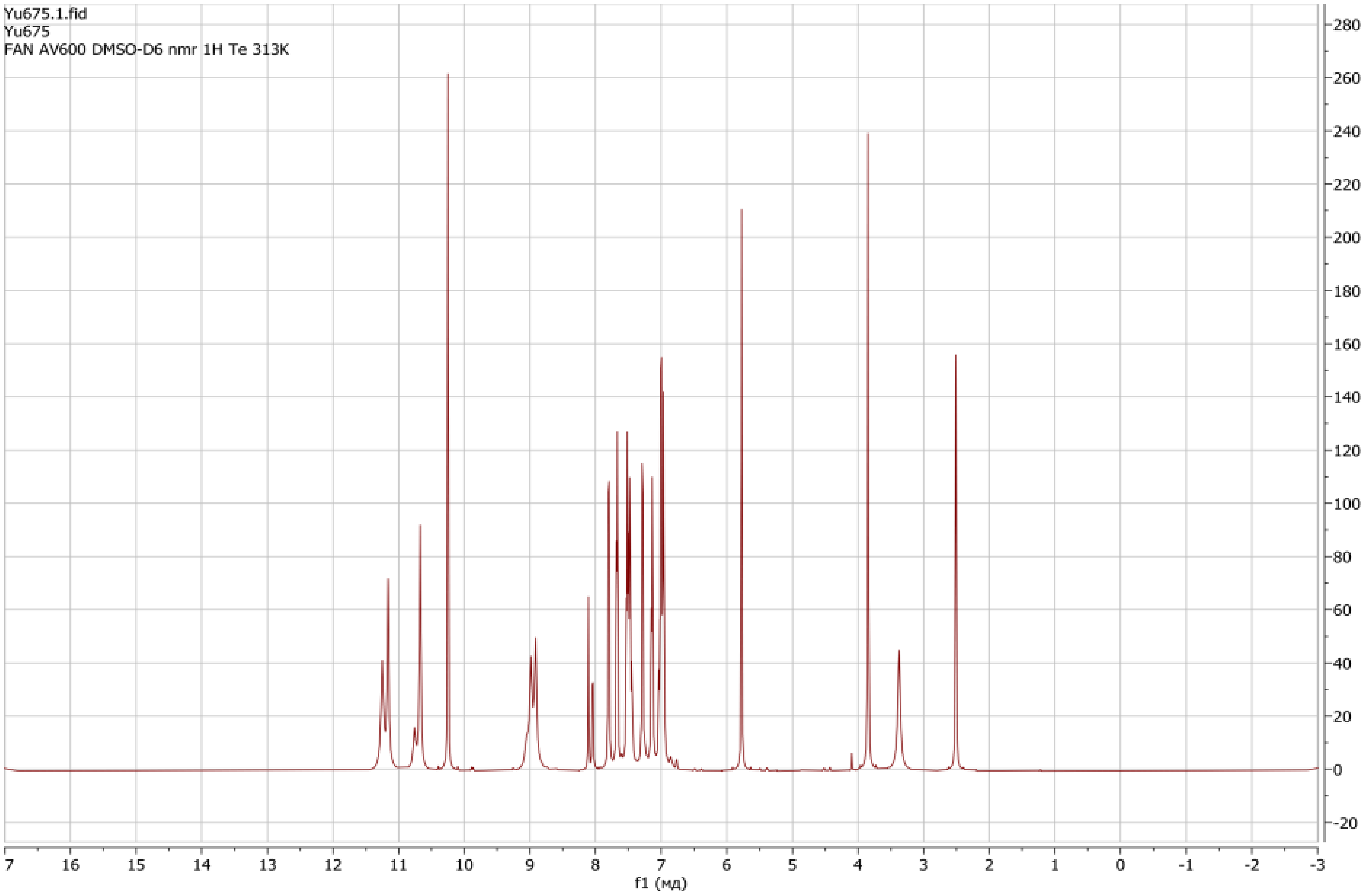

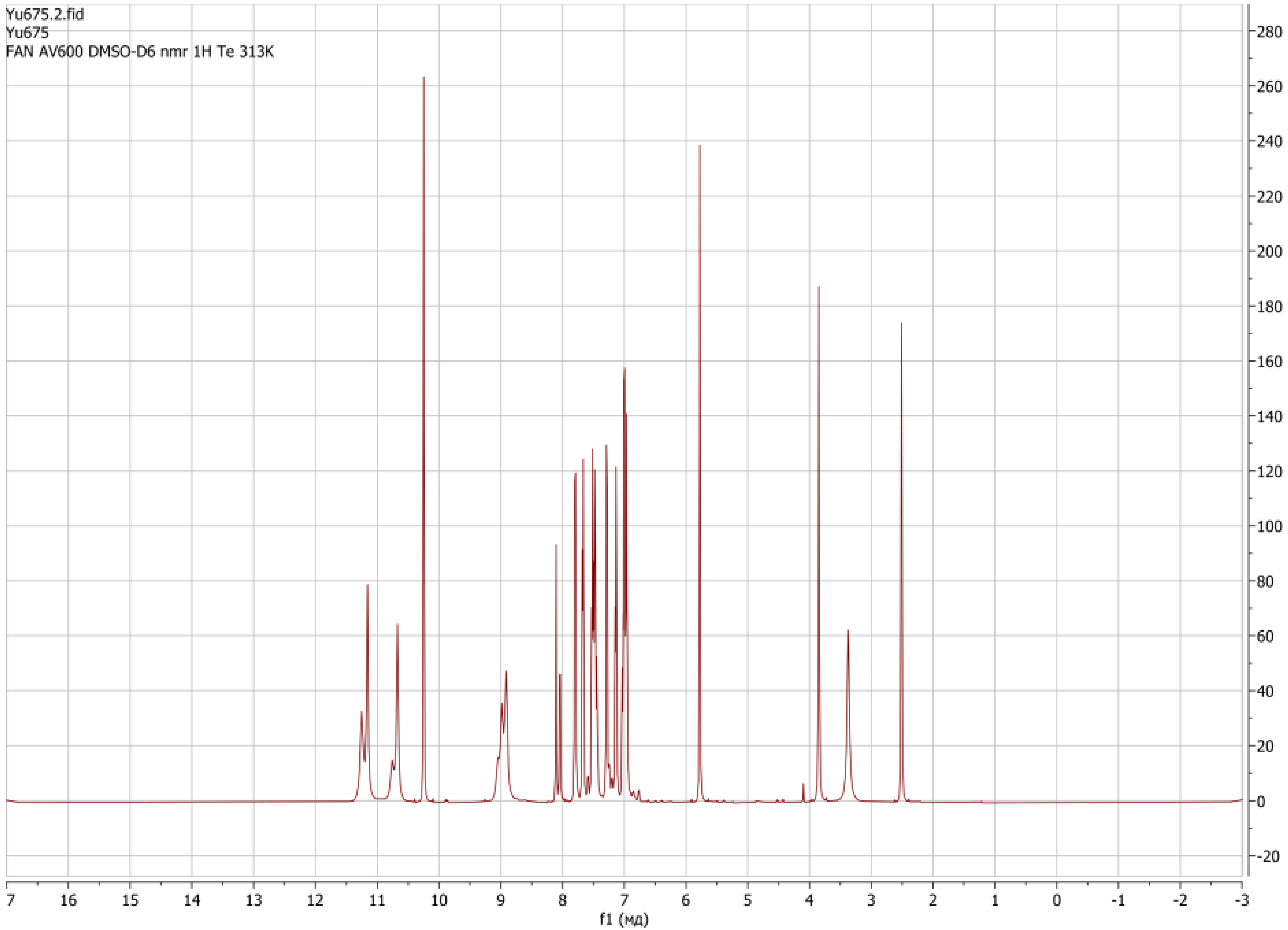
Yu675.2.fid
Yu675
FAN AV600 DMSO-D6 nmr 1H Te 313K
f1 (мд)

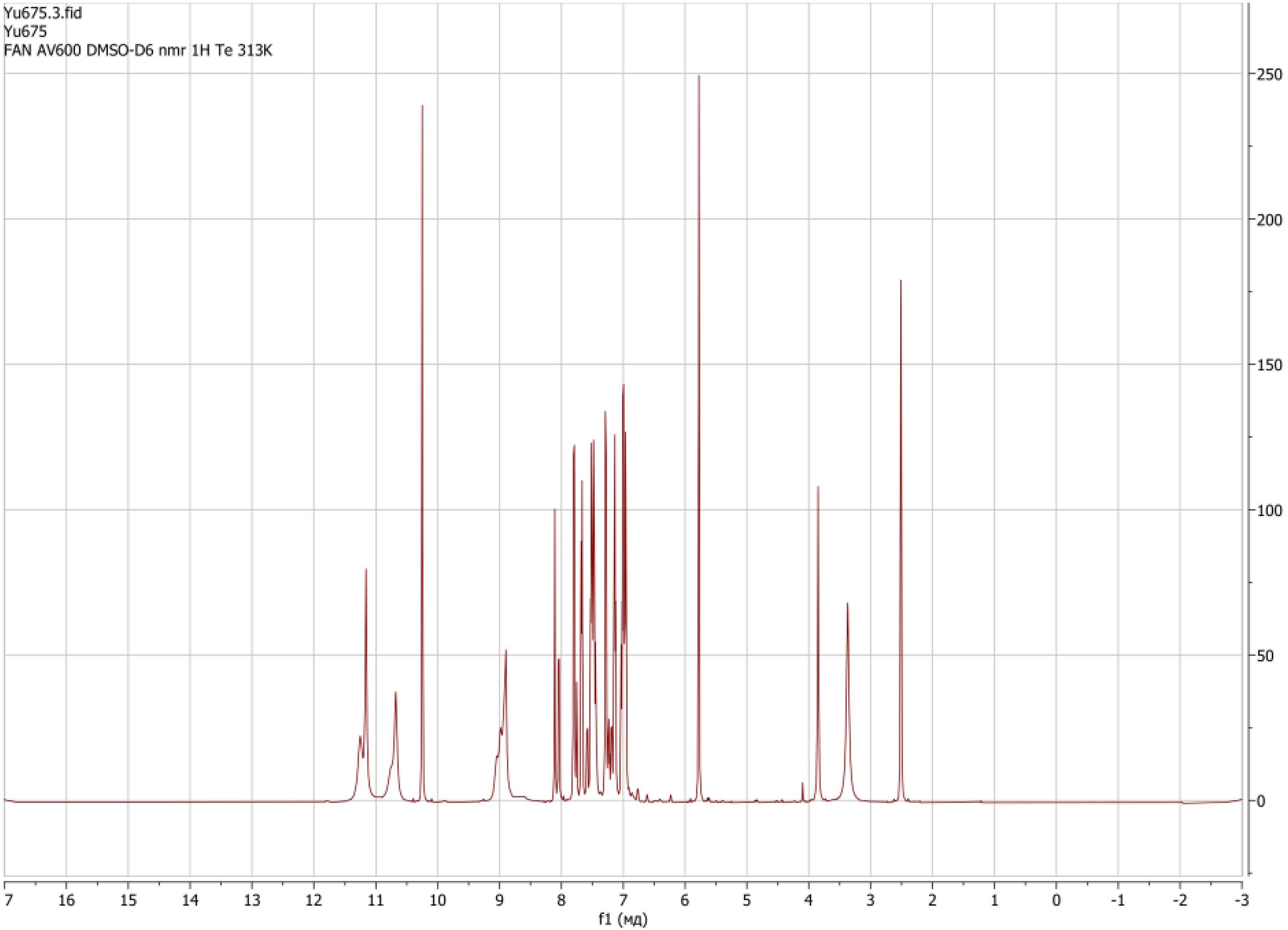
Yu675.3.fid
Yu675
FAN AV600 DMSO-D6 nmr 1H Te 313K
250
200
150
100
50
0
7
16
15
14
13
12
11
10
9
8
7
6
5
4
3
2
1
0
-1
-2
-3
f1 (мд)

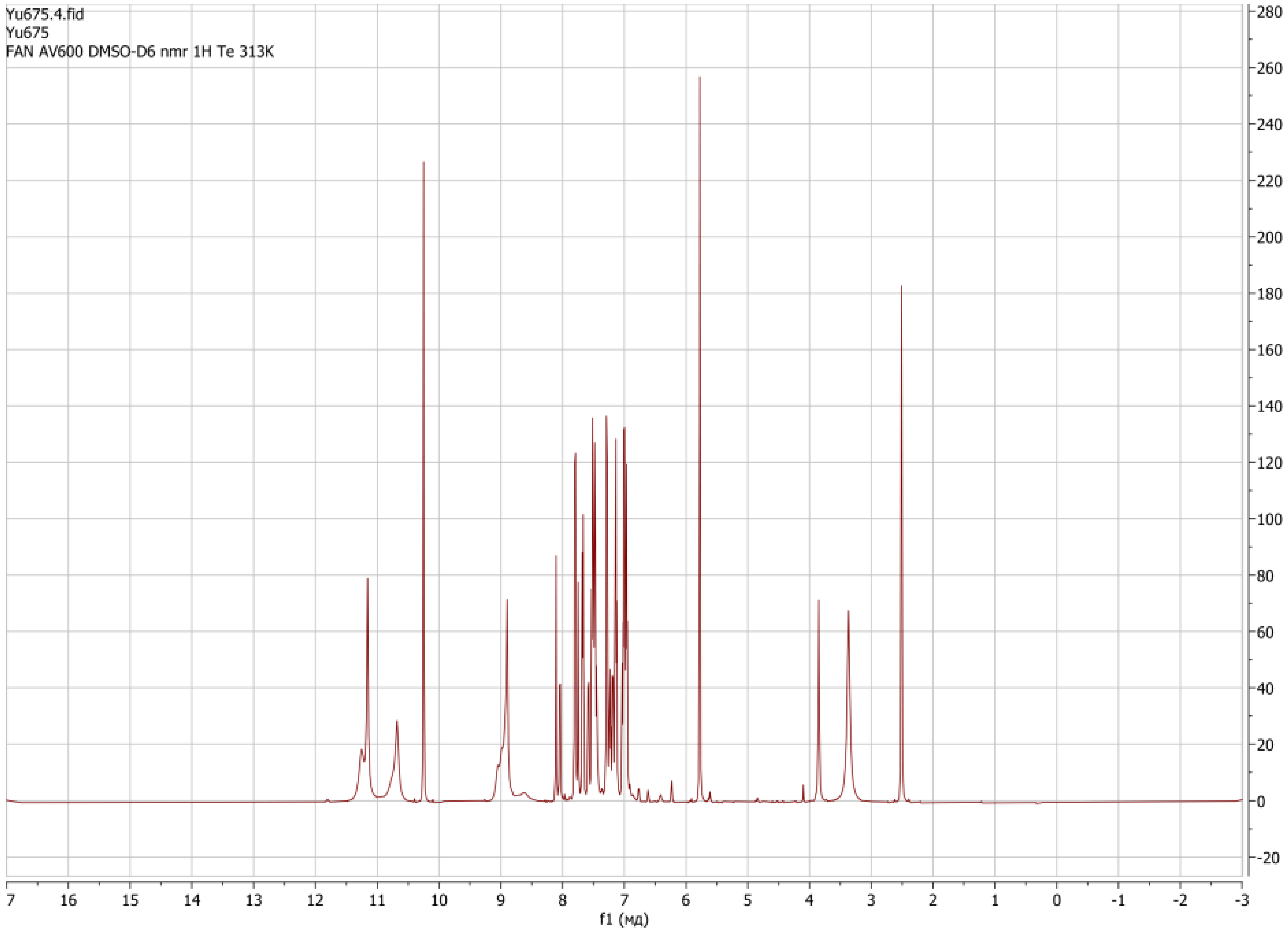

Yu675.4.fid
Yu675
FAN AV600 DMSO-D6 nmr 1H Te 313K
f1 (мд)

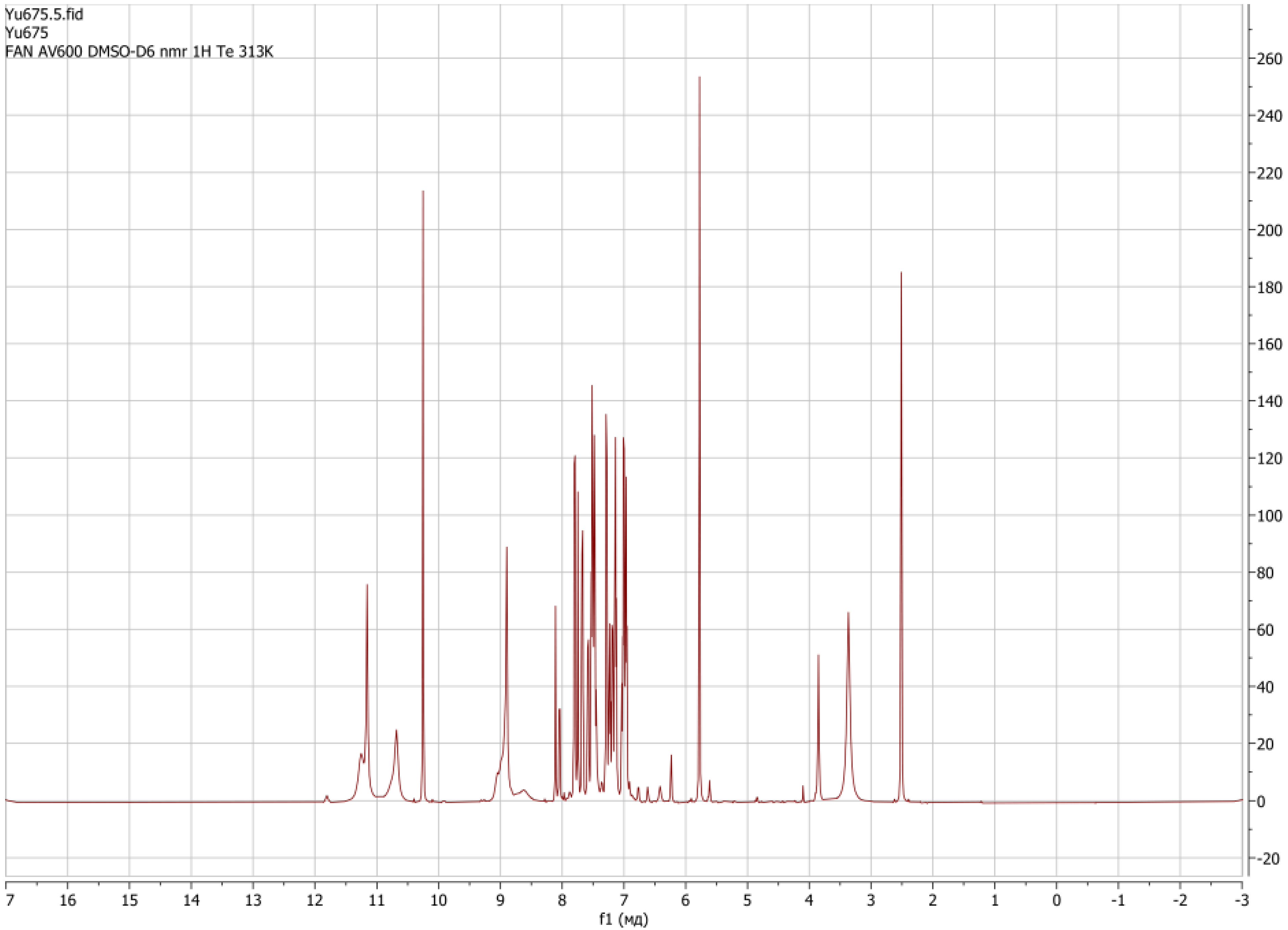

Yu675.5.fid
Yu675
FAN AV600 DMSO-D6 nmr 1H Te 313K
f1 (мд)

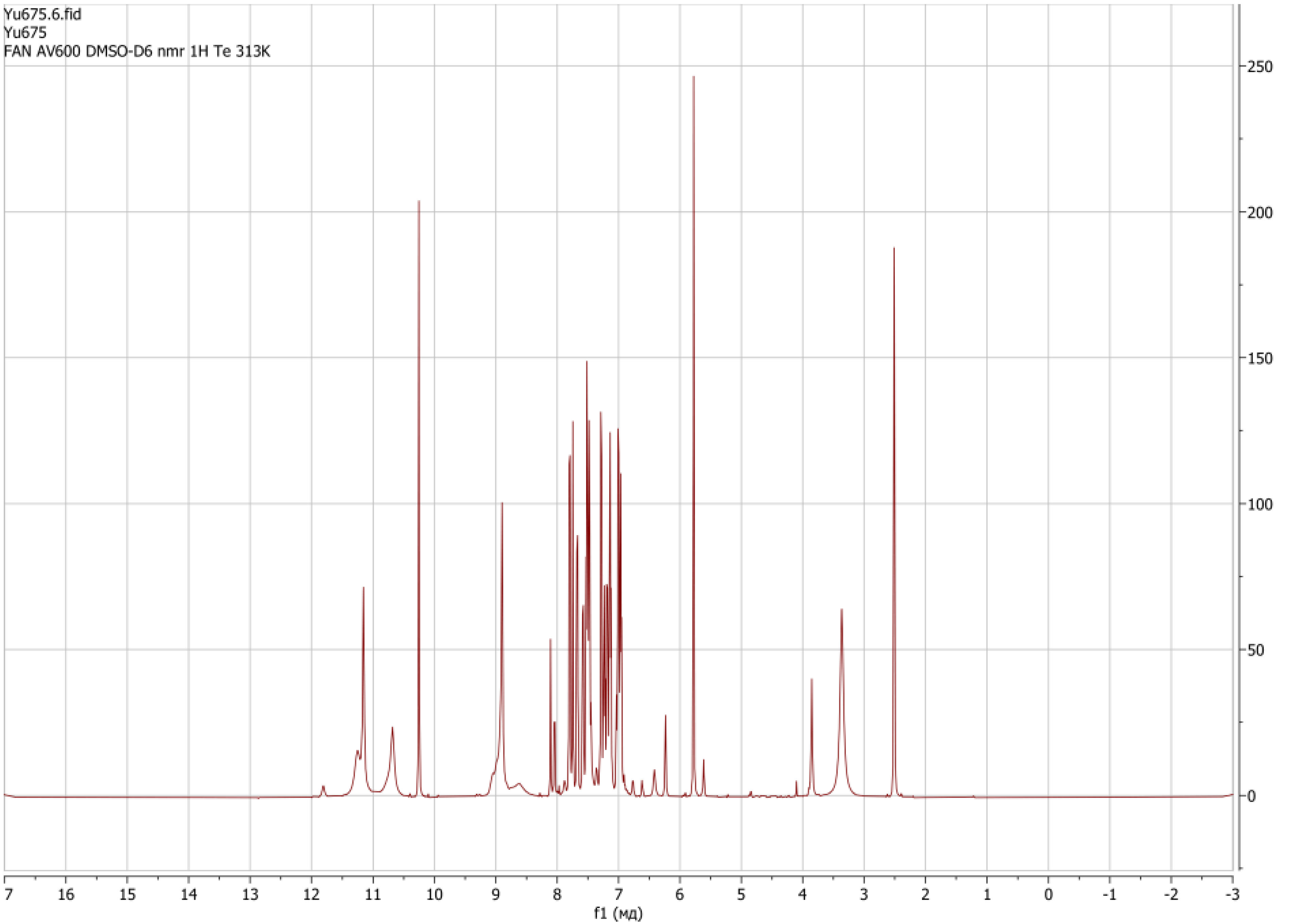
Yu675.6.fid
Yu675
FAN AV600 DMSO-D6 nmr 1H Te 313K
f1 (мд)

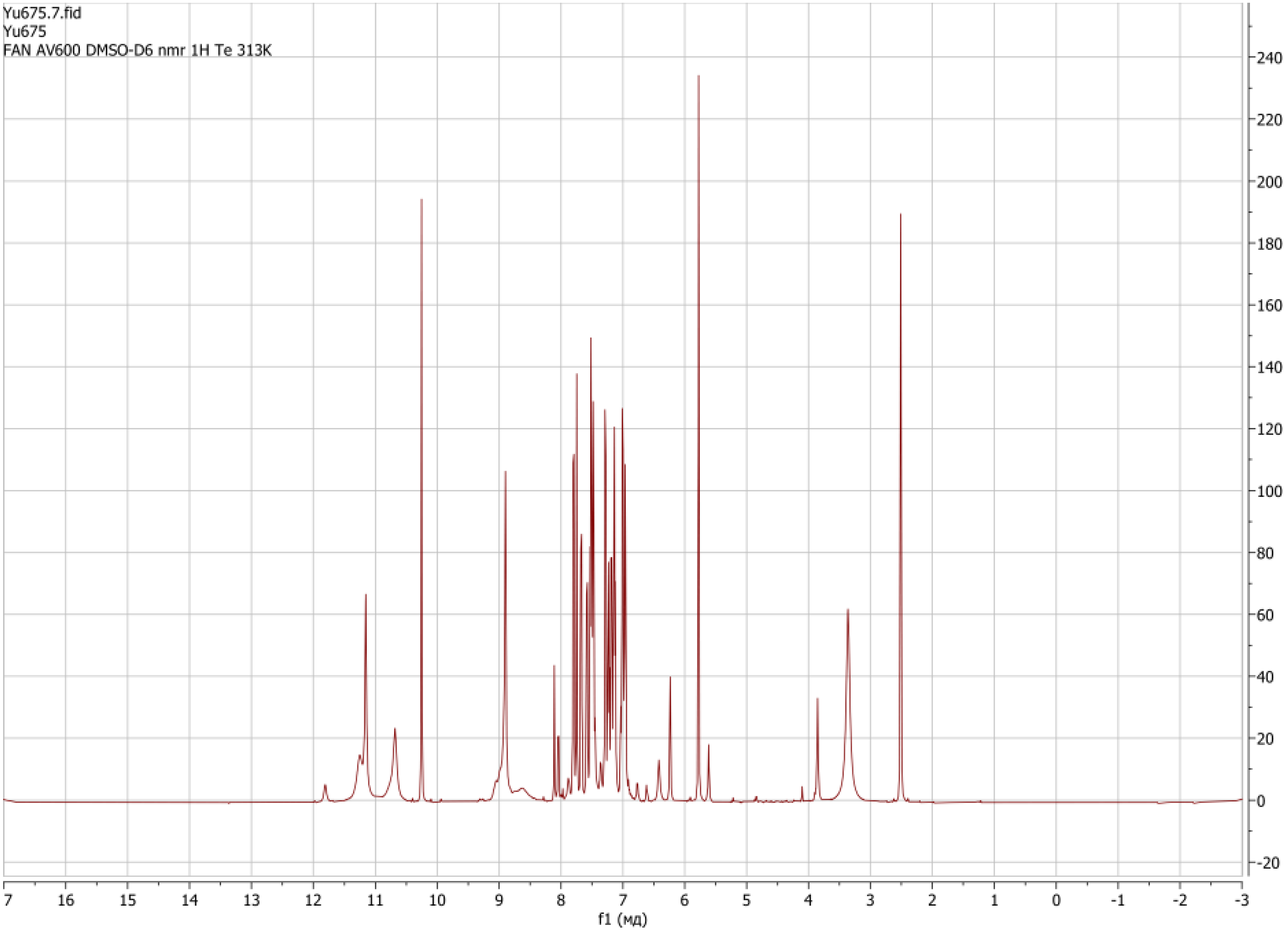

Yu675.7.fid
Yu675
FAN AV600 DMSO-D6 nmr 1H Te 313K
f1 (мд)

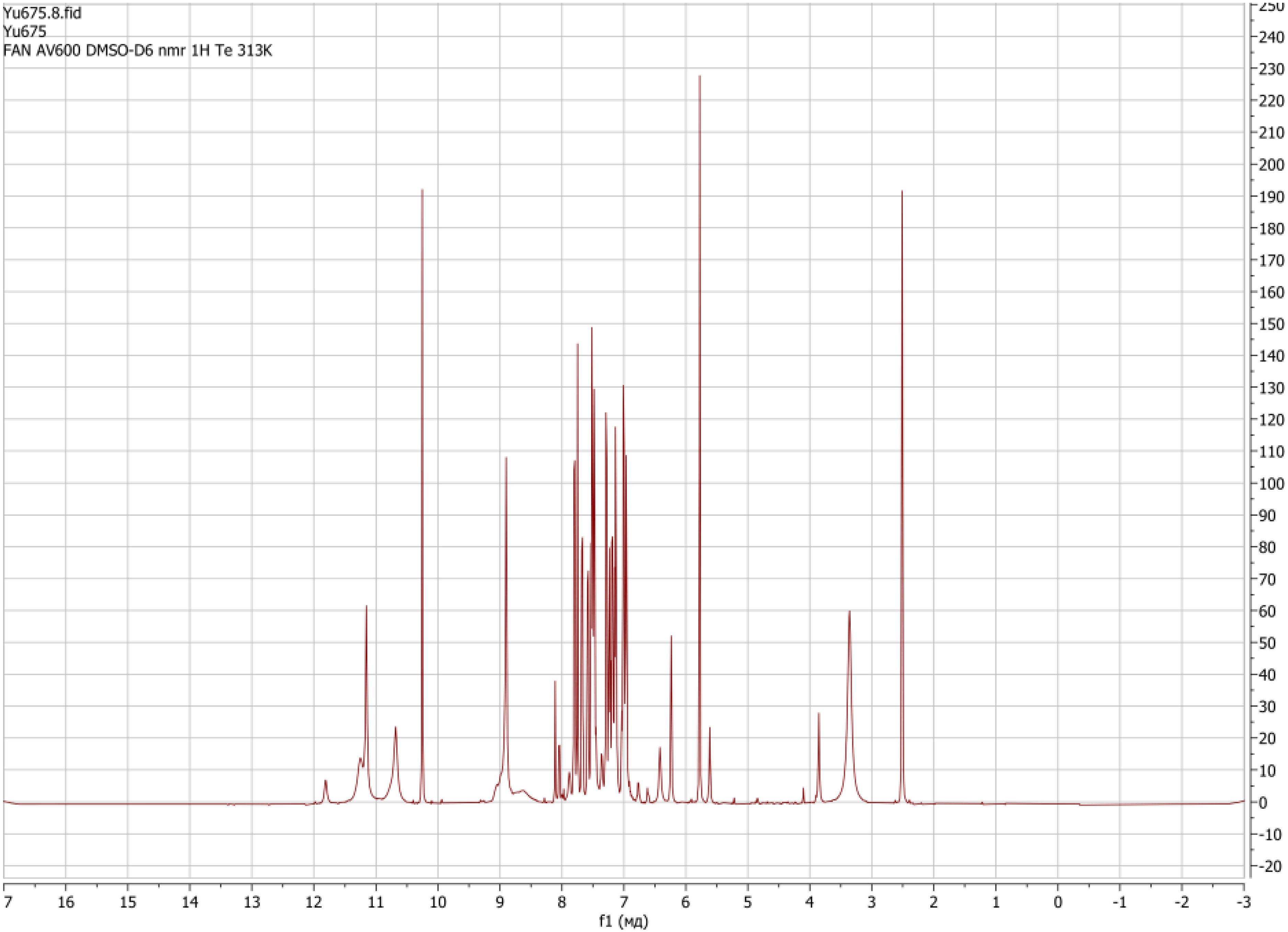

Yu675.8.fid
Yu675
FAN AV600 DMSO-D6 nmr 1H Te 313K
f1 (мд)

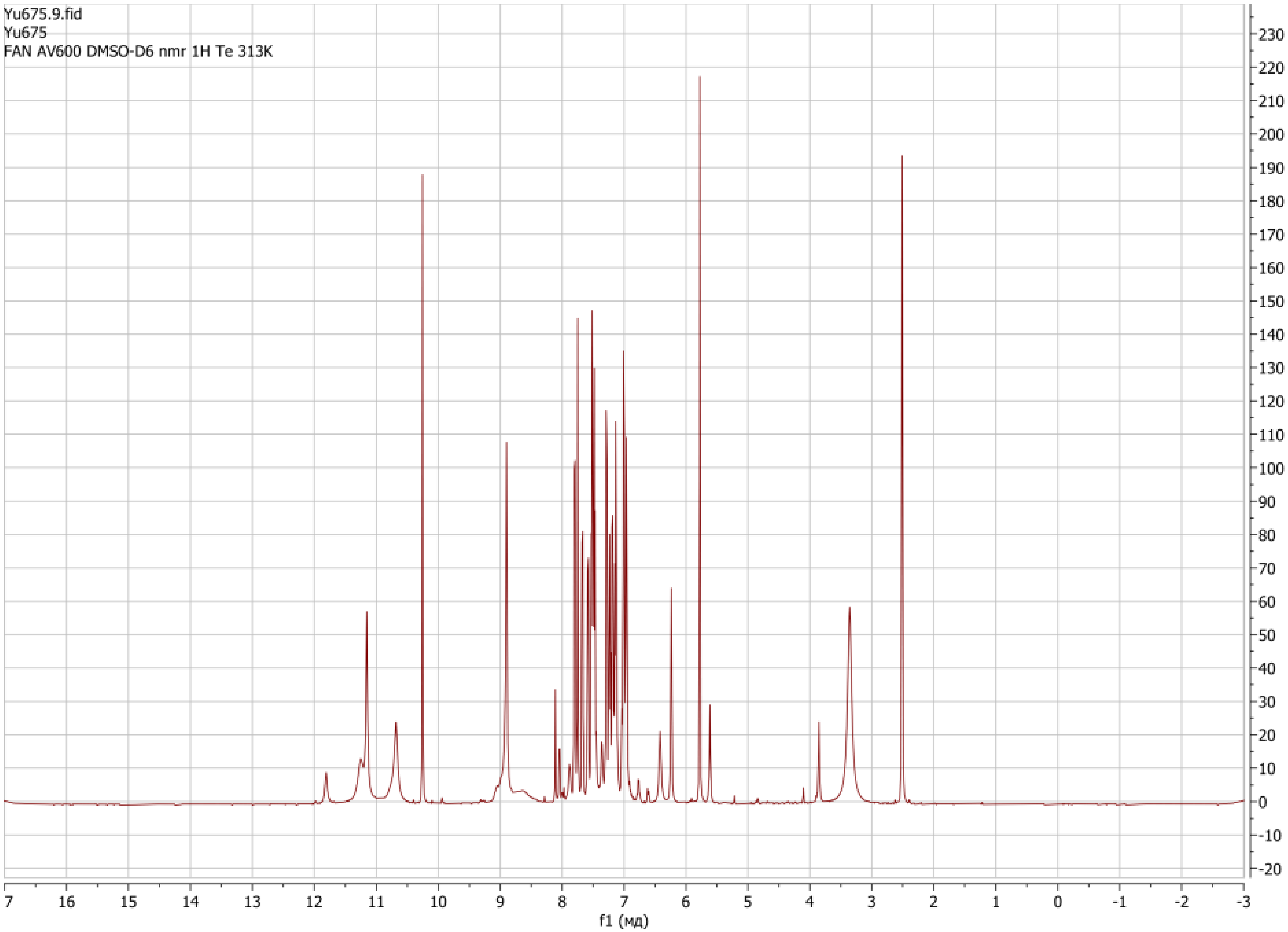
Yu675.9.fid
Yu675
FAN AV600 DMSO-D6 nmr 1H Te 313K
f1 (мд)

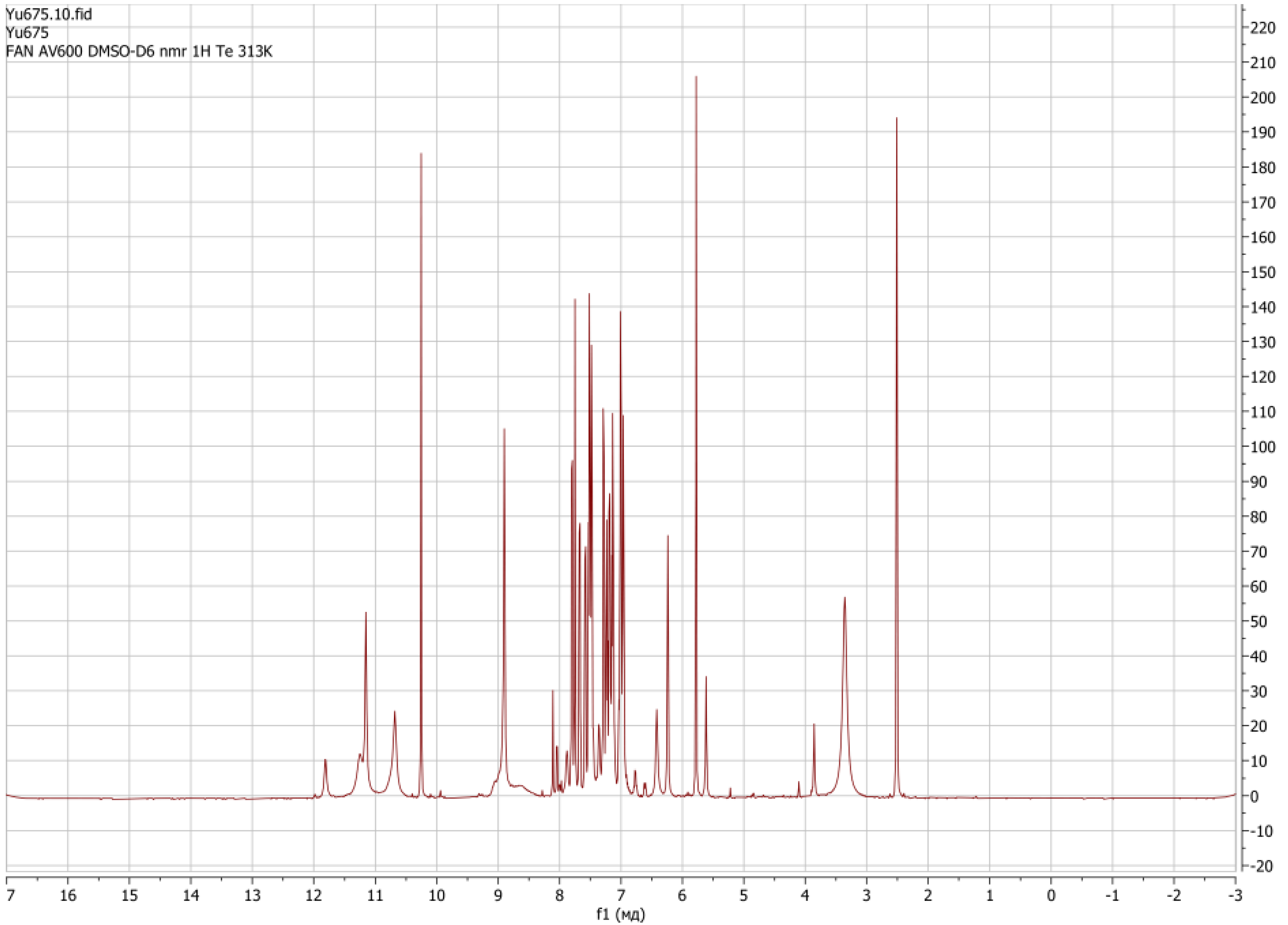
Yu675.10.fid
Yu675
FAN AV600 DMSO-D6 nmr 1H Te 313K
f1 (мд)

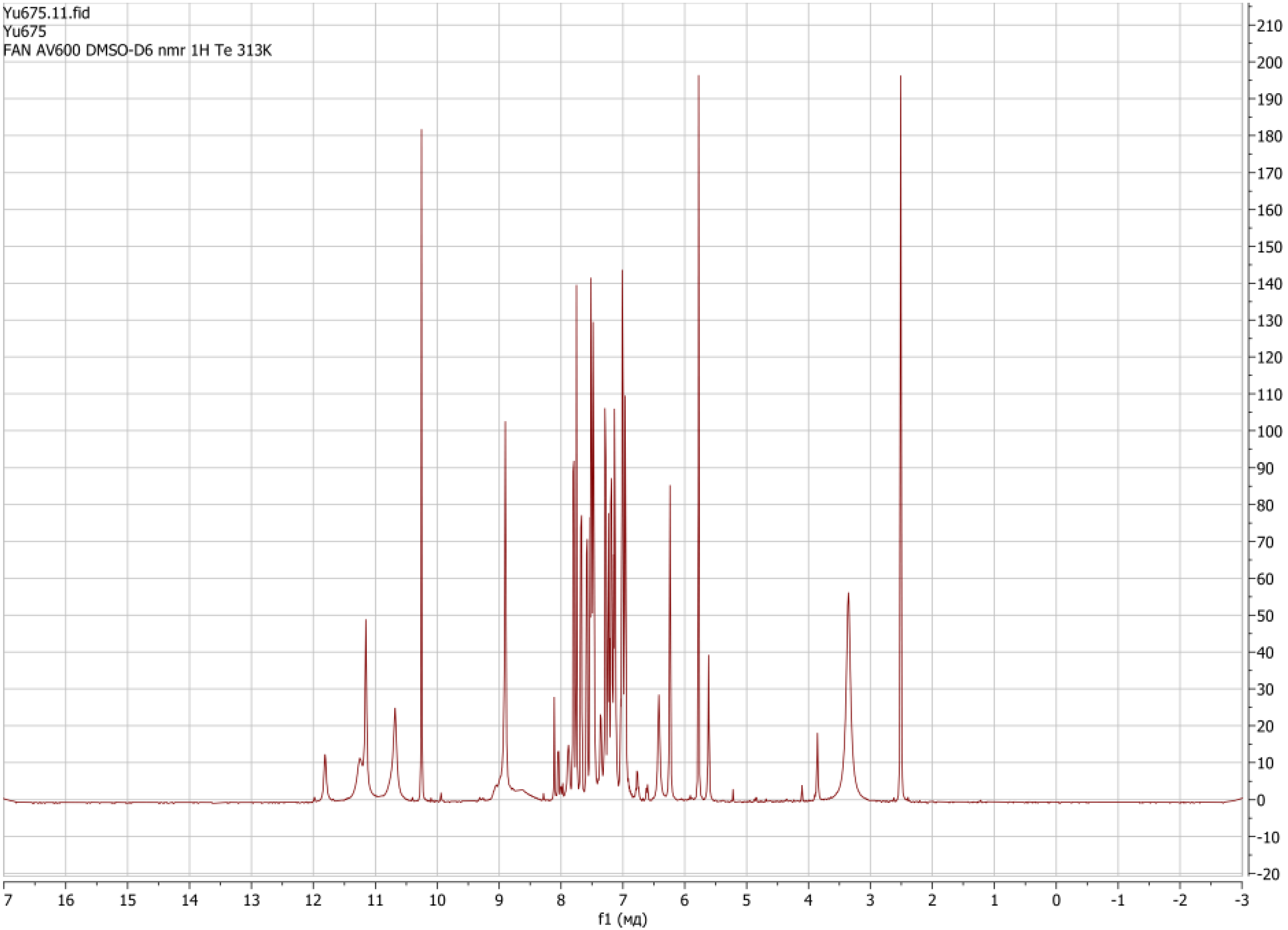
Yu675.11.fid
Yu675
FAN AV600 DMSO-D6 nmr 1H Te 313K
f1 (мд)

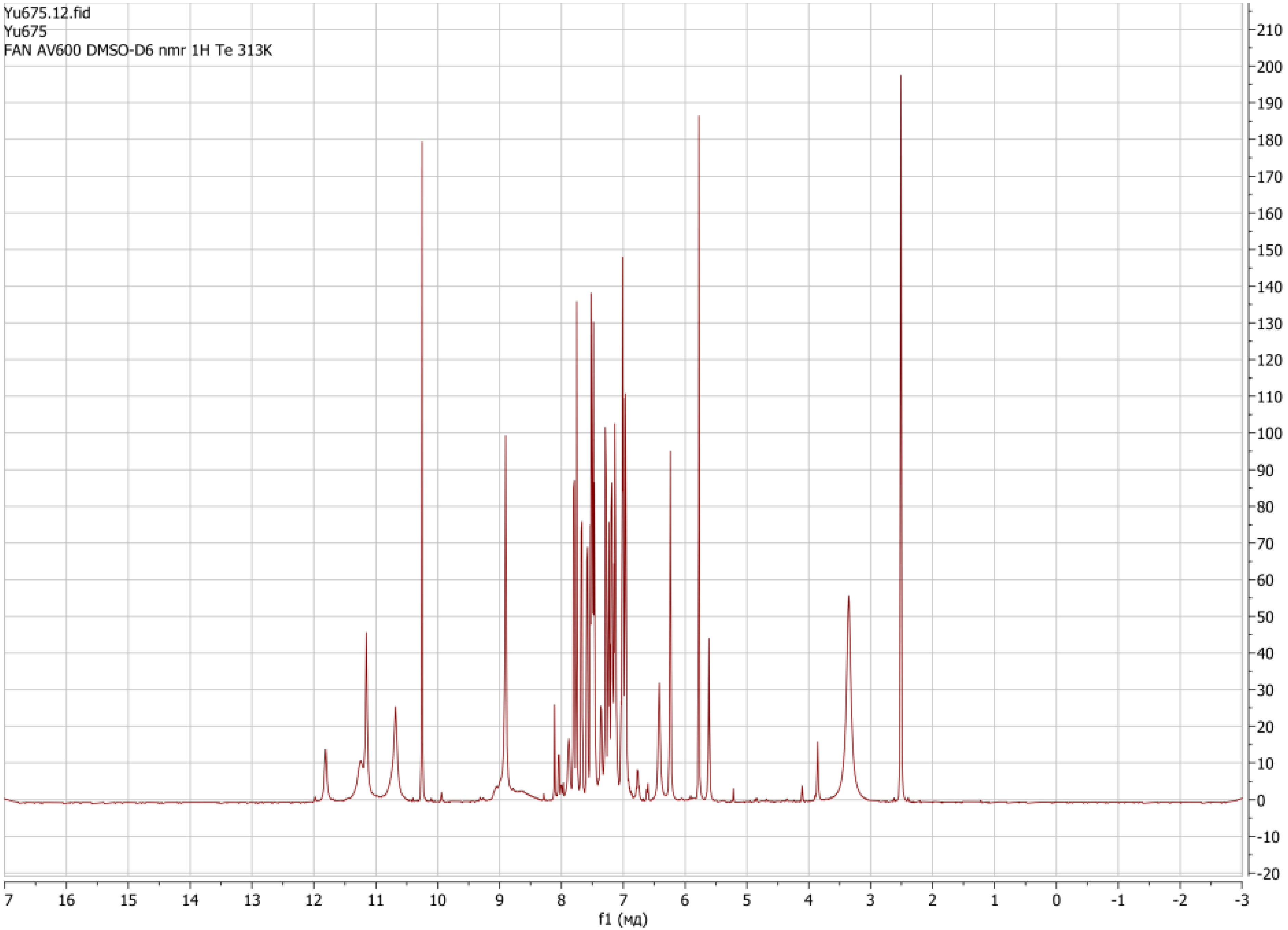
Yu675.12.fid
Yu675
FAN AV600 DMSO-D6 nmr 1H Te 313K
f1 (мд)

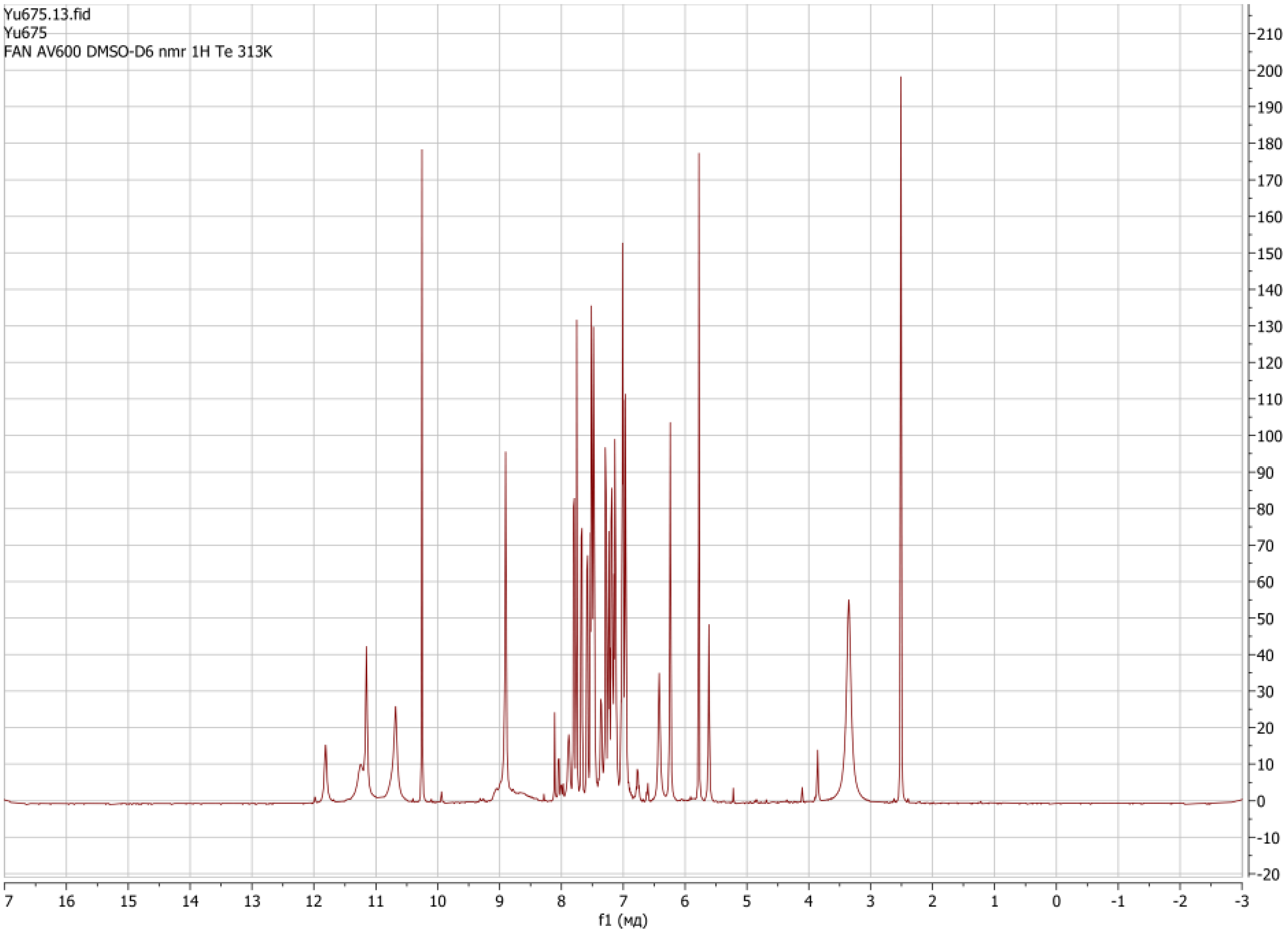

Yu675.13.fid
Yu675
FAN AV600 DMSO-D6 nmr 1H Te 313K
f1 (мд)

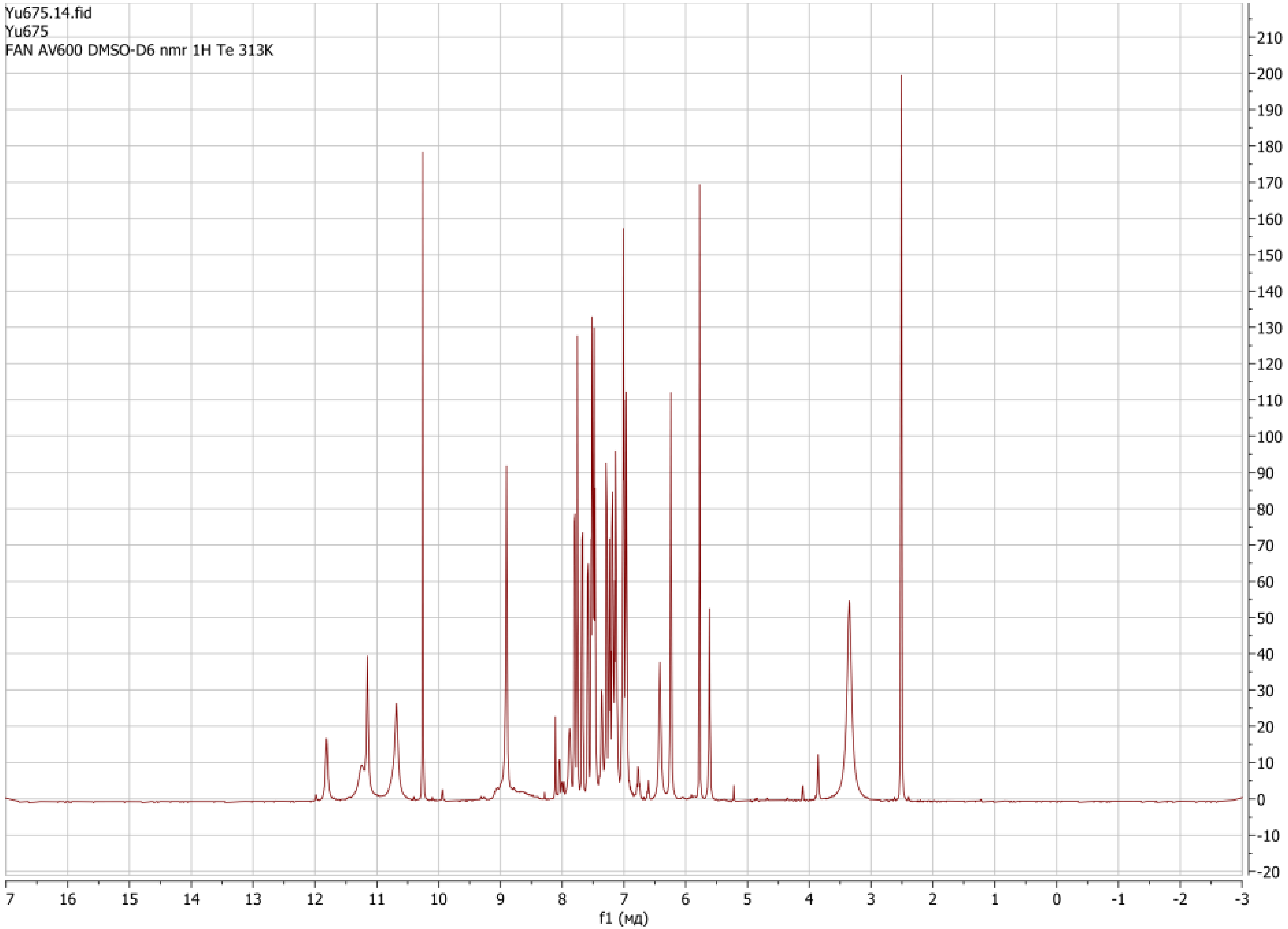
Yu675.14.fid
Yu675
FAN AV600 DMSO-D6 nmr 1H Te 313K
f1 (мд)

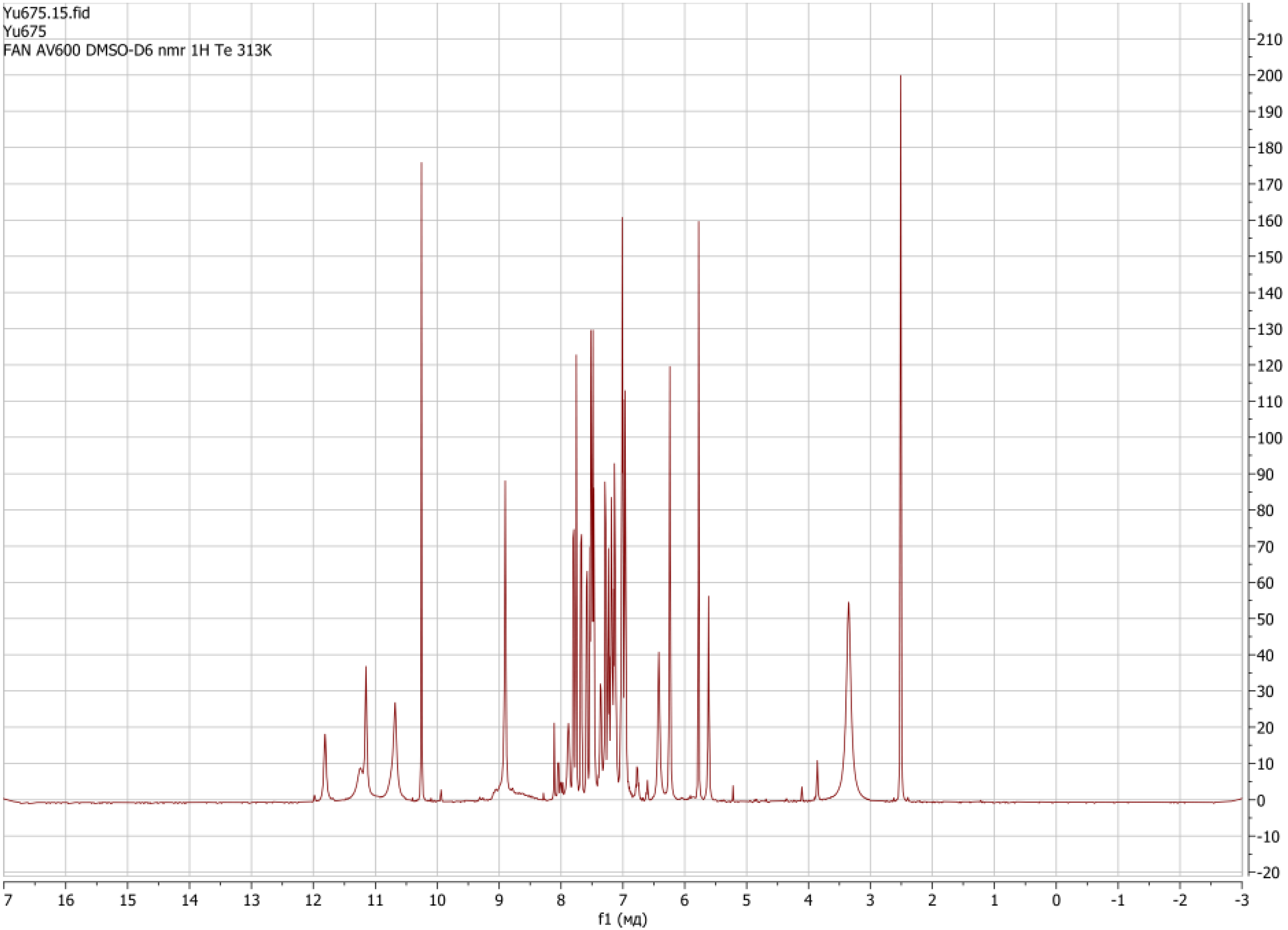

Yu675.15.fid
Yu675
FAN AV600 DMSO-D6 nmr 1H Te 313K
f1 (мд)

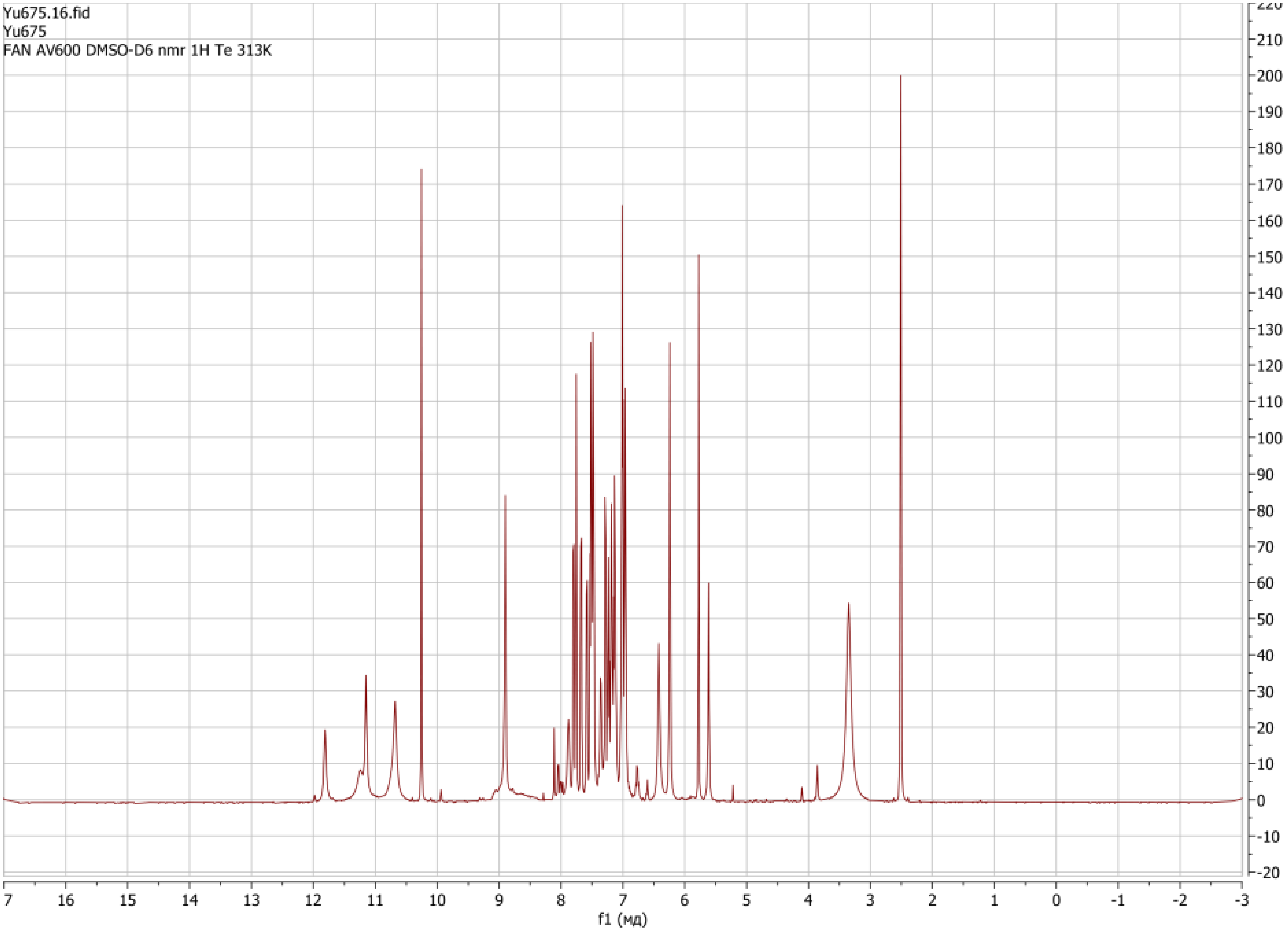

Yu675.16.fid
Yu675
FAN AV600 DMSO-D6 nmr 1H Te 313K
f1 (мд)

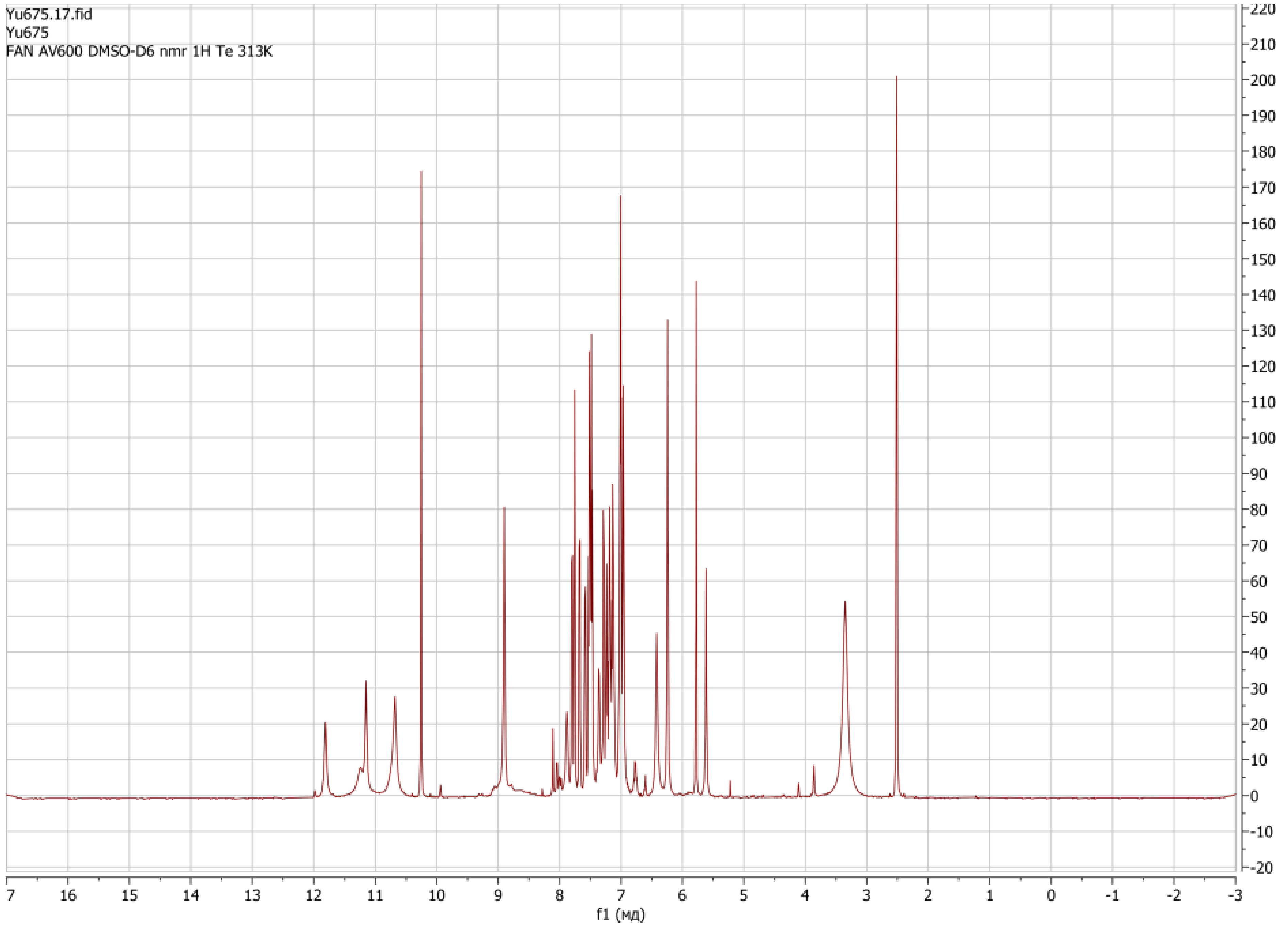
Yu675.17.fid
Yu675
FAN AV600 DMSO-D6 nmr 1H Te 313K
f1 (мд)

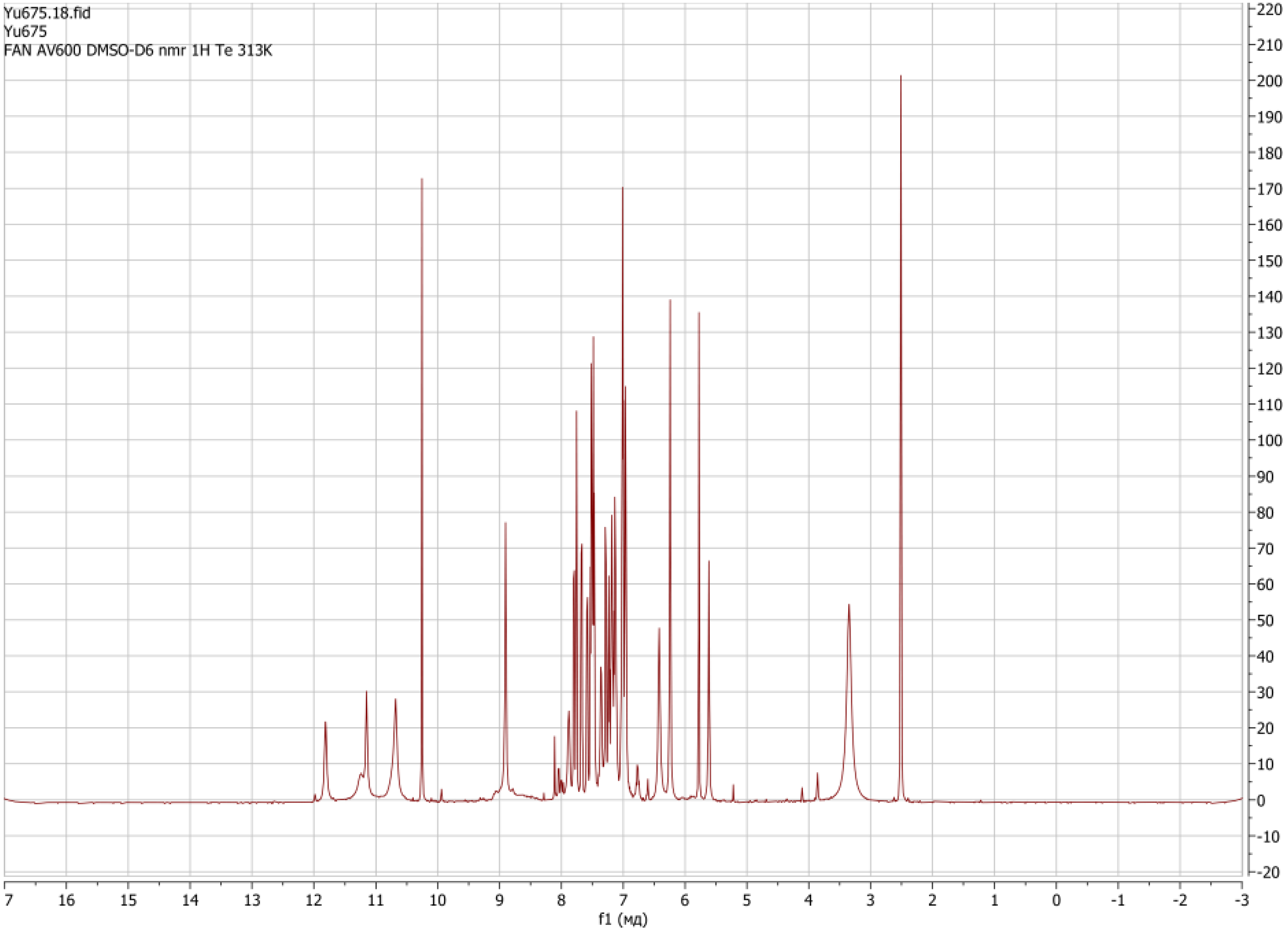
Yu675.18.fid
Yu675
FAN AV600 DMSO-D6 nmr 1H Te 313K
f1 (мд)

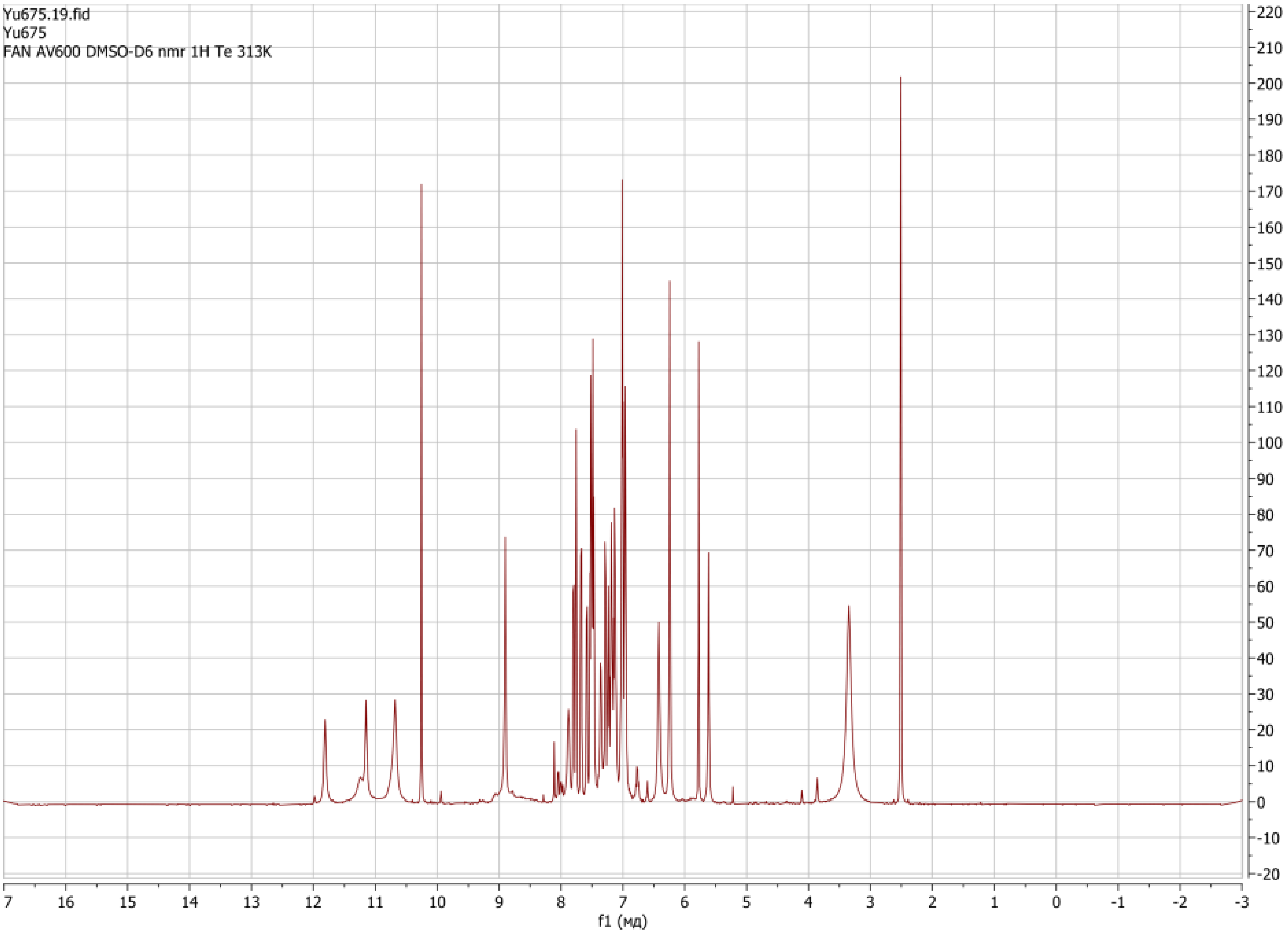

Yu675.19.fid
Yu675
FAN AV600 DMSO-D6 nmr 1H Te 313K
f1 (мд)

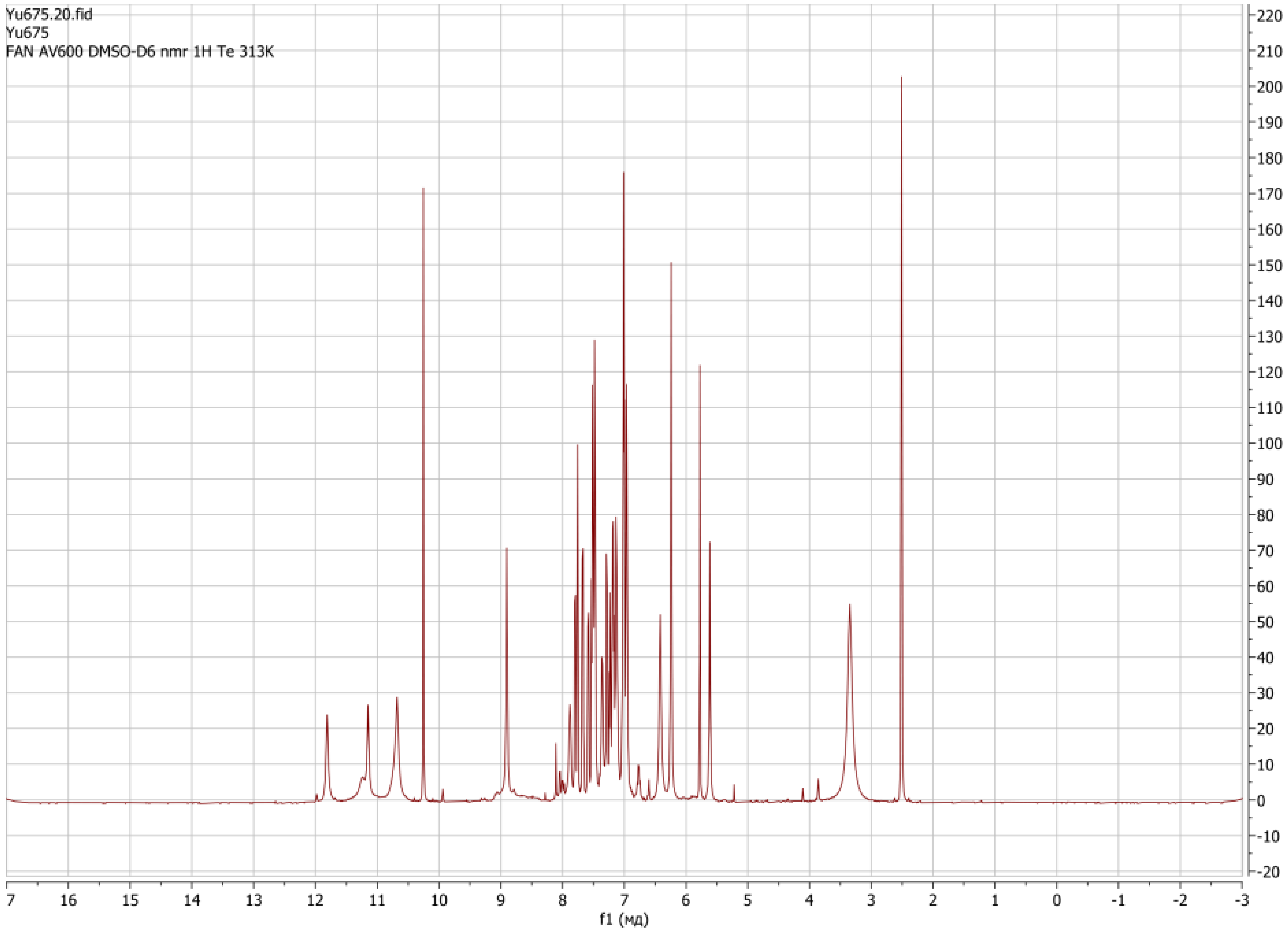
Yu675.20.fid
Yu675
FAN AV600 DMSO-D6 nmr 1H Te 313K
f1 (мд)

Quantum chemistry simulations

Results of quantum chemical calculations for studied compounds **4a-i** with extended decimal point are presented in Table S1

Table S1. Results of quantum chemical calculations for studied compounds **4a-i** (extended decimal point)

Comp.	**4a**	**4b**	**4c**	**4d**	**4e**	**4f**	**4g**	**4h**	**4i**
					Gas phase calculations				
Total energy, a.u.	-1347.192591	-1386.519626	-1461.746133	-1501.069826	-4035.282019	-1806.813879	-3920.734590	-1551.750784	-1500.868189
$E_{(HOMO)}$, eV	-5.707	-5.649	-5.644	-5.558	-5.695	-5.835	-5.830	-6.065	-5.669
$E_{(LUMO)}$, eV	-2.040	-2.023	-1.997	-1.982	-2.072	-2.135	-2.131	-2.416	-2.047
$\Delta E_{(L-H)}$, eV	3.667	3.626	3.647	3.576	3.623	3.700	3.699	3.649	3.622
μ, D	7.896	8.071	7.645	8.119	7.487	7.325	7.351	7.619	7.670
χ	3.874	3.836	3.821	3.770	3.884	3.986	3.981	4.241	3.858
η	1.834	1.813	1.824	1.788	1.812	1.85	1.849	1.825	1.811
ω	4.092	4.058	4.003	3.975	4.164	4.301	4.286	4.929	4.109
σ	0.545	0.552	0.548	0.559	0.552	0.541	0.541	0.548	0.552
					Calculations for solvated compounds				
Total energy, a.u.	-1347.218268	-1386.545401	-1461.773407	-1501.098635	-4035.310729	-1806.839494	-3920.760255	-1551.779693	-1500.894522
$E_{(HOMO)}$, eV	-5.993	-5.947	-5.940	-5.900	-5.968	-6.037	-6.035	-6.139	-5.934
$E_{(LUMO)}$, eV	-1.795	-1.791	-1.783	-1.791	-1.822	-1.826	-1.825	-2.726	-1.810
$\Delta E_{(L-H)}$, eV	4.198	4.156	4.157	4.109	4.146	4.211	4.210	3.413	4.124
μ, D	10.263	11.789	9.883	10.873	11.137	9.490	9.486	9.609	10.135
χ	3.894	3.869	3.867	3.846	3.895	3.932	3.930	4.433	3.872
η	2.099	2.078	2.079	2.055	2.073	2.106	2.105	1.707	2.062
ω	3.612	3.602	3.596	3.599	3.659	3.671	3.669	5.756	3.635
σ	0.476	0.481	0.481	0.487	0.482	0.475	0.475	0.586	0.485
					Calculations for protonated forms of studied compounds				
Total energy, a.u.	-1347.665214	-1386.992850	-1462.220470	-1501.546164	-4035.756965	-1807.285181	-3921.205929	-1552.223091	-1501.341028
$E_{(HOMO)}$, eV	-6.592	-6.541	-6.374	-6.350	-6.449	-6.623	-6.617	-6.670	-6.321
$E_{(LUMO)}$, eV	-1.983	-1.971	-1.967	-1.977	-2.026	-2.035	-2.033	-2.927	-2.044
$\Delta E_{(L-H)}$, eV	4.609	4.57	4.407	4.373	4.423	4.588	4.584	3.743	4.277
μ, D	9.704	9.785	11.099	8.722	13.356	12.543	13.784	16.319	11.489
χ	4.288	4.256	4.171	4.164	4.263	4.329	4.325	4.799	4.183
η	2.305	2.285	2.204	2.187	2.212	2.294	2.292	1.872	2.139
ω	3.988	3.964	3.947	3.964	4.108	4.085	4.081	6.151	4.090
σ	0.434	0.438	0.454	0.457	0.452	0.436	0.436	0.534	0.468

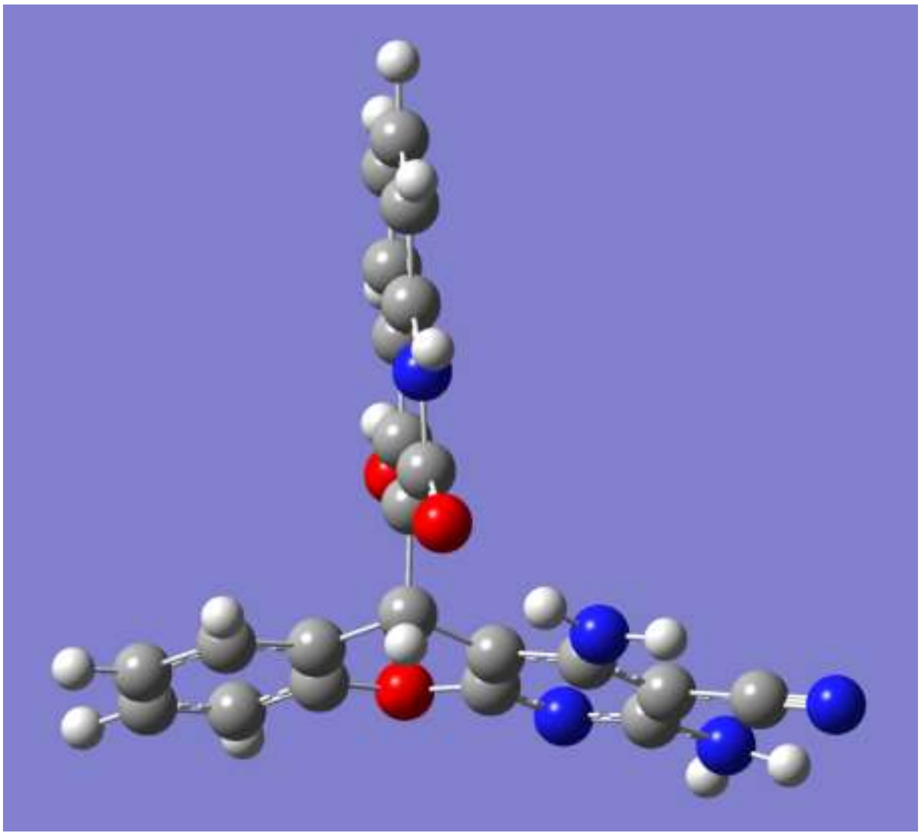

Figure S43. Optimized structure for compound **4a** (gas phase)

Frontier orbitals of several studied compounds

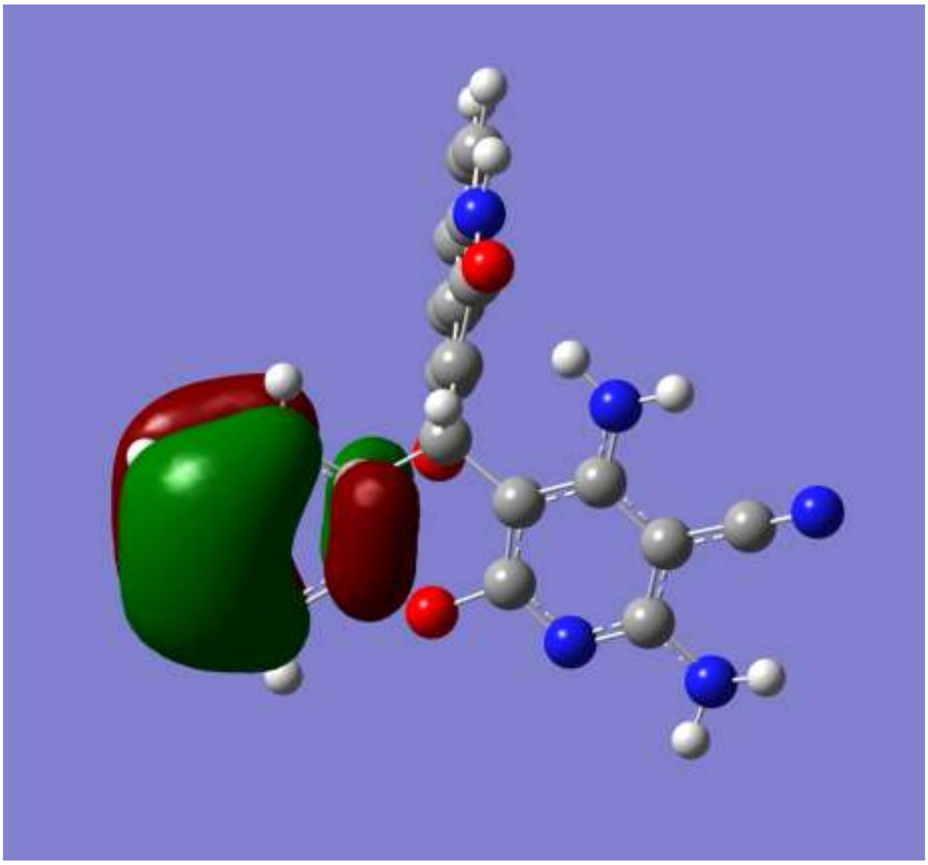

Figure S44. HOMO of **4a** (gas phase)

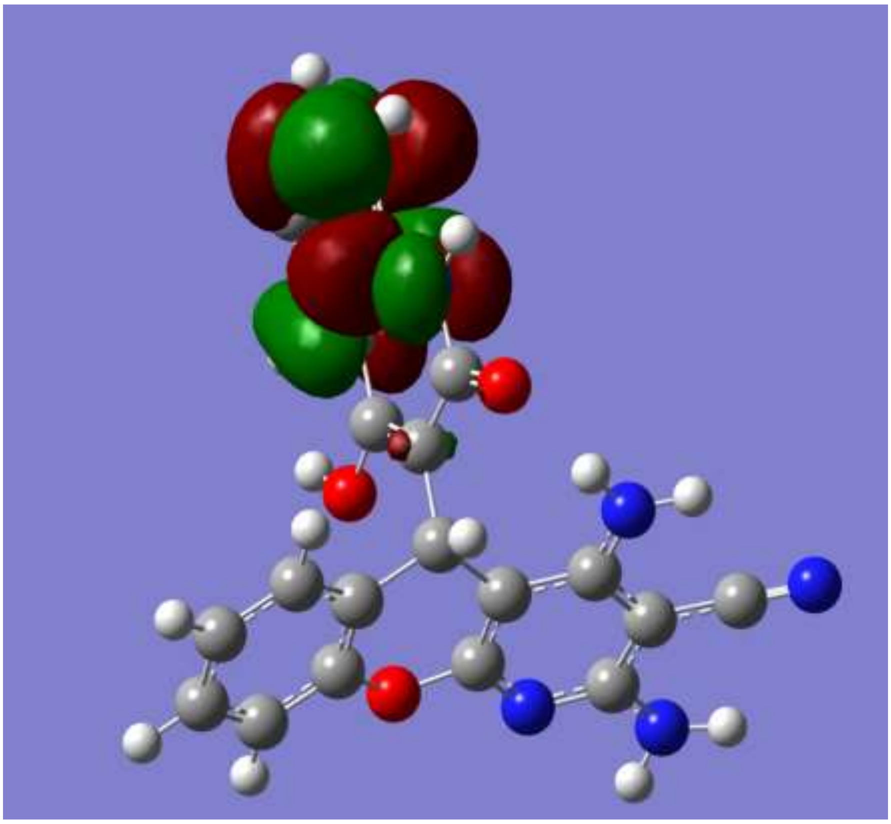

Figure S45. LUMO of **4a** (gas phase)

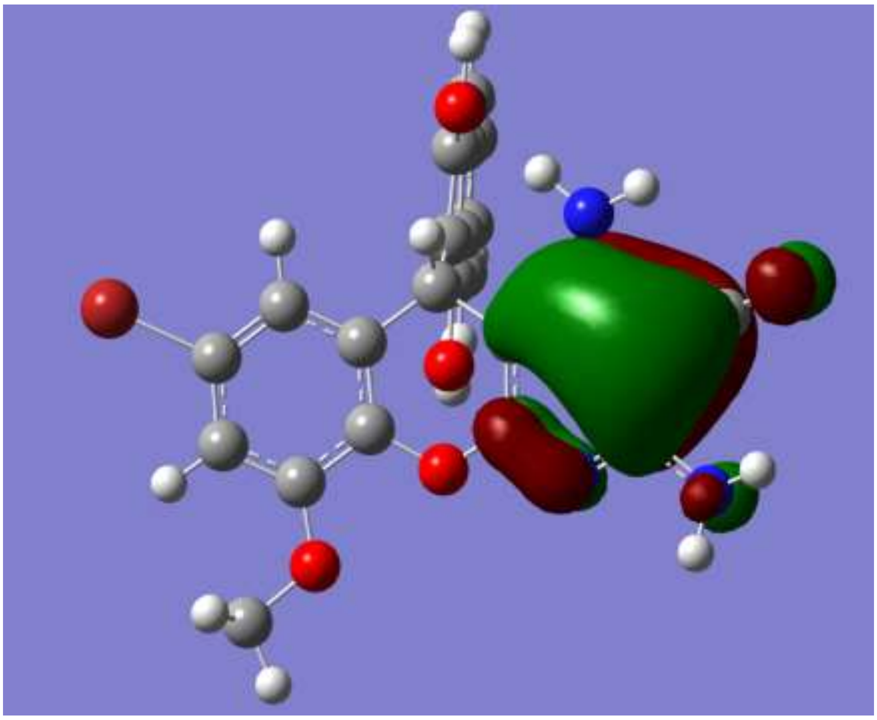

Figure S46. HOMO of **4e** (gas phase)

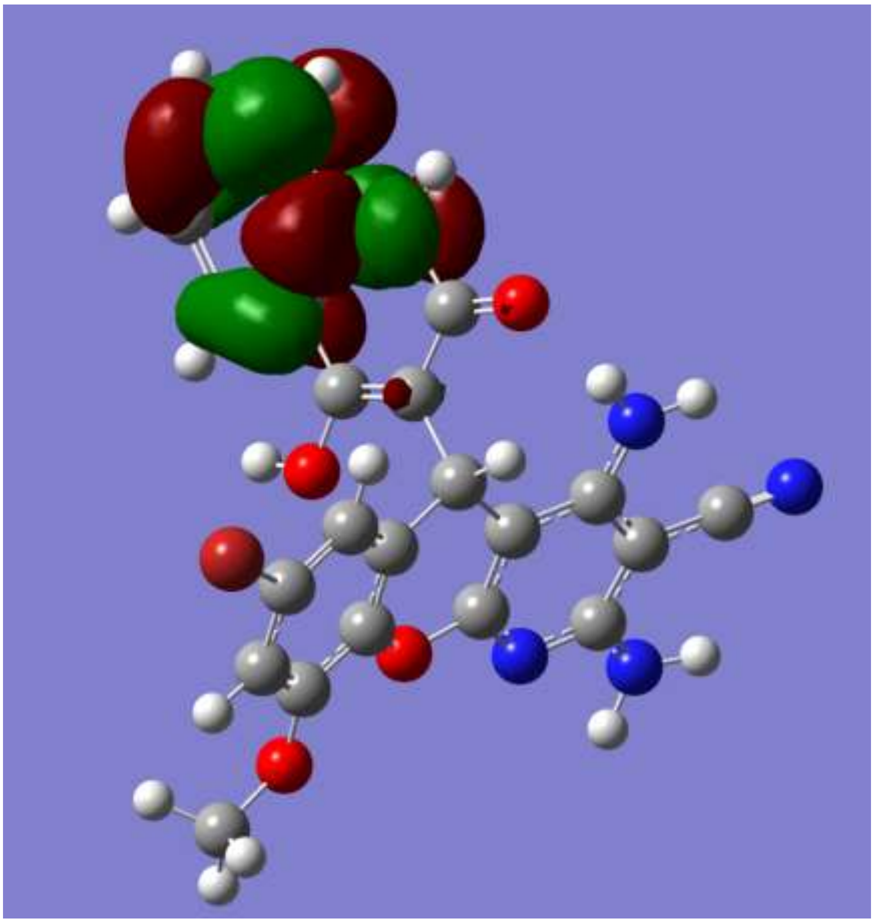

Figure S47. LUMO of **4e** (gas phase)

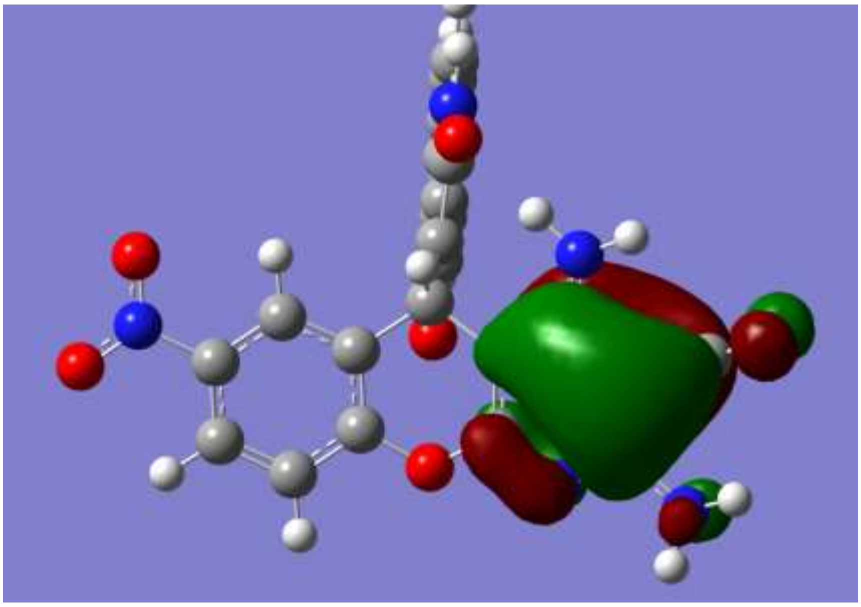

Figure S48. HOMO of **4h** (gas phase)

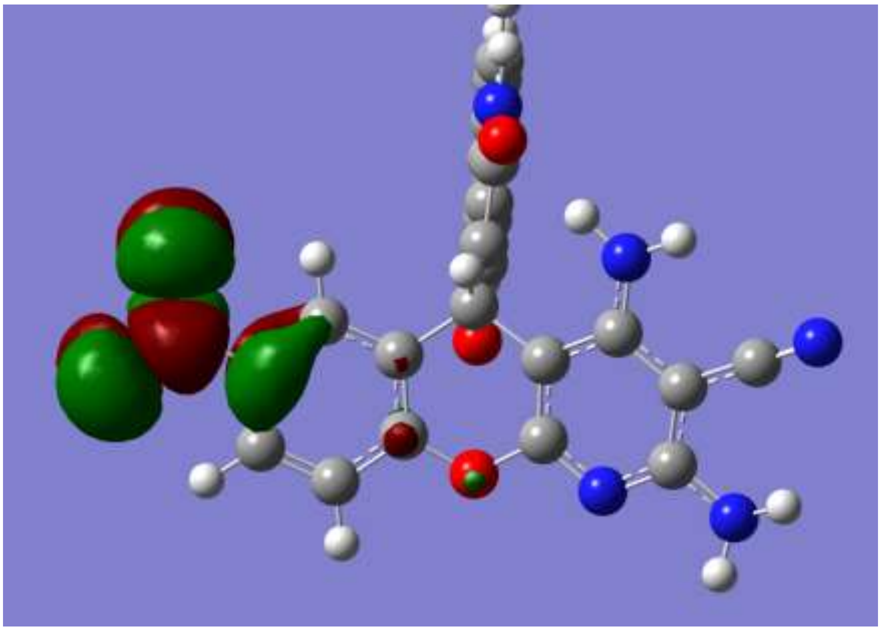

Figure S49. LUMO of **4h** (gas phase)

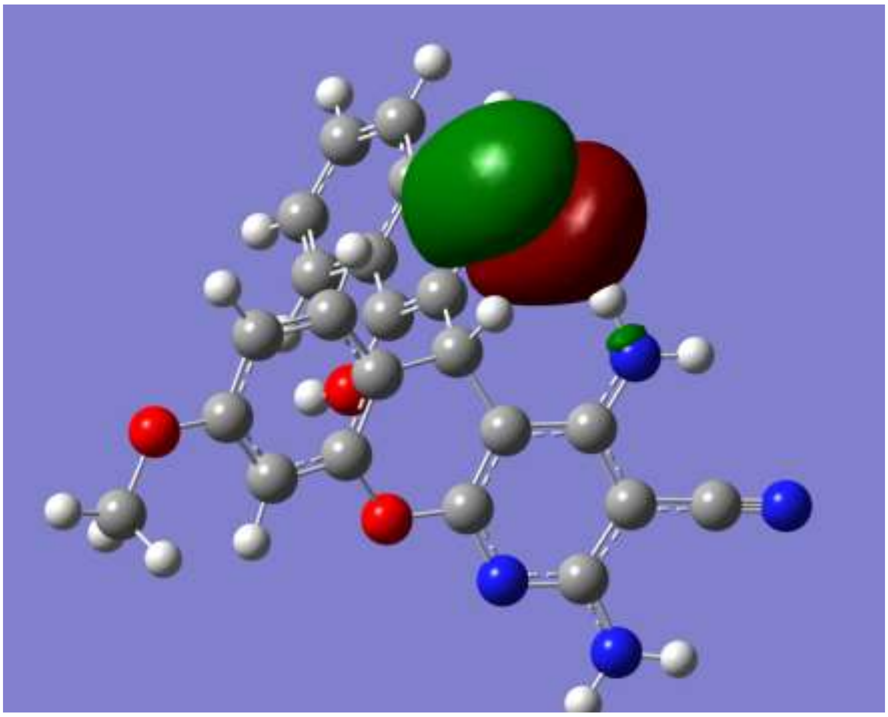

Figure S50. HOMO of **4c** (solvated compound)

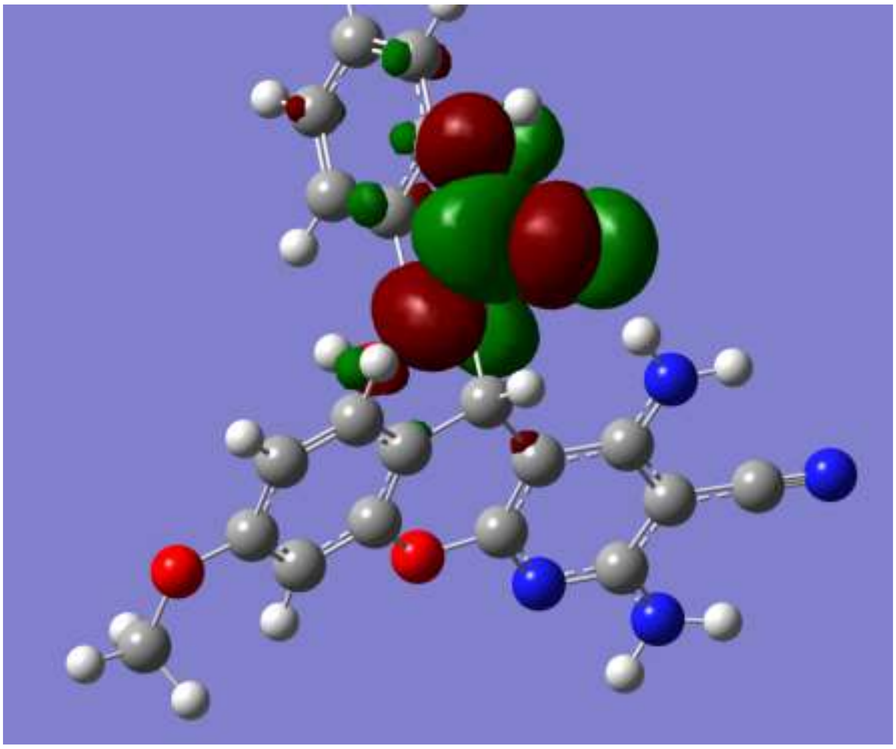

Figure S51. LUMO of **4c** (solvated compound)

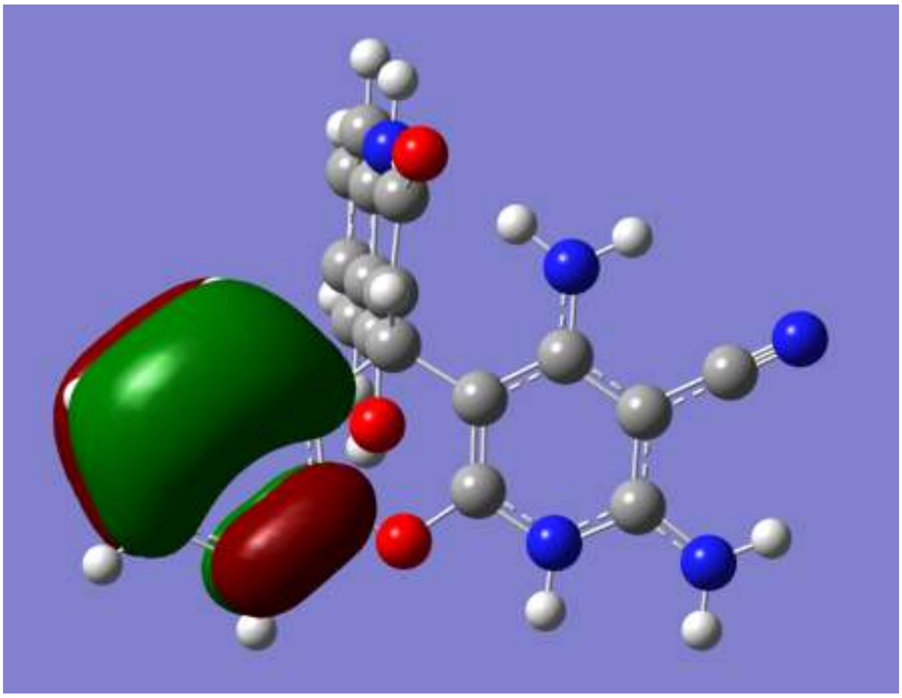

Figure S52. HOMO of **4a** (protonated compound)

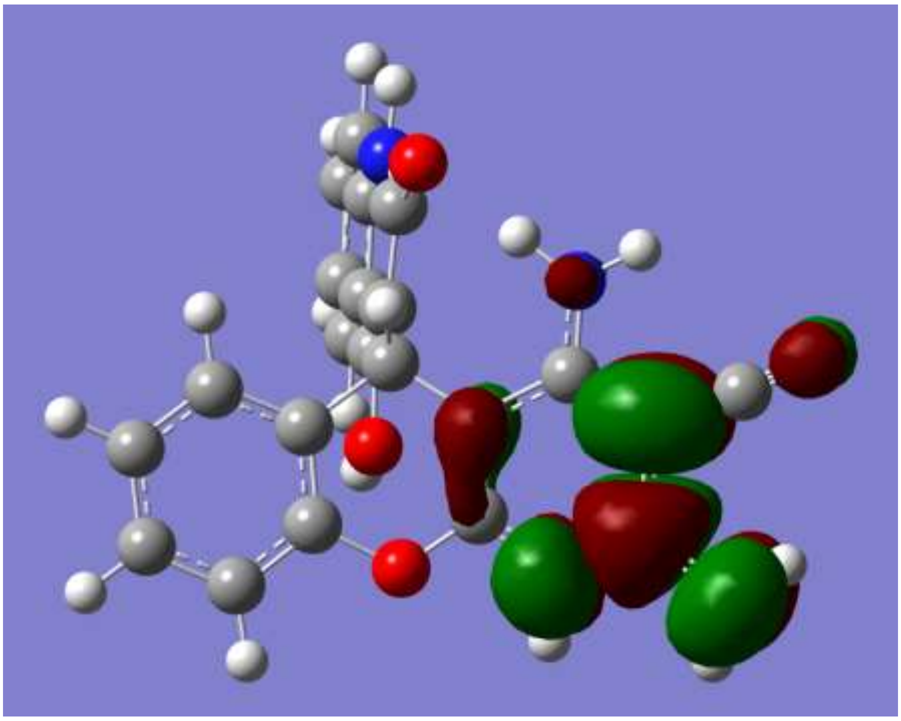

Figure S53. LUMO of **4a** (protonated compound)

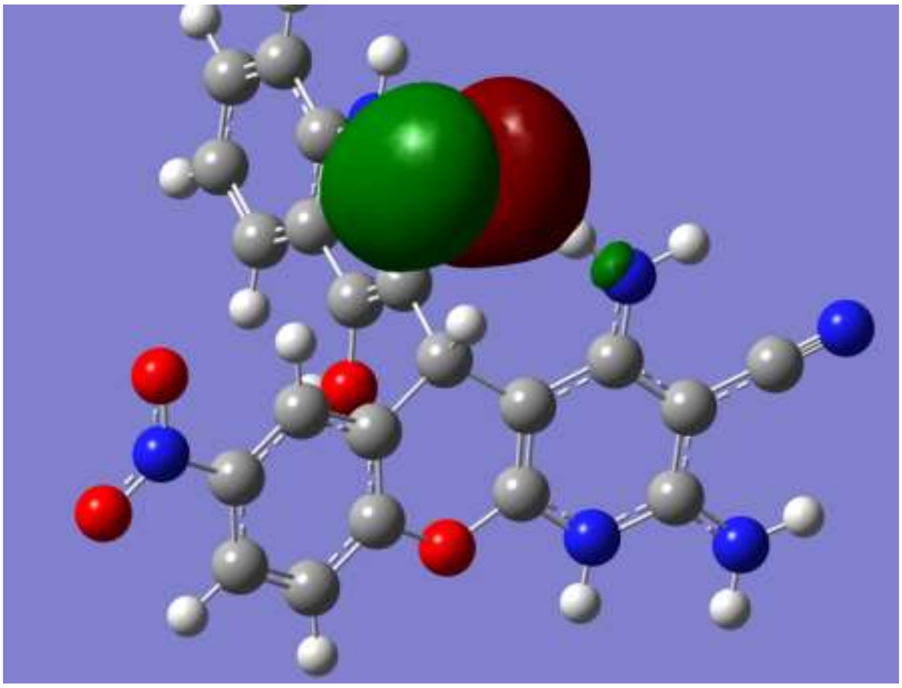

Figure S54. HOMO of **4h** (protonated compound)

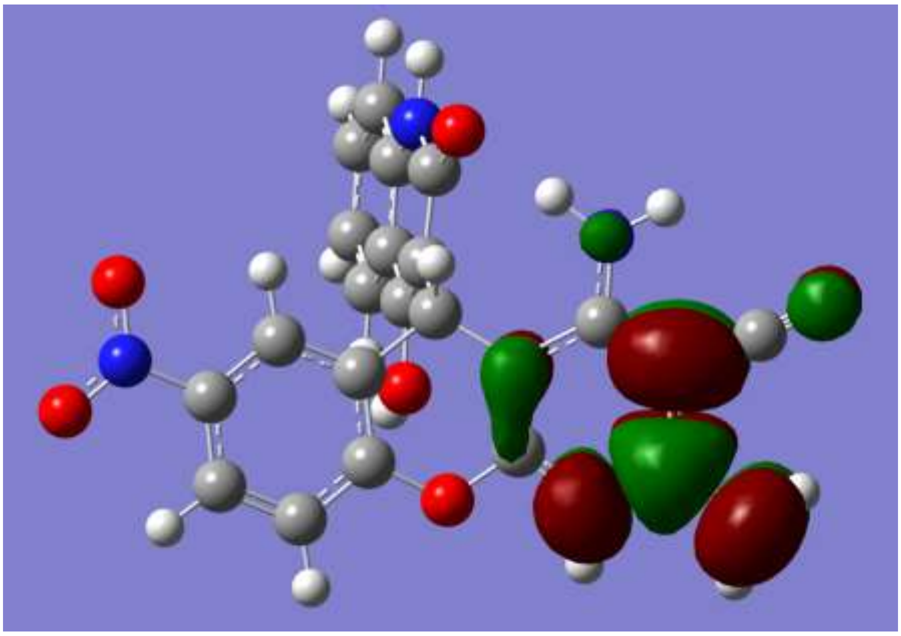

Figure S55. LUMO of **4h** (protonated compound)

References

[1] M.N. Elinson, A.N. Vereshchagin, Y.E. Anisina, A.N. Fakhrutdinov, A.S. Goloveshkin, M.P Egorov. (2019). *Eur. J. Org. Chem.*, 2019, 4171-4178.
